# Programmable synthesis of organic cages with reduced symmetry†

**DOI:** 10.1039/d4sc00889h

**Published:** 2024-04-01

**Authors:** Keith G. Andrews, Peter N. Horton, Simon J. Coles

**Affiliations:** a Department of Chemistry, Chemistry Research Laboratory, University of Oxford Oxford OX1 3TA UK; b Department of Chemistry, Durham University Lower Mount Joy, South Rd Durham DH1 3LE UK keith.g.andrews@durham.ac.uk; c UK National Crystallography Service, School of Chemistry, Faculty of Engineering and Physical Sciences, University of Southampton Southampton SO17 1BJ UK

## Abstract

Integrating symmetry-reducing methods into self-assembly methodology is desirable to efficiently realise the full potential of molecular cages as hosts and catalysts. Although techniques have been explored for metal organic (coordination) cages, rational strategies to develop low symmetry organic cages remain limited. In this article, we describe rules to program the shape and symmetry of organic cage cavities by designing edge pieces that bias the orientation of the amide linkages. We apply the rules to synthesise cages with well-defined cavities, supported by evidence from crystallography, spectroscopy and modelling. Access to low-symmetry, self-assembled organic cages such as those presented, will widen the current bottleneck preventing study of organic enzyme mimics, and provide synthetic tools for novel functional material design.

## Abstract 2

The rules underpinning the dynamic low-symmetry conformation of a symmetric organic cage are decoded, and rationally reapplied to access new cages with constrained symmetry. This allows tuning of conformation, height and shape for guest binding.

## Graphical abstract

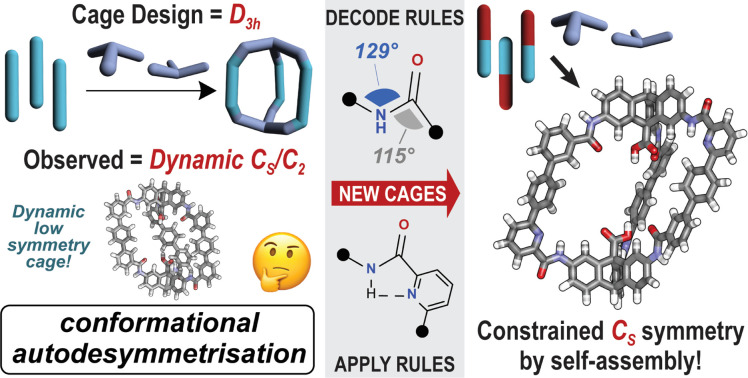

## 1 Introduction

It has long been known that supramolecular systems can host unique chemical environments not found in bulk solution or the gas phase.^1–7^ These tailored environments are highly attractive for tasks such as sensing,^8^ catalysis,^9,10^ separation,^11–13^ delivery,^14^ and stabilisation,^15,16^ to name a few. However, access to low symmetry cavities, such as those found in enzymes, remains challenging using current self-assembly approaches due to the reliance on symmetric geometries to favour assembly by dynamic covalent chemistry.^17–19^

Nonetheless, the successes of modern macromolecular cavity chemistry^8–11,14^ have inspired attempts to tune and reduce the symmetry elements of the cavities of self-assembled structures, to increase activity, selectivity and functionality.^20–25^ Promising cavity types include non-covalently assembled organic capsules,^26–28^ metal organic (coordination) cages^9,10,29–31^ and organic cages.^20,32–36^ While rational methods to lower symmetry in coordination cages have gathered increasing momentum,^21,23,37–44^ symmetry-lowering approaches in organic cages (usually imine-linked) remain opportunistic rather than procedural. In addition to the semi-stepwise methodology of Otte,^22^ one approach is to use computational screening combined with synthesis to assess viable formation of stable imine-linked cages when different types of multivalent aldehydes are mixed with multivalent amines.^45–48^ Social self-sorting, narcissistic self-sorting^49,50^ (including with chiral fragments)^47,51^ and scrambling are possible outcomes, and successful instances of reduced symmetry cages accessed *via* self-assembly have been reported by He and Zhang,^52^ Mukherjee,^50^ and Cooper, Slater, Greenaway and Jelfs.^47,53,54^ Although valuable, the outcomes are discovered^53,55,56^ rather than designed,^57^ and a lack of robust rationalisations of why certain cages are preferred means the search space for low symmetry cage geometries remains vast and poorly mapped. Further, without systematic access to incrementally varied cages, the correlation-rich structure–activity data that drives development towards application remains unavailable.

**Fig. 1:**
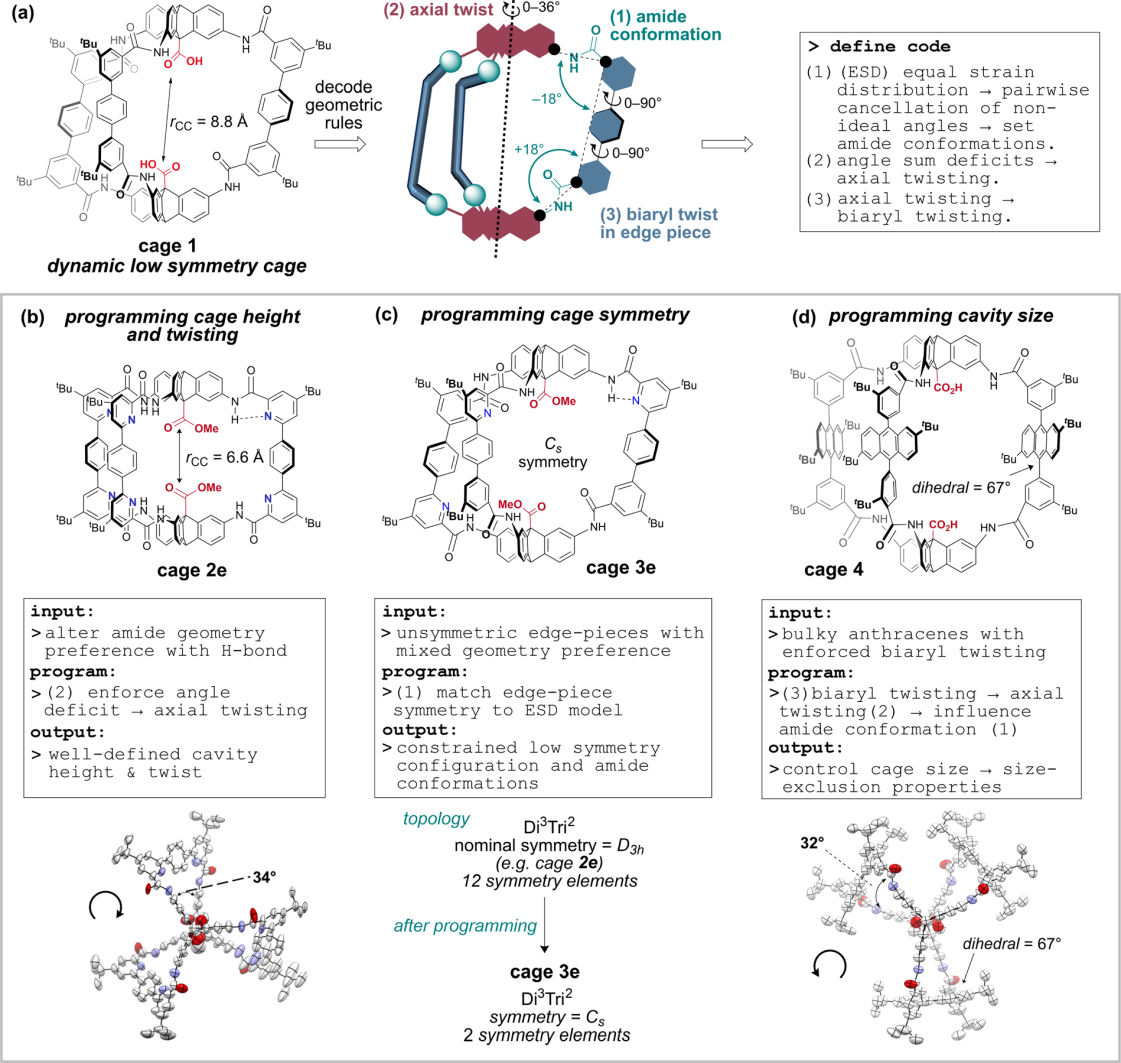
Programmable organic cage cavity tuning and symmetry lowering reported in this work. (a) Decoding the geometric rules underpinning the conformer landscape of cage 1 allows a set of codable rules to be defined. (b–d) Systematic exploitation of the conformational rules for programmable cage cavities with defined shape and reduced symmetry.

For this reason, our approach has been to tune specific promising cage classes based on amide-linkages, which offer greater stability and post-functionalisation^58–62^ options than the imine variants. To this end, we recently reported methodology to access robust, soluble and functional organic amide-linked cages^63^ using an *in situ* Pinnick oxidation locking approach,^64,65^ which advanced important work by Mastalerz.^61,66^ The resulting cages, which can be prepared on gram scale, are promising as sensors and catalysts as they feature a pair of endohedral antipodal carboxylic acid groups that resemble the enzyme motif found in a broad family of aspartyl proteases and glycoside hydrolases.^67,68^ The activity of these functional cages is expected to depend on cavity height, internal functionality, edge piece functionality, and edge piece steric presence, as well as symmetry. Towards our efforts to tune cavity properties, we now report a series of rational design principles that enable programable control of cavity shape, size, and symmetry (Fig. 1), supported by modelling, spectroscopy, crystallography, and exemplification of properties. Importantly, and unusually, we focus on the conformational preferences of the linking groups (amides) rather than the bonding vectors defined by the building block geometries. We first decode the geometric rules underpinning the amide conformational preferences for a dynamic low-symmetry cage, 1. We then demonstrate how rational exploitation of these rules gives access to geometrically well-defined low symmetry cavities.

## 2 Results and discussion

### 2.1 Defining the geometric code underpinning symmetry of cage 1

#### 2.1.1 Symmetric cage 1 presents a reduced-symmetry conformation

**Fig. 2:**
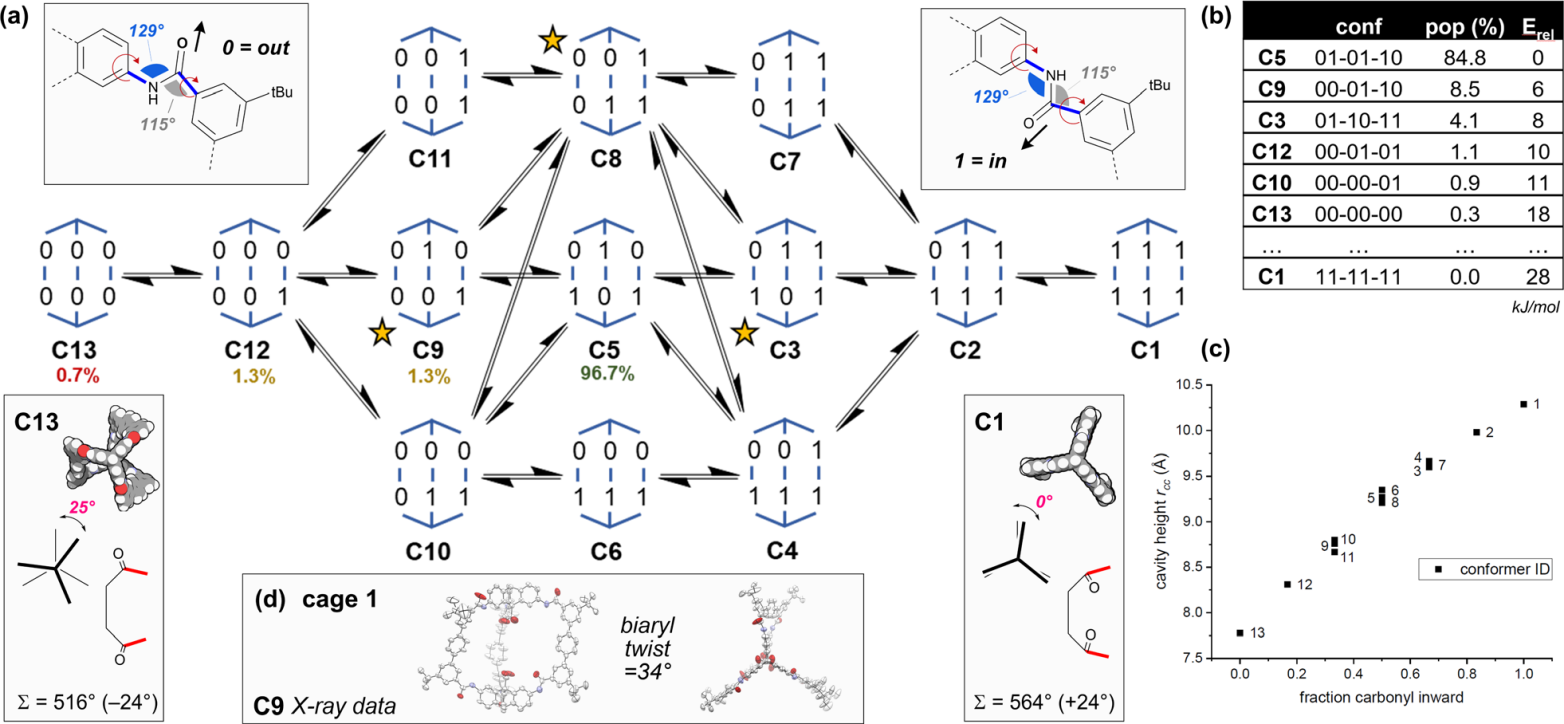
(a) The 13 conformers C1–C13 of cage 1 according to amide orientation [0 = carbonyl oxygen is oriented outwards; 1 = inwards] and their interconversion network. Their relative populations as calculated by MD simulations are shown as a percentage. Chiral conformers are marked with a star. (b) The table shows DFT energies (PBE0-D3BJ-def2-svp, CPCM(THF)) and Boltzmann weighted populations (298 K) for some of the conformers (see Tables S12–S15†). (c) The graph shows calculated cavity heights measured between the two carboxylic acid carbon atoms (*r*_CC_) for each conformer. Calculated axial twists are shown for C13 (left) and C1 (right), along with the internal macrocyclic angle sum, *Σ*, and the deviation (in brackets) from the ideal planar sum (540°). (d) Crystal structure data is shown for cage 1 (conformer C9).

In 2023, we reported the synthesis of the [2 + 3] hexaamide cage 1 by the *in situ* trapping of metastable imine assemblies.^63^ We now report the single crystal X-ray structure of diacid cage 1 (Fig. 2d). Although symmetric in design, cage 1, like its dimethyl ester analogue 1e,^63^ did not crystallise in the naively expected symmetric *D*_3h_ geometry that defines the trigonal prism cage topology^48^ (often termed [2 + 3] or Tri^2^Di^3^ cages^48^). In the crystal structures of both cages 1 and 1e, four of the six amide carbonyl groups are pointing out of the cage, with the remaining two, at the top and bottom of two separate edges, pointing inwards. This means both “symmetrical” achiral cages 1 and 1e display asymmetric chiral cavities in the solid state. We set out to understand this behaviour, believing it could underpin novel approaches to accessing reduced symmetry cages.

#### 2.1.2 The cavity of cage 1 is dynamic, but well-defined

In principle, any number of the six amide linkages could orient with the carbonyl oxygen pointing into or out of the cage – there are 13 unique permutations of six carbonyl orientations for planar^69^*trans*-amides, which we will refer to as conformations C1–C13 (Fig. 2a and Table S11†). Three are chiral (C3, C8 and C9).

DFT models (Fig. 2b and Tables S12–S15†) indicate C5 is predominant in solution (THF), with significant amounts of C9 (as seen in the X-ray structures of 1 and 1e) accessible at 298 K. Cage 1 undergoes dynamic exchange between conformers, supported by variable temperature (Fig. S17 and S18†) and NOESY ^1^H-NMR data (Fig. S30†), along with molecular dynamics simulations (nanosecond exchange) (Tables S17 and S18†). There are two crucial corollaries: first, the different conformers have vastly different cavity heights/properties and so access of specific conformers would allow tuning of the cavity height and symmetry (Fig. 2c). Second, the population of cage 1 is acutely weighted towards a few key structures in solution (Fig. 2a and b), which suggests that control of the conformation and therefore the symmetry of the cages is highly achievable. Thus, we sought to understand the natural bias towards low symmetry in cage 1 as a means to access tuned cavities through rational design.

#### 2.1.3 Cavity symmetry is a function of predictable geometric rules

Analysis of the conformers C1–13 in Fig. 2 and their energies revealed three key observations, which we codified as geometric rules underpinning cage behaviour (Fig. 1a).

(1) The low energy structures in Fig. 2 employ the amide conformational pattern 01–10–XX to maximally distribute strain. This is readily understood by noting that the amide bond linkages deviate from linearity: the CN̂C angle opens to 129.5°, whilst the NĈ(=O)C angle narrows to 114.8° (Fig. 2a)^69,70^ and so each terphenyl edge piece can project different bonding vectors depending on the conformation of the amide linkages (00, 01 and 11). Intuitively, “in–out” pairs of amides (01) cancel the angle deviations within an edge piece (Fig. 1a, centre). Likewise, up/down pairs of edges (01–10) cancel deviations between edges. Together, these allow the two triptycene caps to remain parallel, and accommodate a third edge piece. More generally, the sum of the internal angles of a convex 2D polygon with *n* sides (like benzene) must satisfy 
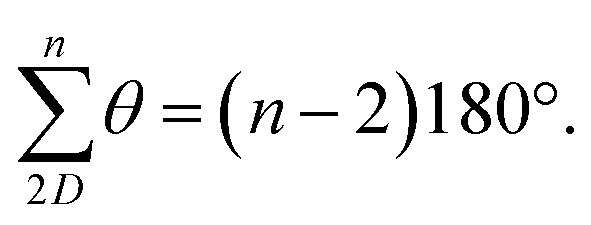
 The angle-pairing facilitated by conformers C5 and C9 satisfies this requirement, and so maximally distributes strain.

(2) Examining the DFT models, conformer C13 is axially twisted (25°) and energetically accessible, whilst C1 is untwisted (0°) and energetically inaccessible (Fig. 2, 2b). Geometry again explains this observation. Twisting a polygon into a third plane (*e.g.* benzene → cyclohexane), results in the inequality 
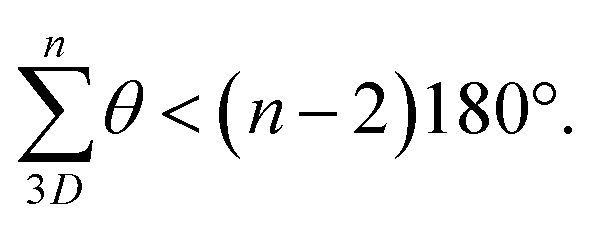
 This means planar polygon angle sum deficits (but not excesses) can be accommodated without bond angle strain if low energy twisting (out of plane) is possible. In the case of conformer C13, the six “out” amide linkages result in an angle sum deficit (−24°), which induces axial twisting (+25°) to relieve the bond angle strain. The angle excess in C1 cannot be accommodated, explaining its high energy.

Axial twisting bears its own cost: it requires a slight biaryl twisting in the terphenyl groups (Fig. 1a). This is tolerated, since the penalty for reducing conjugation within the terphenyl group by biaryl twisting from 34° (cage 1, C9) to 39° (cage 1, C13) is less costly than permitting bond angle strain from the angle deficit.^71,72^ Since cage height can be controlled by setting the cage conformation (Fig. 2c), the cage height can be programmed by enforcing the amide conformations, which also controls twisting (Fig. 1B).

(3) A final corollary is that the reverse process is possible: stabilising axial twisting can set the amide conformation. Since the strain from axial twisting can be dispersed in the edge-piece biaryl dihedral angles, it is possible to enforce biaryl twisting to reduce the cost of axial twisting, which alters the amide conformer preference and therefore the cage shape and size (Fig. 1D). We now exemplify these three rules to program cavity shape, size and symmetry. This new approach uses a dynamic system to solve geometric preferences, and then deploys rationally designed building blocks that reinforce the preferences to access stable cages with low symmetry.

### 2.2 Programming the cavity height using conformational rules

**Fig. 3:**
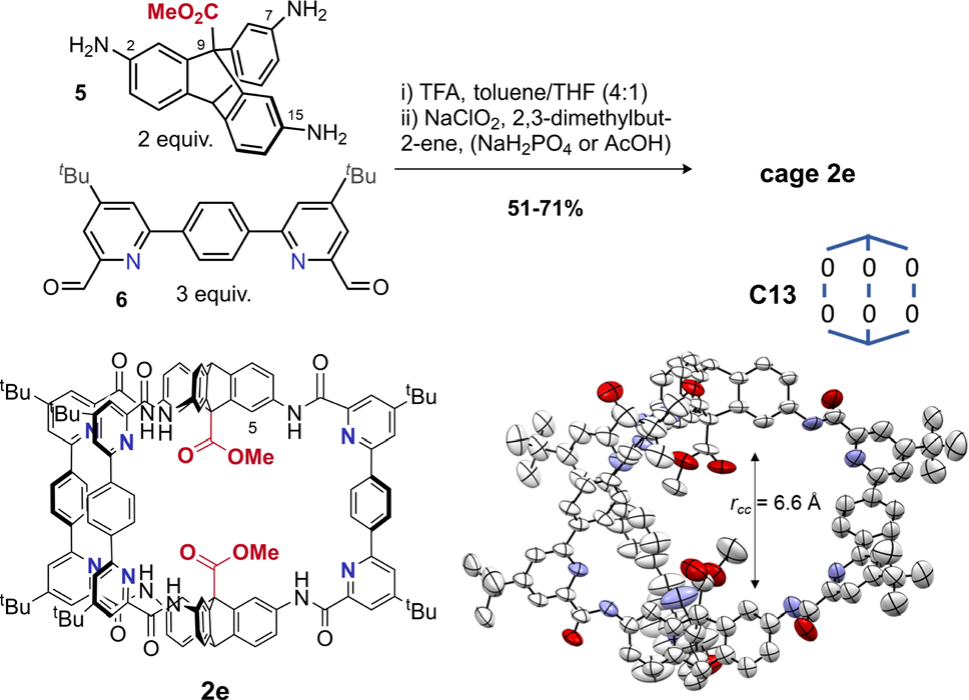
Synthesis of cage 2e and crystal structure showing the expected C13 conformer (see also Fig. 1b for the axial twisting).

Use of hydrogen bonding to override geometric preferences has been applied widely, from helical peptides^73^ and macromolecules^70,74^ to organic cages.^75^ We wondered whether cages containing pyridyl bisaldehyde 6 (Fig. 3) would orient the amide NH groups internally due to hydrogen bonding (or by reducing N/C=O dipole clashes).^70^ If so, then cage 2e would exist as C13, not C5, and the resulting angle deficit would illicit twisting, and decrease the cavity height. Accordingly, we synthesised tetrapyridine hexaamide [2 + 3] cage 2e in up to 71% yield from 5 and 6 using our previously developed *in situ* Pinnick oxidation strategy^63^ (Fig. 3). Cage 2e indeed crystallised in conformer C13, and shows a large twist angle of 34° (Fig. 1B and S21†) (*cf.*1e in C5 with no twist), and a significantly reduced cavity height of 6.6 Å (*cf.* cage 1e = 8.8 Å).^63^ NOE data shows exchange between the NH signal and the internal triptycene C**H̲**^5^, indicating that C13 predominates in solution for hexapyridine cages (Fig. S31†). Control of this acid–acid distance is of high interest in tuning the cage as a lysozyme mimic; we will report studies to this end separately. The ability to reliably install helicity may also allow cage chirality by induction.

### 2.3 Programming symmetry using conformational rules

**Fig. 4:**
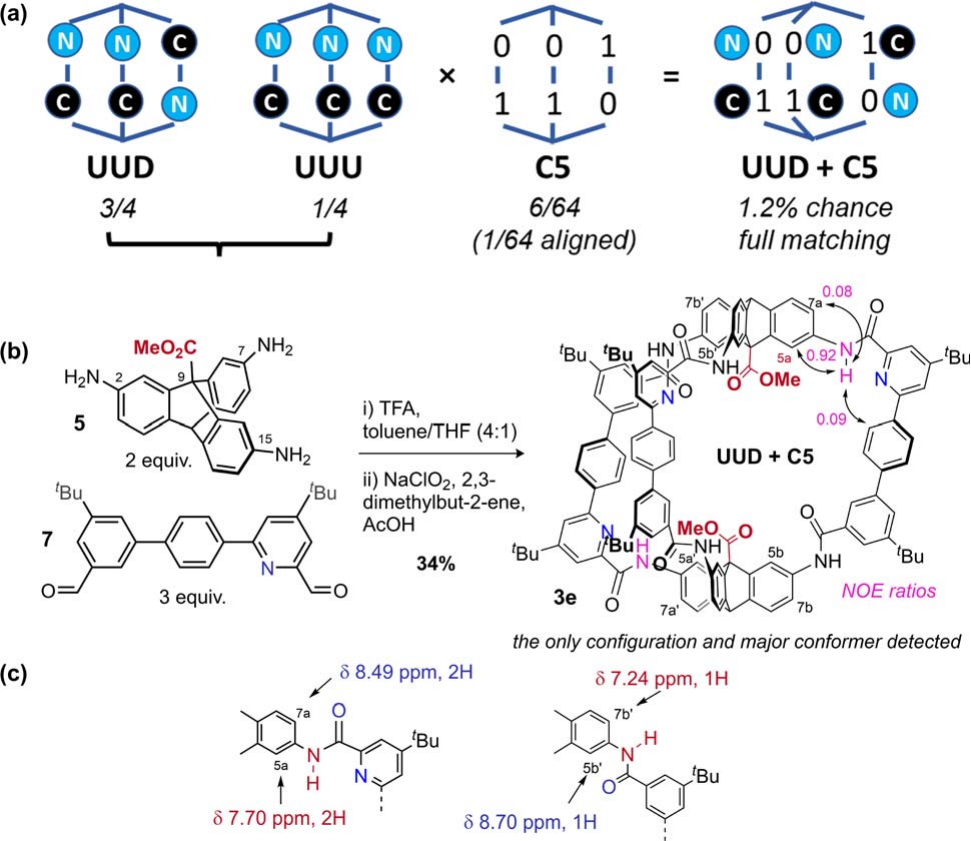
(a) Statistical permutations of configuration and conformation for [2 + 3] amide cages formed with an unsymmetric bisaldehyde. (b) Synthesis of cage 3e confirms the expected configuration and conformation according to the geometry heuristics discussed above. (c) ^1^H-NMR data (CDCl_3_) demonstrating the chemical shift effects of localised carbonyl orientation differences in cage 3e.

Recent work from Cooper, Jelfs, Slater, and Greenaway has focused on using computational screening to predict imine cage assembly.^53,55,76,77^ Using our geometry heuristics, we were able to design a low symmetry cage without further computation. Unsymmetric bisaldehyde 7, with one pyridyl aldehyde and one aryl aldehyde, can in theory form two [2 + 3] cages: all pyridine units can be adjacent to the same triptycene (“all up” = UUU: 1/4 chance), or one can be distal (“up–up–down” = UUD: 3/4 chance) (Fig. 4a).^50^ On the basis of our analysis of cage 1 (C5 preferred), and the observation that C13 is preferred for pyridyl cage 2e (pyridyl-directed amide orientation), we predicted formation of the cage with the UUD configuration and with cooperative matched pairs (C5 = 01–01–10) with “out carbonyls” adjacent to the pyridine units. The statistical (unbiased) probability of this outcome (UUD aligned with C5) is 1.2% across all possible configurations & conformations. When three equivalents of mixed bisaldehyde 7 were subjected to our assembly/oxidation cage protocol,^63^ we observed a single cage species and conformer in the crude NMR. Purification by recycling GPC provided a pure sample of cage 3e in 34% yield, which could be unambiguously assigned by ^1^H-NMR data as the expected UUD & C5 configuration and conformer. Notably, NMR data was consistent with the required *C*_s_ symmetry for UUD (Fig. S3–S7†) (2 : 1 signal ratios). Distinct and convincing ratios of NOE exchange between amide N**H̲** groups adjacent to the pyridine groups and either internal (5a, strong) or external (7a, weak) aryl-H environments were observed for both the UU (Fig. 4b) and D (Fig. S6 and S32†) environments, confirming that “*N*” environments were matched with “0” carbonyl out conformation. Additionally, the triptycene C**H̲** environments adjacent to a non-pyridyl aryl group showed reverse chemical shift trends compared to the analogous pyridyl-adjacent environments (Fig. 4c). Slight broadening of the environments near the non-pyridyl amide groups suggest amide rotation is limited to non-pyridyl amides, perhaps accessing minor amounts of C9 (and perhaps C12 & C13). We have been unable to obtain a suitable crystal for diffraction so far, as often observed for lower symmetry cages.^53^ Crucially, no UUU configuration was detected in the crude NMR. This self-sorting synthesis exploits the theoretical dynamic conformational symmetry-reduction process and translates it into a stabilised configurational low symmetry cavity.

### 2.4 Programming the cavity size using modular synthesis and steric engineering

**Fig. 5:**
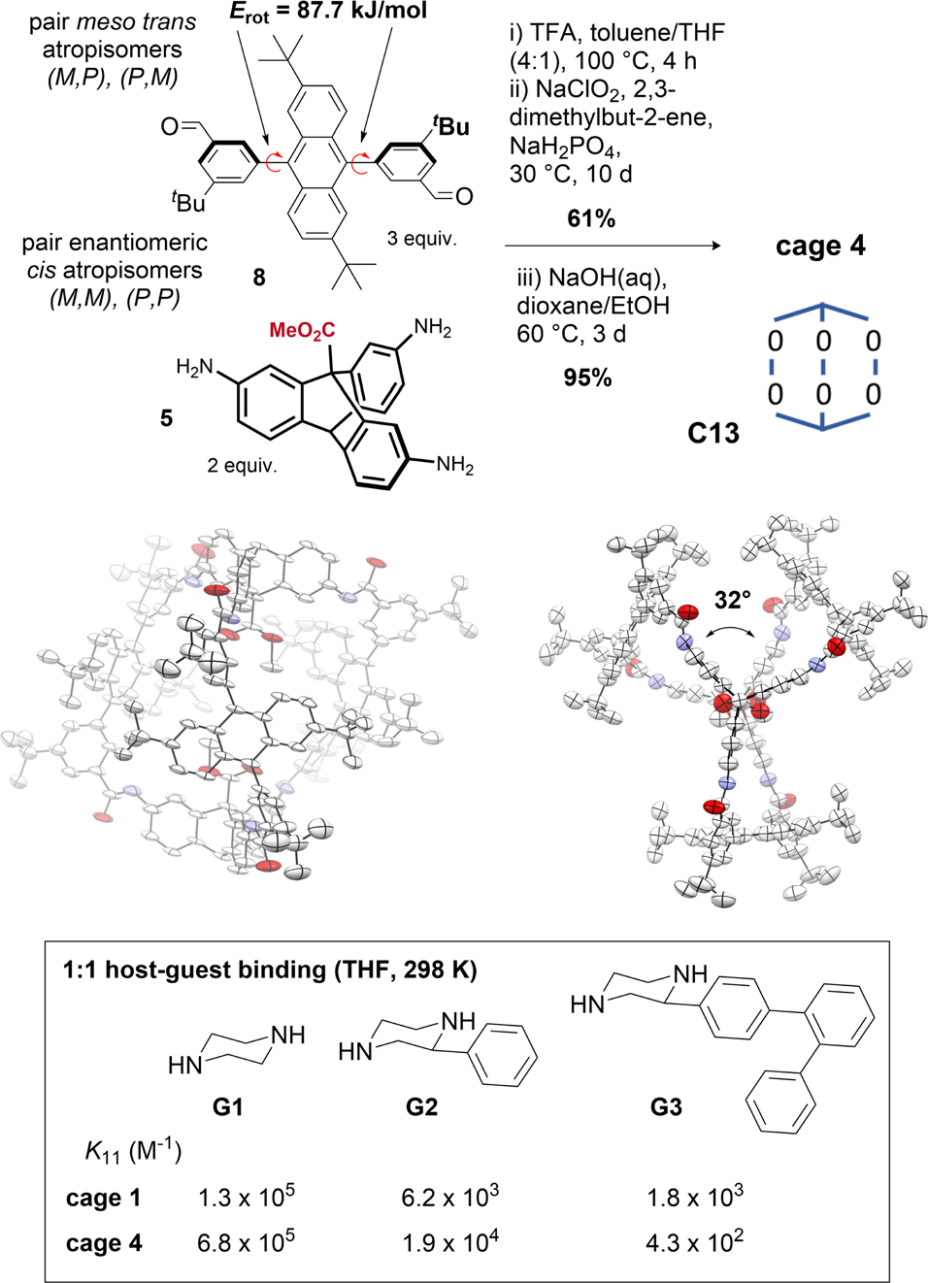
Synthesis of cage 4 depicting atropisomers and rotational barriers of bisaldehyde 8. Crystal structure of cage 4 (side view and top view, see also Fig. 1d) in conformer C13. Size exclusion properties of cage 4 compared to cage 1 demonstrated by comparison of 1 : 1 binding constants (298 K, THF) of bisamine guests.

Tuning of cage windows^78^ and cavity size and volume is a key technique for tuning cage properties.^77^ Modifications at the periphery typically alter the window size, although they can also influence cage topology.^79,80^ We sought to alter the cage cavity size by replacing the central aryl groups in the terphenyl edge pieces of cage 1 with 2,6-di-*tert*-butyl-anthracene groups. In solution (*e.g.* DMSO-d_6_, benzene-d_6_, d-chloroform), the anthracenyl bisaldehyde precursor 8 presented as *syn*/*anti* atropisomers (∼1 : 1) (Fig. S8†) with a rotational Gibbs energy barrier of 87.7 kJ mol^−1^, corresponding to a half-life on the order of minutes at 25 °C or milliseconds at 100 °C (Fig. S9 and Table S1†), indicating the *anti* atropisomer would not hinder cage self-assembly reactions requiring the *syn* geometry. The anthracene cage 4 was assembled as for cages 1–3, but the hexaimine formation was performed at 100 °C for 4 h to aid isomerization (Fig. 5). *In situ* Pinnick oxidation conditions afforded dimethyl ester cage 4e in 61% yield. Unlike 1e, complete methyl ester hydrolysis of 4e to give 4 required heating at 60 °C over 3 days with NaOH with added THF for full solubility, indicative that the cavity environments are very different (*cf.* 2 h at ambient temperature to hydrolyse cage 1e).^63^

Soluble anthracene cage 4 (>20 mg mL^−1^, THF) exists as two pairs of enantiomeric interconvertible atropisomers (Fig. S11†), accessed by 180° rotation of 1, 2, or 3 anthracenyl units from any starting point. Statistically, there are two equivalent configurations with *D*_3_ symmetry, and six with *C*_1_. ^1^H-NMR analysis indicates a roughly statistical mixture (2 : 6) of the two possibilities exists at equilibrium in THF at 298 K (Fig. S12 and S13†). The crystal structure shows only one conformer; C13 with ∼*D*_3_ symmetry, with a large axial twist angle of ∼32°, which appears to originate from the large dihedral angle between the anthracenyl groups and the flanking aryl groups (average dihedral angle = 67°). This means that aryl-anthracene conjugation is already reduced in the edge pieces, and so the cage no longer pays a biaryl twisting cost. Since axial twisting can promote favourable symmetry lowering, C13 predominates (in the solid state at least; solution phase behaviour may be more complex, see Fig. S33†). The axial twist may also be favoured due to a reduction in clashes between adjacent anthracene groups. In the crystal structure, the direction of the helical twist is determined by the axial atropisomeric configuration of the anthracenyl ^*t*^Bu groups. This indicates another mode of cavity symmetry-lowering, although we have yet to realise control over it: cages formed with stable (*M*,*M*) or (*P*,*P*) enantiomers of bisaldehydes like 11 are predicted to translate their axial configurational chirality to a conformational helical chirality.^42,47^ Instead, we were able to demonstrate the size-exclusion properties of cage 4 by observing a switch in binding preference for increasingly large bisamine guests (between the two carboxylic acids) at 298 K in THF relative to cage 1 (Fig. 5 and S14†).^63^ Cage 4 therefore highlights programmable cavity size exploiting a sequence of heuristics, although we note that rational control becomes more difficult when the factors contributing to the energy become more numerous and less distinctive. In these cases, control over solution-state preferences becomes more challenging.

## 3 Discussion

Many current approaches to access low-symmetry cages use geometrically unsymmetric edge pieces.^53,80^ The aldehydes used in the current work are all geometrically symmetric – their bonding vectors and steric requirements are equivalent. Yet, within the amide cages, they undergo a symmetry reduction. The observation that a symmetric assembly can relax into a reduced symmetry conformation is not new, but instances are usually “noted” rather than exploited.^50,53,81–84^ The observed symmetry-lowering here is a result of the linker design within the cage polymacrocycle system; the same preferences do not necessarily exist outside of the cage context. Inside a polymacrocycle, the angle sum requirements compel competing preferences to “rank themselves” to equally distribute strain.^85,86^ This can cause symmetry lowering in a way not available for fixed bonding vectors.

“Self-sorting” describes the configurational assembly preference of components in a self-assembling mixture.^87,88^ Although self-sorting inherently includes conformational biases, symmetry is usually defined by the configuration. In the current work, conformational preferences can affect the symmetry independently to configuration (*e.g.* cage 1). In our synthesis of cage 3e, we harness this conformational preference to drive configurational self-sorting.

We therefore emphasise that the technique discussed here should be viewed as a rational approach to achieve low-symmetry assemblies using self-assembly synthesis, rather than merely an observation implying the symmetric structure is strained. Stated clearly, the concept is this: assemblies based on symmetric polyhedra can be biased to access non-symmetric conformational minima by incorporation of motifs in which strain cannot be symmetrically distributed (in the polymacrocycle environment). We tentatively term this, as yet unnamed, approach: “conformational autodesymmetrisation”.

## 4 Conclusions

We have exemplified programmable cavity tuning and symmetry-lowering of amide-linked organic cages using heuristics derived from conformational analysis to access three new cage architectures. By subtle modification of the bisaldehyde edge-piece fragments in the three cages, we were able to tune the amide conformational preferences, which are intimately coupled to the cage axial twist and edge-piece biaryl twist angle. These parameters in turn define the cage height, symmetry, and volume. In essence, we decoded the geometric preferences of a dynamic cage and applied them to access cages with well-defined geometries with reduced symmetry. Notably, we were able to reduce the symmetry of a *D*_3h_ [2 + 3] cage architecture to *C*_s_ symmetry by using a conformational autodesymmetrisation approach, in which building blocks are selected to generate an assembly in which strain cannot be symmetrically distributed. The results are supported by crystallography data and NMR assignments, which demonstrate strong conformational preferences in solution for cages 2e and 3e. The protocols reported here represent important advances in tailored cage synthesis, and will lead to methods to access robust, chiral cages, with controllable flexibility, and internal functionality mimicking enzyme motifs.

## Data availability

All experimental/computational procedures and data related to this article and additional reference annotations are provided in the ESI.†

## Author contributions

KGA conceived, managed, and executed the project. KGA performed the experiments, computations and analysis. PNH and SJC collected and solved the single crystal X-ray diffraction data. KGA wrote the manuscript, with input from all authors.

## Conflicts of interest

There are no conflicts to declare.

## Supplementary Material

SC-015-D4SC00889H-s001

SC-015-D4SC00889H-s002

SC-015-D4SC00889H-s003
