# Supplementary material for: Programmable synthesis of organic cages with reduced symmetry
Source: Chem Sci. 2024 Apr 1;15(17):6536–43. doi: 10.1039/d4sc00889h (PMC11062111; doi:10.1039/d4sc00889h)
Supplement: SC-015-D4SC00889H-s001 [file SC-015-D4SC00889H-s001.pdf]

Electronic Supplementary Material (ESI) for Chemical Science.
This journal is © The Royal Society of Chemistry 2024

Supporting Information for:

Programmable Synthesis of Organic Cages with Reduced Symmetry

Keith G. Andrews,* Peter N. Horton, Simon J. Coles

Contents

General Methods 4
Synthesis 5
Synthesis of triptycene **5** 5
Triptycene **5** 5
Synthesis of bisaldehydes **s13**, **6** and **7** 7
Bisaldehyde **s13** 7
Bisaldehyde **6** 8
Figure S2. Synthesis of choloropyridine **s16** 8
Bisaldehyde **7** 10
Synthesis of cages **1**, **2e** and **3e** 11
Synthesis of cage **1** 11
Synthesis of cage **2e** 12
Synthesis of cage **3e** 13
Analysis of cage **3e** 14
Figure S3. ^{1}H-NMR spectra ($CDCl_3$) of cage **3e** 14
Figure S4. ^{13}C-NMR spectra of cage **3e** 15
Figure S5. 2D-EXSY experiments 15
Figure S6. Cage **3e** NOE analysis 16
Figure S7. NMR analysis demonstrates that the UUD configuration and C5 conformer are the only plausible candidates for the identity and major conformation of cage **3e**. 16
Synthesis of the anthracene bisaldehyde **8** 17
Figure S8. Depiction of the atropisomers of anthracenyl bisaldehyde **8** 19
Figure S9. Variable temperature (25-112 °C) ^{1}H-NMR spectra 19
Table S1. Calculation of rotational barrier of bisaldehyde **8** 20
Synthesis of anthracene cage **4** 21
Hydrolysis of anthracene cage **4e** to form **4** 22
Figure S11. Depiction of the possible atropisomers of tert-butyl anthracene units in cage **4e**. 22
Figure S12. Selected regions of ^{13}C-NMR spectra of cage **4** displaying evidence of the two isomers. 23
Figure S13. Selected regions of ^{1}H-NMR spectra of cage **4** displaying evidence of the two isomers. 24
Binding titrations of cages **1** and **4** with bisamines 25

Synthesis of guests for binding titrations 25

^{1}H-NMR Binding Titration Experiments - Protocol 26

Titration Binding Data Summary 27

Table S2. Titration binding data for cage **1** with **G1-3** fitting to a 1:1 model. Host concentration fitting allowed as indicated. 28

Table S3. Titration binding data for cage **1** fitting to a 1:2 (host:guest) model. Host concentration fitting allowed for strongly binding guests. K_1 and K_2 are stepwise association constants. 28

Table S4. Titration binding data for cage **1** fitting to a 1:1 (host:guest) model. Ligand concentration fitting performed for all guests. [host concentration calculated by internal standard]. 29

Table S5. Titration binding data for cage **1** fitting to a 1:2 (host:guest) model. Ligand concentration fitting performed for all guests. [host concentration calculated by internal standard]. 29

Table S6. Titration binding data for anthracene cage **4** fitting to a 1:1 (host:guest) model. Host concentration fitting allowed for strongly binding guests. 30

Table S7. Titration binding data for anthracene cage **4** fitting to a 1:2 (host:guest) model. Host concentration fitting allowed for strongly binding guests. 30

Table S8. Titration binding data for anthracene cage **4** fitting to a 1:1 (host:guest) model. Ligand concentration fitting performed for all guests. [host concentration calculated by internal standard]. 31

Table S9. Titration binding data for anthracene cage **4** fitting to a 1:2 (host:guest) model. Ligand concentration fitting performed for all guests. [Host concentration calculated by internal standard]. 31

Figure S14. ^{1}H-NMR binding constant data 32

Example of titration data, fitted values, and chemical shift changes 33

Figure S15. Example ^{1}H-NMR host-guest binding titration data 33

Figure S16. Fitting the triptycenyl bridgehead proton to the 1:1 model. 34

Conformational studies of cage **1** 35

Demonstration of cage **1** conformer permutations – **C1-C13** 35

Table S11. Conformer label assignment of cage **1**, **C1-C13** 36

Boltzmann weighting 36

Variable Temperature ^{1}H-NMR (VT-NMR) of cage **1** – **Figure S17, S18** 37

Crystallisation Methodology 38

Figure S19. Crystallography details for cage **1** 38

Figure S20. Intramolecular hydrogen bond contacts for cage **1**. 38

Figure S21. Crystallography details for cage **2e.** 39

Figure S22. Crystallography details for cage **4e.** 39

DFT study of geometry of cage **1** 40

Empty cage generation, conformational screening and optimisation 40

Tables S12-15: cage **1** parameters generated using different DFT functionals 40

Figure S23. Connectivity map showing which cage conformers are connected 42

Molecular Dynamics of cage **1** 43
Figure S24 45
Table S16. Example of cage conformational data scraping from MD file. 46
Table S17. Molecular dynamics: 13 x 1 µs simulations of cage **1** 46
Table S18. Molecular Dynamics populations. 49
Cage Conformer Molecular Dynamics Transition Pathways 51
NOE data – **Figures S28-33** 53
NOE data for cage 1e 53
NOE data for cage 1_{HH} 54
NOE data for cage 1 55
NOE data for cage 2_{HH} 56
NOE data for cage 3e 57
NOE data for cage 4 58
References for supporting information 59
Spectra 61
Reference Annotations for main manuscript 76

General Methods

Commercially available reagents were used as received. Dry solvents (THF (tetrahydrofuran), CH_2Cl_2, benzene, diethyl ether) for reactions were purified by a MBraun MB-SPS-5 bench-top SPS system under nitrogen (H_2O content < 20 ppm). All other solvents used were HPLC grade and dried over appropriate drying agents when required. Petroleum ether (petrol) had a boiling point range of 40–60 °C. TFA = trifluoroacetic acid. All solutions used during workups ($NaHCO_3$, brine) were saturated aqueous solutions, unless otherwise specified. Reactions, unless otherwise stated, were carried out in undried glassware under an air atmosphere. Thin layer chromatography (TLC) was carried out on aluminium-backed silica gel plates with 0.2 mm thick silica gel 60 F254 (Merck) and visualized by UV irradiation at either 254 nm or 366 nm. Preparative flash column chromatography was either carried out using flash silica gel 60 (230-400 mesh) obtained from Sigma-Aldrich, or on a Biotage Isolera One with a 200–400 nm UV detector using sfar or KP-sil prepacked columns ("flash cartridges"). Size exclusion chromatography (SdEC) was carried out using Bio-Beads S-X3, 40-80 µm (Bio Rad). Evaporation of solvents was performed at 20–50 °C and 5–1010 mbar. Reported yields refer to pure compounds dried under high vacuum (< 0.1 mbar). ^{1}H and ^{13}C nuclear magnetic resonance (NMR) spectra were recorded on Bruker AVIII HD 400, Bruker NEO 600, Bruker AVIII HD 500, Bruker AVIII HD 600 (Prodigy N2 broadband cryoprobe) spectrometers at 400 MHz, 600 MHz, 500 MHz, and 500 MHz (^{1}H) and 101 MHz, 151 MHz, 126 MHz and 126 MHz (^{13}C), respectively at 298 K unless stated otherwise. NMR chemical shifts were reported in ppm relative to $SiMe_4$ ($\delta = 0$) and were referenced internally with respect to residual solvent protons using the reported values. All chemical shifts are reported in ppm, coupling constants are reported in Hz and ^{1}H multiplicities are reported in accordance with the following: app= apparent; s = singlet; br s = broad singlet; d = doublet; t = triplet; q = quartet; and m = multiplet. ^{1}H assignments were made using 2D NMR methods (COSY, NOESY, HSQC, HMBC). Electrospray mass spectrometry was carried out on a Waters Micromass LCT Premier XE spectrometer using 90:10 $MeOH:H_2O$ (+0.1% formic acid) as the mobile phase. High-resolution mass spectrometry (HR-MS) measurements were performed by the mass spectrometry service at the University of Oxford on a Waters GTC classic. MALDI measurements were performed using a Bruker Autoflex Speed MALDI-ToF using a DCTB matrix (*trans*-2-[3-(4-tert-butylphenyl)-2-methyl-2-propenylidene]malononitrile, CAS=300364-84-5). Computational calculations were performed using the High Performance Computing service from Advanced Research Computing,[1] running Linux CentOS 8. Desktop computing was performed on Windows 10, with python scripts run using OS X 10.14.6. OpenBabel (v2.4.0, Nov 2021)[2] was used to convert chemical file types, in particular Gaussian log files to .xyz files. AutoDE[3] was used for handling structural information with python scripts, and calculating distances/angles etc.

Synthesis

Synthesis of triptycene **5**

*Triptycene **5***

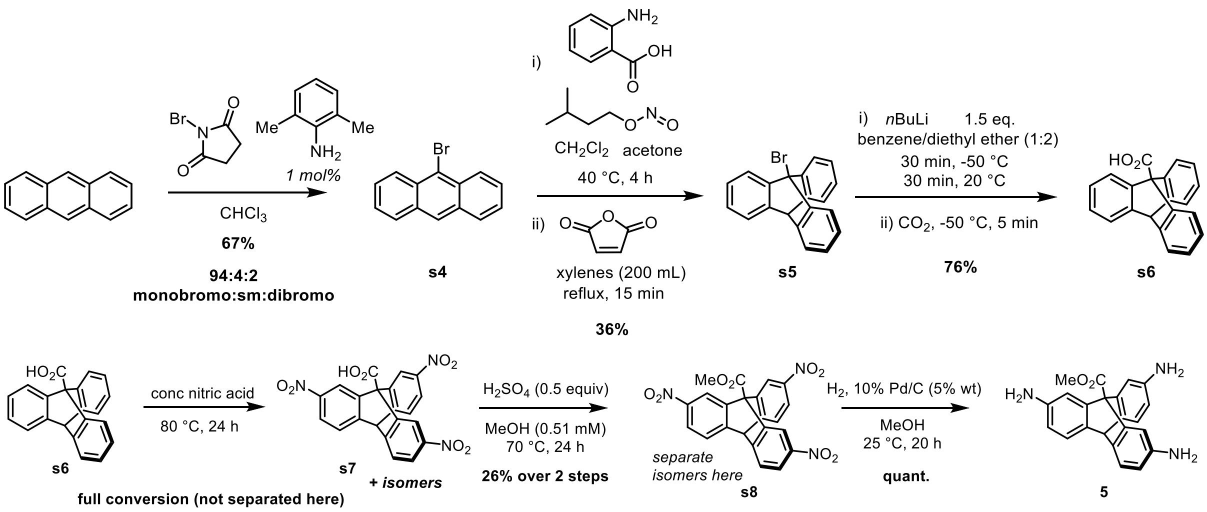

Figure S1. *Synthesis of triptycene **5**.*

9-bromoanthracene (s4): According to an adapted literature procedure,[4] anthracene (36.0 g, 202 mmol) was dissolved in dry dichloromethane (750 mL). Separately, *N*-bromosuccinimide (NBS) was partially dissolved in a mixture of dry dichloromethane (150 mL) and chloroform (50 mL), and pyridine (0.5 mL) was added to attempt to assist dissolution with limited success. Added to each mixture was 2,6-dimethylaniline (144 µL; 248 µL in total, 2.02 mmol,). The solution of anthracene was cooled to –40 °C, and the solution of NBS was then added portionwise over 3 h, stirring in the dark. The reaction was stirred for a further 14 h, warming to ambient temperature, and then concentrated. The resulting dark brown sugar-like solid was triturated with a mixture of pentane and dichloromethane (1.2 L, 9:1), and the succinimide by-product filtered off. The remaining solution was concentrated, and the solid analysed by ^{1}H-NMR, suggesting a ratio of 4:94:2 anthracene:monobromo:dibromo. Attempts to separate this mixture by recrystallisation from boiling hexane or column chromatography with neat petrol proved fruitless on this scale, and it is preferred to take the material to the next step after a quick silica pad filtration with neat petrol to leave behind darker residues (45 g, 87%); the crude residue has major signals consistent with the literature:[5] **^{1}H NMR** (400 MHz, $CDCl_3$) δ 8.53 (app. ddd, *J* = 8.9, 1.8, 1.1 Hz, 2H), 8.45 (s, 1H), 8.01 (app. ddd, *J* = 8.0, 1.8, 1.3 Hz, 2H), 7.61 (ddd, *J* = 8.9, 6.6, 1.3 Hz, 2H), 7.51 (ddd, *J* = 8.0, 6.6, 1.1 Hz, 2H).

9-bromotriptycene (s5): According to adapted literature procedures,[6,7] 9-bromoanthracene **s4** (34.90 g, 135.7 mmol) was dissolved in dry dichloromethane (400 mL) in an oven-dried 3-necked 1 L round-bottomed flask fitted with a water condenser and a 250 mL pressure equalised dropping funnel. To this refluxing solution (42 °C), under a flow of argon, was added dropwise from the dropping funnel over 4 h a solution of anthranilic acid (18.61 g, 135.7 mmol) in acetone (110 mL), at a rate to maintain a gentle bubbling. The solution became orange and then brown after 1.5 h. After completion of the addition, the reaction was stirred at reflux for a further hour, and then concentrated to ~ 50 mL, and redissolved in xylenes (200 mL). Maleic anhydride (20.0 g, 102 mmol) was dissolved in a minimum of acetone and added to the mixture, and the reaction stirred at reflux (155 °C) for 30 min. After this time, the reaction was cooled for 10 min, and then poured into cold water (500 mL) and diluted with dichloromethane (200 mL), and the mixture stirred at room temperature overnight. The red-brown organic layer was diluted with further dichloromethane, and washed four times with 15% wt sodium hydroxide solution, and the coloured aqueous layers discarded. The remaining organic phase was concentrated to dryness, and

then refluxed in ethanol (~800 mL), the undissolved solid filtered hot (11.85 g of **s5**), and the resulting solution cooled, and a further crop of light brown crystals filtered (4.2 g). The combined solids were washed with ice cold ethanol to leave small tan crystals of **s5** (16.05 g, 36%) with data matching the literature:[7,8] **^{1}H NMR** (400 MHz, $CDCl_3$) δ 7.81 – 7.78 (m, 3H), 7.40 – 7.36 (m, 3H), 7.10 – 7.03 (m, 6H), 5.43 (s, 1H); **^{13}C NMR** (101 MHz, $CDCl_3$) δ 144.4, 143.7, 126.4, 125.5, 123.9, 123.1, 71.5, 53.8.

triptycene-9-carboxylic acid (s6): 9-Bromo-triptycene **s5** (5.00 g, 15.0 mmol) was dissolved in a mixture of benzene (250 mL) and diethyl ether (500 mL) and the reaction cooled to –50 °C. Added dropwise was ^{n}BuLi (14.1 mL, 1.6 M in hexanes, 1.5 equiv.) and the reaction stirred for 30 min, and then for a further 30 min at room temperature to yield a brown/tan suspension. The reaction was recooled to –50 °C, and dry gaseous carbon dioxide rigorously bubbled through the solution for 10 minutes. Ideally, the tan suspension should become a clear solution in less than 2 minutes. [NOTE: To generate the CO_2, a dry flask fitted with a drying tube ($CaSO_4$, Drierite), rubber tubing and a cannula on an adapted syringe was charged with solid (dry ice) carbon dioxide. At the requisite time, the cannula was inserted into the reaction solution through a Suba-Seal (rubber cap), (with a vent to a Schlenk line) and the flask of dry ice submerged in a water bath at room temperature to generate a rapid flow of gaseous CO_2] (warning: pressure!). After 10 min of CO_2 bubbling, the reaction was concentrated to 500 mL *in vacuo*, then quenched with a saturated aqueous ammonium chloride solution, the organics separated, and the aqueous layer extracted with diethyl ether. The combined organics were dried over magnesium sulfate, filtered and concentrated. The resulting solid was purified by flash column chromatography (dry loaded, biotage KP-Sil 100 g cartridge) with a gradient of 0-50% petrol/ethyl acetate to remove the parent triptycene and finally eluting the acid with 80% ethyl acetate/petrol to give **s6** as a white solid (3.20 g, 71%) with data consistent with the literature:[9] **^{1}H NMR** (400 MHz, $CDCl_3$) δ 7.87 (dd, J = 5.6, 3.3 Hz, 3H), 7.43 – 7.38 (m, 3H), 7.08 – 7.00 (m, 6H), 5.39 (s, 1H).

2,7,15-trinitrotriptycene-9-carboxylic acid ($s7_{mix}$)): Triptycene-9-carboxylic acid **s6** (3.20 g, 10.7 mmol) was suspended in nitric acid (20 mL) and the reaction heated with stirring at 80 °C for 20 h. After this time, the reaction was cooled to 40 °C and poured into 150 mL water with stirring. The resulting precipitate/solution was filtered through the same filter bed repeatedly until the filtrate was a clear liquid, and then the solid was carefully washed with water. The solid was dissolved in ethyl acetate and washed with brine (containing 5% sodium bicarbonate), dried over magnesium sulfate, filtered and concentrated to give a crude solid **$s7_{mix}$** (4.65 g, quant.) as a mixture of isomers, as described in the literature.[9] The solid is slightly soluble in chloroform, and more soluble in acetone. The material is best taken to the next step crude as a mixture of isomers. The crude material has data: **^{1}H NMR** (400 MHz, $CDCl_3$) δ 8.86 – 8.78 (m, 3H), 8.21 – 8.05 (m, 3H), 7.68 – 7.57 (m, 3H), 5.80 – 5.74 (m, 1H).

methyl 2,7,15-trinitrotriptycene-9-carboxylate (s8): The mixture of isomers **$s7_{mix}$** (4.65 g, 10.7 mmol) was dissolved in methanol (60 mL) and added was concentrated sulfuric acid (1.7 mL) and the reaction stirred at reflux (70 °C) for 20 h. Progress was followed by ^{1}H-NMR. [**NOTE:** the concentration of substrate and acid is important to obtain the reported rate. Ideally, MeOH is at least 2 mL/mmol substrate, and sulfuric acid is at least 30 µL/mmol substrate.] On completion, the reaction was cooled and diluted with ethyl acetate, and washed with saturated sodium bicarbonate solution and brine, dried over magnesium sulfate, filtered and concentrated. The crude residue was purified by flash column chromatography (loaded (in ethyl acetate) onto a 340 g KP-SNAP biotage cartridge) and eluted using a gradients of 0-22% (hold for first isomer, δ H (Me) = 4.37 ppm); 22-25% (hold for major isomer, δ H (Me) = 4.40 ppm); 25-45% (hold for desired isomer, δ H (Me) = 4.43 ppm). The desired isomer **s8** was isolated as a white solid (900 mg, 19% over two steps) with data consistent with the literature.[9] **^{1}H NMR** (400 MHz, $CDCl_3$) δ 8.64 (d, *J* = 2.1 Hz, 3H), 8.09 (dd, *J* = 8.2, 2.1 Hz, 3H), 7.64 (d, *J* = 8.2 Hz, 3H), 5.76 (s, 1H), 4.43 (s, 3H). **^{13}C NMR** (126 MHz, $CDCl_3$) δ 168.0 (CO_2R), 149.1, 146.4, 143.2, 125.0, 123.0, 120.3, 61.5 (C(CO_2Me), 53.9 (CH), 53.7 (OMe). **AT-IR** (neat, cm^{-1}): 2981, 1746, 1603, 1523, 1342.

methyl 2,7,15-triaminotriptycene-9-carboxylate (5): Trinitrotriptycene **s8** (115 mg, 0.257 mmol) was dissolved in methanol (5 mL) and under argon was added 10% Pd/C (6 mg, 5% wt/wt). The reaction was stirred vigorously overnight under an atmosphere of hydrogen (double balloon), purged to an atmosphere of argon, filtered through Celite (ensuring the Pd residue did not dry out, and finally quenching the Pd with water once the filtration was complete) and concentrated to dryness to give **5** as an off-white solid (95.5 mg, quant), with data consistent with the literature.[9,10] **^{1}H NMR** (400 MHz, $CDCl_3$) δ 7.06 (d, J = 7.8 Hz, 3H), 7.04 (d, J = 2.2 Hz, 3H), 6.31 (dd, J = 7.7, 2.2 Hz, 3H), 5.01 (s, 1H), 4.18 (s, 3H); **^{13}C NMR** (101 MHz, $CDCl_3$) δ 171.1, 144.2, 143.4, 137.7, 123.4, 112.1, 111.7, 62.1, 52.0, 51.9; **MS *m/z*** (ESI+): 358.2 ($C_{22}H_{20}N_3O_2$, $[M+H]^+$ requires 358.16); **AT-IR** (neat, cm^{-1}): 3346, 2952, 1731, 1603, 1474, 1298.

Synthesis of bisaldehydes **s13**, **6** and **7**

*Bisaldehyde **s13***

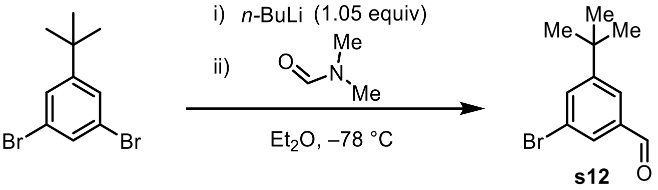

3-bromo-5-(tert-butyl)benzaldehyde s12: 1,3-Dibromo-5-(tert-butyl)benzene (5.00 g, 17.1 mmol) was dissolved in ether (40 mL) and cooled to –78 °C (dry ice/acetone bath). Added dropwise over 3 min was *n*-butyllithium (1.6 M solution in hexanes, 1.05 eq, 11.3 mL, 18.0 mmol). After stirring for 30 min at –78 °C the solution was allowed to warm to –30 °C over 30 min. Added dropwise at –30 °C was dimethylformamide (1.99 mL, 1.5 eq, 25.7 mmol), and then the reaction was allowed to warm to 0 °C over 2.5 h. After this time, the reaction was quenched with aqueous ammonium chloride solution, and extracted with ethyl acetate. The combined organics was washed with water and brine, and then dried over magnesium sulfate, filtered and concentrated. The crude residue was loaded onto a 100 g (sfar liquid, biotage) column in a minimum of petrol, and purified by flash column chromatography (petrol/Et_2O) (0 to 12%) to yield a pale yellow oil **s12** (2.52 g, 61%); **^{1}H NMR** (400 MHz, $CDCl_3$) δ 9.95 (s, 1H), 7.82 (d, J = 1.7 Hz, 2H), 7.77 (d, J = 1.8, 1H), 7.77 (d, J = 1.8, 1H), 1.35 (s, 9H); **^{13}C NMR** (101 MHz, $CDCl_3$) δ 191.3, 154.8, 138.0, 134.8, 130.0, 125.5, 123.4, 35.3, 31.2; **HR-MS *m/z*** (ESI+): 241.0211 ($C_{11}H_{14}BrO$, $[M+H]^+$ requires 241.0223).

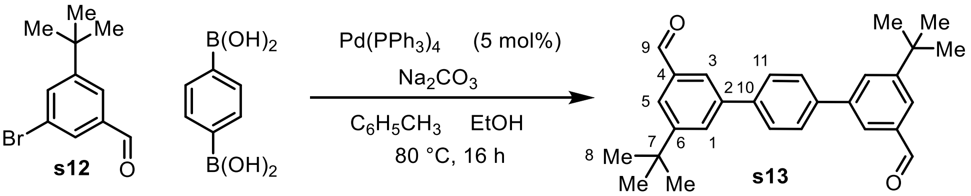

Bisaldehyde s13: Aryl bromide **s12** (1.50 g, 6.22 mmol) was dissolved in toluene (20 mL) and ethanol (7 mL) with tetrakis(triphenylphosphine)palladium(0) (180 mg, 5 mol%) and diboronic acid (516 mg, 3.11 mmol) and added was sodium carbonate (1.17 g, 3.54 mmol) predissolved in water (20 mL) and the reaction stirred under argon for 16 h at 80 °C. Two further portions (50 mg) of diboronic acid were added after 16 h and 20 h, and the reaction was judged complete on the appearance of “palladium black” at 20.5 h. The reaction was cooled, diluted in ethyl acetate and washed with sodium hydroxide and brine, dried over magnesium sulfate, filtered and concentrated. The crude material was solid-loaded onto silica, and purified by flash column chromatography (0-15% EtOAc in petrol, KP-Sil 50 g column, biotage) to give **s13** as a white powder (1.00 g, 81%) with data consistent with the literature:[10] **^{1}H NMR** (400 MHz, $CDCl_3$) δ 10.11 (s, 2H), 7.97 (dd, J = 1.6, 1.6 Hz, 2H), 7.93 (2xoverlapping ap. d, J = 1.6 Hz, 4H), 7.74 (s, 4H), 1.44 (s, 18H); **^{13}C NMR** (101 MHz, $CDCl_3$) δ 192.8, 153.1, 141.5, 140.0, 137.1, 130.6,

128.0, 126.0, 125.9, 35.2, 31.5; **MS *m/z*** (ESI+): 421.2 ($C_{28}H_{30}NaO_2$, $[M+Na]^+$ requires 421.21); **AT-IR** (neat, cm^{-1}): 2963, 1698, 1596, 1176.

*Bisaldehyde **6***

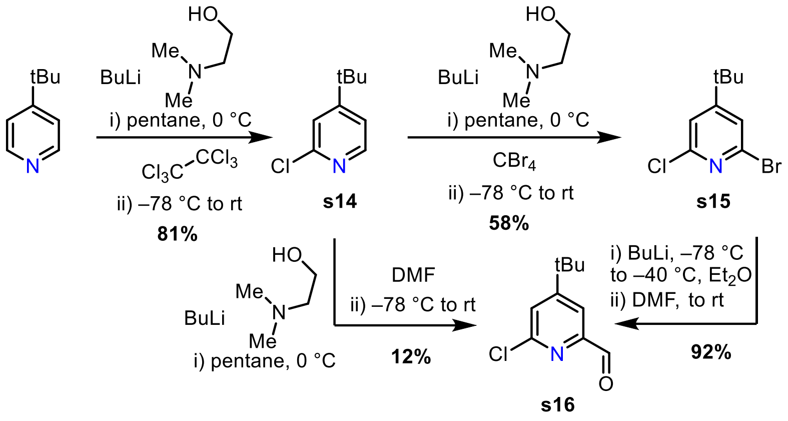

Figure S2. *Synthesis of choloropyridine **s16***

4-(tert-butyl)-2-chloropyridine s14: Using an adapted literature procedure,(Kaminski et al., 2003) in a flame-dried flask under argon, dry 2-(dimethylamino)ethan-1-ol (1.43 g, 1.61 mL, 2 eq, 16.0 mmol) was dissolved in pentane (50 mL), cooled to 0 °C, and added dropwise was butyllithium (1.6 M in hexanes) (2.050 g, 20.00 mL, 1.6 molar, 4 eq, 32.00 mmol) over 5 min. The reaction was stirred at 0 °C for 15 min and then added was a solution of 4-(tert-butyl)pyridine (1.08 g, 1.17 mL, 1 eq, 8.00 mmol) in pentane (10 mL), dropwise over 2 min. After stirring for 1 hour at 0 °C, the reaction was cooled to –78 °C and added was perchloroethane (4.734 g, 2.5 eq, 20.00 mmol) as a solution in THF (20 mL) and the reaction stirred for 1 hour at –78 °C. The reaction was allowed to warm to 0 °C over 10 min and then quenched with water (30 mL), extracted with diethyl ether (x2) and dried over magnesium sulfate. After filtration, the reaction was concentrated to a residue, the solid insoluble in petrol discarded, and the liquid purified by flash column chromatography (biotage, 25 g, loaded in petrol, 0-5% EtOAc/petrol). The desired product **s14** eluted as an orange oil (1.10 g, 81%) with data consistent with the literature:[11] ^{1}**H NMR** (400 MHz, $CDCl_3$) δ 8.31 – 8.26 (m, 1H), 7.31 – 7.28 (m, 1H), 7.20 (dd, *J* = 5.3, 1.7 Hz, 1H), 1.31 (s, 9H); ^{13}**C NMR** (101 MHz, $CDCl_3$) δ 163.7, 151.9, 149.5, 121.4, 119.8, 35.1, 30.5; **MS** ESI(+): 170.0, 172.0.

Conversion of s14 to 2-bromopyridine s15 and subsequent conversion to 2-carboxaldehyde-pyridine s16

2-bromo-4-(tert-butyl)-6-chloropyridine s15: Using an adapted literature procedure,(Kaminski et al., 2003) in a flame-dried flask under argon, dry 2-(dimethylamino)ethan-1-ol (2.67 g, 3.01 mL, 2 eq, 29.9 mmol) in pentane (90 mL) was cooled to 0 °C and added dropwise was butyllithium (1.6 M in hexanes) (3.84 g, 37.4 mL, 1.6 molar, 4 eq, 59.9 mmol) over 5 min. The reaction was stirred at 15 min for 0 °C and then added was a solution of 4-(tert-butyl)-2-chloropyridine (2.54 g, 1 eq, 15.0 mmol) in pentane (36 mL), dropwise over 2 min. After stirring for 0 °C at 1 hour, the reaction was cooled to –78 °C and added was carbon tetrabromide (12.4 g, 2.5 eq, 37.4 mmol) as a solution in THF (36 mL) and the reaction stirred for 1 hour. The reaction was allowed to warm to 0 °C over 10 min and then quenched with water (60 mL), extracted with diethyl ether (x2) and dried over magnesium sulfate. After filtration, the reaction was concentrated to a residue, the solid insoluble in petrol/dichloromethane (1:1) discarded, and the liquid purified by flash column chromatography (biotage, 25 g, loaded in petrol/dichloromethane, 0-50% EtOAc/petrol). The desired product **s15** eluted as a brown solid (2.16 g,

58%) with data: **^{1}H NMR (400 MHz, $CDCl_3$)** δ 7.38 (d, *J* = 1.4 Hz, 1H), 7.26 (d, *J* = 1.4 Hz, 1H), 1.30 (s, 9H); **^{13}C NMR (101 MHz, $CDCl_3$)** δ 166.1, 150.8, 140.9, 124.2, 120.7, 35.5, 30.5; **HR-MS m/z** (ESI+): 247.9847 ($C_9H_{12}BrClN$, MH+ requires 247.9836).

4-(tert-butyl)-6-chloropicolinaldehyde s16: Using an adapted literature procedure,(Kaminski et al., 2003) in a flame-dried flask under argon, 2-bromo-4-(tert-butyl)-6-chloropyridine (1.50 g, 1 Eq, 6.04 mmol) was dissolved in diethyl ether (20 mL) and cooled to –78 °C. Butyllithium (1.6 M in hexanes) (425 mg, 4.15 mL, 1.6 molar, 1.1 eq, 6.64 mmol) was added dropwise (the solution became dark immediately), and the was reaction stirred at –78 °C for 30 min. After this time, the reaction was warmed to –30 °C and added was N,N-dimethylformamide (662 mg, 701 µL, 1.5 Eq, 9.05 mmol), maintaining –30 °C. The reaction was warmed to 0 °C over 10 min. The reaction was quenched with saturated aqueous ammonium chloride solution and extracted with diethyl ether, washed with brine x 4, dried over magnesium sulfate, filtered and concentrated under reduced pressure. The residue was purified by flash column chromatography to give 4-(tert-butyl)-6-chloropicolinaldehyde **s16** (1.1 g, 5.6 mmol, 92 %) as a pale yellow oil. **Data matches s16, below.**

Direct reaction of s14 to s16

4-(tert-butyl)-6-chloropicolinaldehyde s16: Using an adapted literature procedure,(Kaminski et al., 2003) in a flame-dried flask under argon, dry 2-(dimethylamino)ethan-1-ol (946 mg, 1.07 mL, 2 eq, 10.6 mmol) in pentane (30 mL) was cooled to 0 °C and added dropwise was butyllithium (1.6 M in hexanes) (1.359 g, 13.26 mL, 1.6 molar, 4 eq, 21.22 mmol) over 5 min. The reaction was stirred at 15 min for 0 °C and then added was a solution of 4-(tert-butyl)-2-chloropyridine **s14** (0.900 g, 1 eq, 5.31 mmol) in pentane (12 mL), dropwise over 2 min. After stirring for 0 °C at 1 hour, the reaction was cooled to –78 °C and added was N,N-dimethylformamide (970 mg, 1.03 mL, 2.5 eq, 13.3 mmol) as a solution in THF (12 mL) and the reaction stirred for 1 hour. The reaction was allowed to warm to 0 °C over 10 min and then quenched with water (20 mL), extracted with diethyl ether (x2) and dried over magnesium sulfate. After filtration, the solution was concentrated to a residue, and purified by flash column chromatography, (biotage, 25 g, loaded in petrol/dichloromethane, 0-5% EtOAc/petrol). The product was not clean, and was repurified by chromatography (biotage, 25 g, loaded in petrol/dichloromethane, 0-50% CH_2Cl_2/petrol) to give the desired product **s16** as a pale yellow oil (126 mg, 12%): **^{1}H NMR (400 MHz, $CDCl_3$)** δ 9.98 (s, 1H), 7.88 (d, *J* = 1.6 Hz, 1H), 7.52 (d, *J* = 1.6 Hz, 1H), 1.34 (s, 9H); **^{13}C NMR (101 MHz, $CDCl_3$)** δ 192.3, 165.2, 152.9, 152.4, 126.0, 117.7, 35.6, 30.5; **HR-MS m/z** (ESI+): 198.0681 ($C_{10}H_{13}NCl$, MH^+ requires 198.0680).

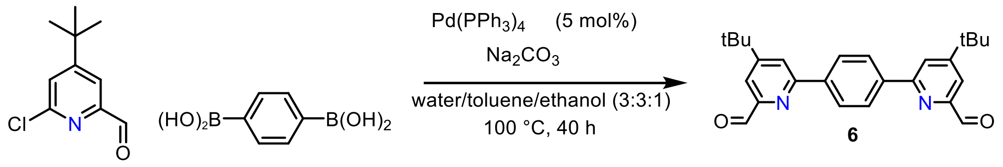

6,6'-(1,4-phenylene)bis(4-(tert-butyl)picolinaldehyde) 6: In a flame-dried flask under argon, 4-(tert-butyl)-6-chloropicolinaldehyde (477.0 mg, 2 Eq, 2.413 mmol), and tetrakis(triphenylphosphine)palladium(0) (86.0 mg, 0.0617 eq, 74.4 µmol) were dissolved in toluene (9 mL) and ethanol (3 mL) and the mixture briefly degassed (three vacuum/argon cycles). Added was a solution of sodium carbonate (447.6 mg, 3.5 eq, 4.223 mmol) in water (9 mL), and the reaction degassed once more. The mixture was then stirred vigorously at 100 °C for 40 hour. The reaction mixture was cooled, diluted with ethyl acetate, and washed with sodium hydroxide and brine and dried over magnesium sulfate, filtered and concentrated. The residue was purified by flash column chromatography, eluting with 0-20% EtOAc/petrol (holding at 14%) to give **6** as a pale yellow solid (236 mg, 49%); **^{1}H NMR (400 MHz, $CDCl_3$)** δ 10.21 (s, 2H), 8.24 (s, 4H), 8.01 (d, *J* = 1.7 Hz, 2H), 7.97 (d, *J*

= 1.7 Hz, 2H), 1.44 (s, 18H); **^{13}C NMR (101 MHz, CDCl$_3$)** δ 194.5, 162.6, 157.5, 153.1, 139.8, 127.7, 121.9, 117.6, 35.5, 30.7; **HR-MS m/z** (ESI+): 401.2222 ($C_{26}H_{29}O_2N_2$, MH$^+$ requires 401.2224).

*Bisaldehyde **7***

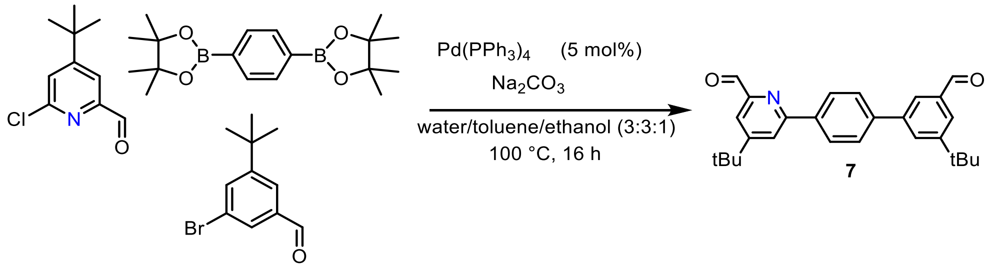

6,6'-(1,4-phenylene)bis(4-(tert-butyl)picolinaldehyde) 7: In a flame-dried flask under argon, 4-(tert-butyl)-6-chloropicolinaldehyde (245.9 mg, 1.244 mmol), 3-bromo-5-(tert-butyl)benzaldehyde (300.0 mg, 1.244 mmol), and tetrakis(triphenylphosphine)palladium(0) (100.6 mg, 87.09 µmol) were dissolved in toluene (30 mL) and ethanol (10 mL) and the mixture briefly degassed (three vacuum/argon cycles). Added was a solution of sodium carbonate (791.2 mg, 3.5 eq, 7.465 mmol) in water (30 mL), and the reaction degassed once more. The mixture was then stirred vigorously at 100 °C for 16 h. The reaction mixture was cooled, diluted with ethyl acetate, and washed with sodium hydroxide and brine and dried over magnesium sulfate, filtered and concentrated. The residue was purified by flash column chromatography, eluting with 0-25% EtOAc/petrol to obtain a mixture of the three bisaldehyde products. This mixture was resubjected to chromatography with 0-100% CH_2Cl_2/petrol, to give the desired mixed bisaldehyde **7** as a pale yellow solid eluting at 90-95% CH_2Cl_2 (83.4 mg, 17%); **^{1}H NMR** (400 MHz, CDCl3) δ 10.20 (s, 1H), 10.12 (s, 1H), 8.19 (d, *J* = 8.5 Hz, 2H), 8.00 – 7.93 (m, 8H), 7.78 (d, *J* = 8.5 Hz, 2H), 1.45 (s, 11H), 1.43 (s, 11H); **^{13}C NMR** (101 MHz, CDCl3) δ 194.4, 192.7, 162.4, 157.5, 153.0 (d, *J* = 1.8 Hz), 141.3, 138.3, 137.0, 130.4, 127.8, 126.0, 125.9, 121.7, 117.3, 35.4, 35.1, 31.4, 30.6; **HR-MS m/z** (ESI+): 400.2289 ($C_{27}H_{30}O_2N$, MH$^+$ requires 400.2271).

Synthesis of cages **1**, **2e** and **3e**

Synthesis of cage **1**

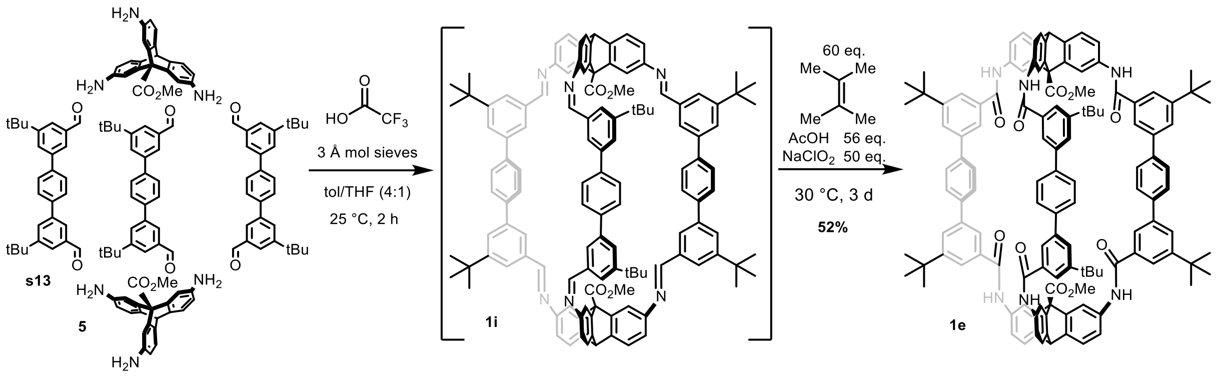

Imine cage 1i: As previously reported,[10] in a 5 L flask, triptycene **5** (1.50 g, 4.20 mmol) was dissolved in THF (750 mL) and the solution diluted with toluene (1500 mL) containing trifluoroacetic acid (150 µL, 1.96 mmol). Separately, bisaldehyde **s13** (2.51 g, 6.30 mmol) was dissolved in (regular) toluene (1500 mL) and the solution added over 60 seconds. After stirring for 2 h at ambient temperature, analytical GPC and MALDI-ToF analysis indicated high conversion to the desired hexaimine cage. Added was 2,3-dimethyl-2-butene (15.0 mL, 126 mmol, 60 eq), sodium chlorite (9.49 g, 105 mmol, 50 eq) and glacial acetic acid (6.73 mL, 118 mmol, 56 eq.). The reaction was stirred vigorously in the dark for 3 d. The reaction was filtered, quenched with aqueous sodium thiosulfate, and extracted three times with ethyl acetate. The organics were washed with aqueous sodium hydroxide (0.5 M) and brine, and the resulting organics purified in five batches by recycling gel-permeation chromatography (THF, 5 x 90 min cycles). The resulting tan solid was triturated with petrol/diethyl ether to remove impurities from the THF (gamma-butyrolactone, butylated-hydroxytoluene (BHT)) to give an off-white solid (2.09 g, 52% over two steps).

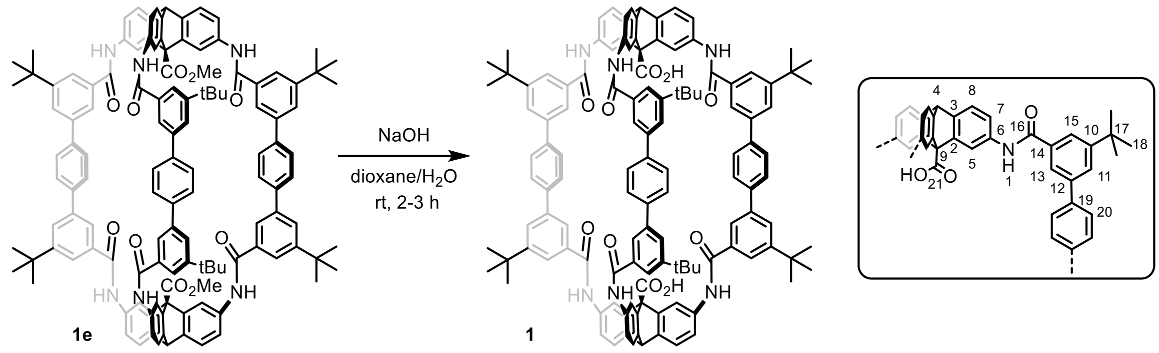

Diacid cage 1: Dimethyl ester cage **1e** (2.09 g, 1.10 mmol) was dissolved in dioxane (120 mL) and added was an aqueous sodium hydroxide solution (3.3 g, 41.3 mL, 2 M, 75 Eq, 82.6 mmol). The reaction was followed by TLC until complete (~1-2 h) and then cautiously quenched with dilute HCl until acidified. The mixture was extracted with ethyl acetate, and the organics washed with water and dried over magnesium sulfate, filtered and concentrated under reduced pressure. The resulting solid was triturated in pentane, and the desired cage **1** was collected by filtration and vacuum dried at 55 °C, 0.3 mbar (2.06 g, quant.); **^{1}H NMR** (500 MHz, THF-d$_8$) δ 9.57 (s, 6H^{1}), 8.44 (d, *J* = 1.7 Hz, 6H^{5}), 8.23 (dd, *J* = 1.5, 1.5 Hz, 6H^{13}), 8.12 (dd, *J* = 1.5, 1.5 Hz, 6H^{15}), 7.86 (dd, *J* = 1.5, 1.5 Hz, 6H^{11}), 7.85 (s, 12H^{20}), 7.83 (dd, *J* = 8.2, 1.8 Hz, 6H^{7}), 7.41 (d, *J* = 8.1 Hz, 6H^{8}), 5.46 (s, 2H^{4}), 1.43 (s, 54H^{18}); **^{13}C NMR** (126 MHz, THF-d$_8$) δ 172.0 (C^{21}), 165.6 (C^{16}), 152.9 (C^{10}), 145.1 (C^{3}), 142.8 (C^{2}), 141.5 (C^{12,19}), 137.6 (C^{6}), 136.7 (C^{14}),

128.6 (C[20]), 127.8 (C[11]), 125.1 (C[15]), 124.3 (C[13]), 124.0 (C[8]), 118.2 (C[7]), 117.9 (C[5]), 63.3 (C[9]), 54.0 (C[4]), 35.9 (C[17]), 31.8 (C[18]); **AT-IR** (neat, cm^{-1}): 2959, 1738, 1660, 1596, 1519, 1469.

Synthesis of cage **2e**

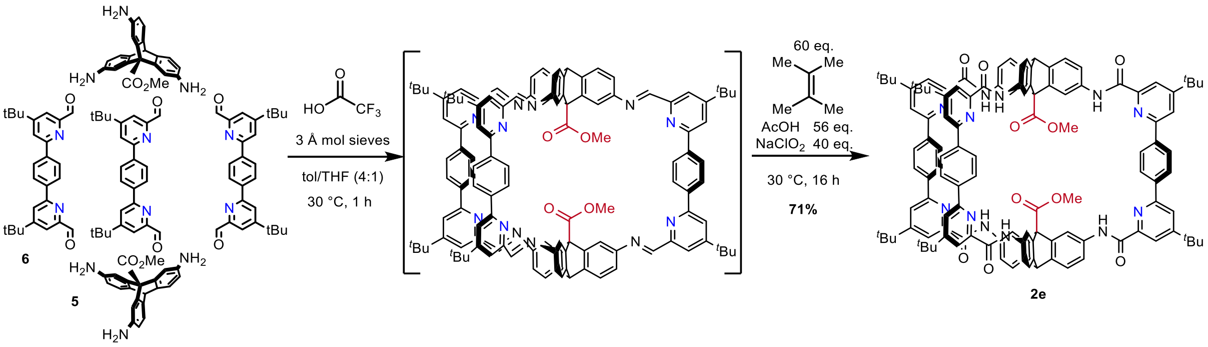

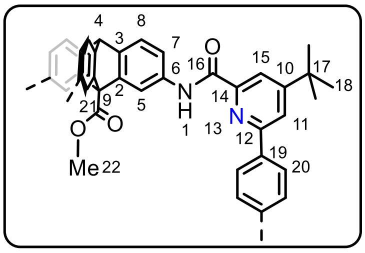

Dimethylester amide cage 2: Triptycene **5** (100 mg, 280 µmol) was dissolved in THF (50 mL) in a flask containing 3 Å molecular sieves (0.15 g). The solution was diluted with toluene (100 mL) containing trifluoroacetic acid (10 µL, 131 µmol). Separately, bisaldehyde **6** (168 mg, 420 µmol) was dissolved in (regular) toluene (100 mL) and this solution added to the reaction over 60 seconds. After stirring for 1 h at ambient temperature, MALDI-ToF analysis indicated high conversion to the desired hexaimine cage. Added was 2,3-dimethyl-2-butene (998 µL, 8.39 mmol, 60 eq), sodium chlorite (80% w/w, 633 mg, 5.60 mmol, 40 eq) and glacial acetic acid (448 µL, 7.83 mmol, 56 eq). The reaction was stirred vigorously in the dark at 30 °C for 16 h. The reaction was filtered to remove the solids, diluted with ethyl acetate (200 mL), and washed with water and brine, and the resulting organics dried over magnesium sulfate, filtered and concentrated. The resulting solid was triturated from THF to give **2e** as a white powder (189 mg, 71%) with data: **^{1}H NMR (600 MHz, CDCl$_3$)** δ 9.90 (s, 6H[1]), 8.32 (d, *J* = 1.7 Hz, 6H[11]), 8.06 (s, 12H[20]), 7.98 (dd, *J* = 8.1, 1.9 Hz, 6H[7]), 7.84 (d, *J* = 1.7 Hz, 6H[15]), 7.51 (d, *J* = 8.1 Hz, 6H[8]), 7.50 (d, *J* = 1.9 Hz, 6H[5]), 5.47 (s, 2H[4]), 4.62 (s, 6H[22]), 1.41 (s, 54H[18]); **^{13}C NMR (151 MHz, CDCl$_3$)** δ 170.6 (C[21]), 163.1 (C[10]), 162.8 (C[16]), 156.7 (C[12]), 150.0 (C[14]), 143.3 (C[3/2]), 142.2 (C[3/2]), 140.5 (C[6]), 134.3 (C[19]), 128.0 (C[20]), 124.3 (C[8]), 121.4 (C[15]), 120.0 (C[7]), 118.9 (C[11]), 117.0 (C[5]), 61.8 (C[9]), 53.2 (C[4]), 52.7 (C[22]), 35.5 (C[17]), 30.7 (C[18]); **MS *m/z*** (MALDI-ToF-RP) (DCTB matrix) 1903.5 ($C_{122}H_{111}N_{12}O_{10}$, $[M+H]^+$ requires 1903.9).

The oxidation step can also be performed with NaH_2PO_4 (56 eq) instead of AcOH (56 eq) as the acid, but the reaction tends to take ~10 days.

Synthesis of cage 3e

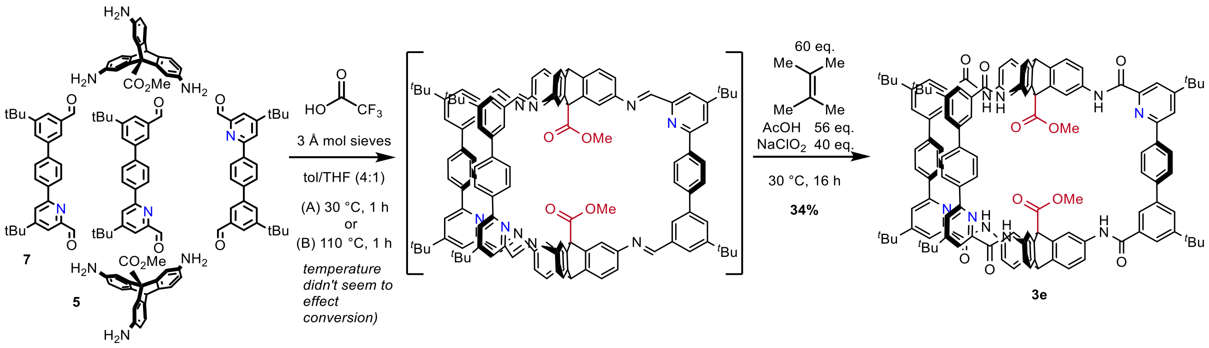

Bisester Amide cage 3e: Reaction (A) Triptycene **5** (20 mg, 56.0 µmol) was dissolved in THF (10 mL) in a flask containing 3 Å molecular sieves (0.20 g). The solution was diluted with toluene (20 mL) containing trifluoroacetic acid (2 µL, 26.2 µmol). Separately, bisaldehyde **7** (33.5 mg, 83.9 µmol) was dissolved in (regular) toluene (20 mL) and this solution added to the reaction over 60 seconds. After stirring for 1 h at ambient temperature, MALDI-ToF analysis indicated high conversion to the desired hexaimine cage.

Reaction (B): The same process was separately completed stirring at 110 °C for 1 h. MALDI-ToF analysis showed no difference from the first procedure.

Reaction A and B, separately: Added was 2,3-dimethyl-2-butene (200 µL, 1.68 mmol, 60 eq), sodium chlorite (80% w/w, 127 mg, 1.40 mmol, 50 eq) and glacial acetic acid (89.7 µL, 1.57 mmol, 56 eq). The reaction was stirred vigorously in the dark at 30 °C for 16 h. MALDI-ToF indicated full conversion to the hexamide cage. The reaction was filtered to remove the solids, diluted with ethyl acetate (100 mL), and washed with water and brine, and the resulting organics dried over magnesium sulfate, filtered and concentrated. ^{1}H-NMR analysis of the crude material indicated reactions A and B had no difference in conversion. The combined crudes from reactions A and B were dissolved in THF and purified by purified by recycling gel-permeation chromatography (THF, 6 x 90 min cycles). The resulting solid was triturated with diethyl ether (to remove impurities from the THF) to give an off-white solid **3e** (17.8 mg, 34% over two steps) with data which could be partially (but definitively) assigned using 2D methods: **^{1}H NMR (600 MHz, $CDCl_3$)** δ 10.27 (s, 1H$^{1a'}$), 9.93 (s, 2H^{1a}), 8.63 – 8.55 (m, 1H$^{5b'}$), 8.39 (dd, *J* = 8.0, 1.8 Hz, 1H$^{7a'}$), 8.31 (d, *J* = 1.7 Hz, 2H^{11a}), 8.28 (d, *J* = 1.7 Hz, 1H$^{11a'}$), 8.24– 8.19 (m, 4H^{7a,5b}), 8.14 (d, *J* = 8.2 Hz, 2H$^{20a'}$), 8.08 (d, *J* = 8.2 Hz, 4H^{20a}), 8.01 (dd, *J* = 1.4, 1.4 Hz, 2H^{13b}), 7.90 (d, *J* = 1.8 Hz, 1H$^{15a'}$), 7.89 – 7.88 (m, 1H$^{15b'/11b'}$), 7.88 – 7.86 (m, 1H$^{15b'/11b'}$), 7.85 (d, *J* = 1.8 Hz, 2H^{15a}), 7.84 – 7.76 (m, 11H$^{15b/11b/20b/20b'/13b'}$), 7.73 (s, 1H$^{5a'}$), 7.60 (s, 2H^{5a}), 7.51-7.45 (m, 7b, 2H^{7b}), 7.50 (d, *J* = 8.3 Hz, 2H$^{8a/8a'/8b/8b'}$), 7.49 (d, *J* = 8.2 Hz, 1H$^{8a/8a'/8b/8b'}$), 7.44 (d, *J* = 7.8 Hz, 3H$^{8a/8a'/8b/8b'}$), 7.19 – 7.10 (m, 1H$^{7b'}$), 5.44 (s, 1H$^{4/4'}$), 5.43 (s, 1H$^{4/4'}$), 4.60 – 4.51 (m, 6H$^{22,22'}$), 1.43 (s, 18H^{18b}), 1.43 (s, 9H$^{18b'}$), 1.42 (s, 9H$^{18a'}$), 1.41 (s, 18H^{18a}). (*Note: some ^{1}H-signals move depending on chloroform acidity; amide NH groups only visible when adjacent to pyridine);* **^{13}C NMR (151 MHz, $CDCl_3$)** δ 170.58 (C$^{21,21'}$), 165.72 (C$^{16b/16b'}$), 165.15 (C$^{16b/16b'}$), 163.28 (C$^{10a',16a'}$), 163.03 (C^{10a,16a}), 162.97 (C^{10a,16a}), 162.50 (C$^{10a',16a'}$), 156.43 (C^{12a}), 155.78 (C$^{12a'}$), 152.66 (C^{10b}), 152.41 (C$^{10b'}$), 150.02 (C^{14a}), 149.64 (C$^{14a'}$), 144.20 (C$^{2/3}$), 143.71 (C$^{2/3}$), 143.36 (2C$^{2/3}$), {142.19, 141.98, 141.96, 141.79, 141.68, 141.49, 141.37, 141.19 (C$^{2b,2b',3b,3b',6a,6a',19b,19b'}$)}, 138.70 (C^{14b}), 138.24 (C$^{14b'}$), 135.79 (C$^{6b/6b'/12b/12b'/19a/19a'}$), 135.50 (C$^{6b/12b/19a}$), 135.44 (C$^{6b'/12b'/19a'}$), 135.24 (C$^{6b/6b'/12b/12b'/19a/19a'}$), 135.13 (C$^{6b/6b'/12b/12b'/19a/19a'}$), 134.77 (C$^{6b/12b/19a}$), 128.21 (C$^{20a/20b}$), 128.07 (C$^{11b'}$), 128.04 (C$^{20a'/20b'}$), 128.00 (C^{11b}), 127.96 (C$^{20a/20b}$), 127.73 (C$^{20a'/20b'}$), 124.33 (C$^{13b'/8}$), 124.28 (C$^{13b'/8}$), 124.16 (C^{8}), 123.78 (C^{13b}), 123.75 (C^{8}), 123.69 (C^{8}), 123.25 (C^{15b}), 122.37 (C$^{15b'}$), 121.06 (C^{15a}), 120.49 (C$^{15a'}$), 118.72 (C^{11a,7a}), 118.31 (C$^{11a'}$), 117.54 (C^{7b}), 117.31 (C$^{7b'}$), 116.81 (C^{5a}), 116.69 (2C^{5b}), 116.65 (C$^{7a'}$), 116.44 (C$^{5b'}$), 115.78 (C$^{5a'}$); 62.14 (C^{9}), 61.95 (C^{9}), 53.20 (C$^{4/4'}$), 52.91 (C$^{4/4'}$), 52.87 (C$^{4/4'}$), 35.47 (C$^{17a'}$), 35.45 (C^{17a}), 35.21 (C^{17b}), 35.20 (C$^{17b'}$), 31.51 (C$^{18b'}$), 31.47 (C^{18b}), 30.67 (C^{18a}), 30.65 (C$^{18a'}$). [a/b = either a or b; a,b = a and b overlapped]; **MS *m/z*** (MALDI-ToF-RP) (DCTB matrix) 1922.3 ($C_{125}H_{113}N_9O_{10}Na$, $[M+Na]^+$ requires 1922.8).

Analysis of cage **3e**

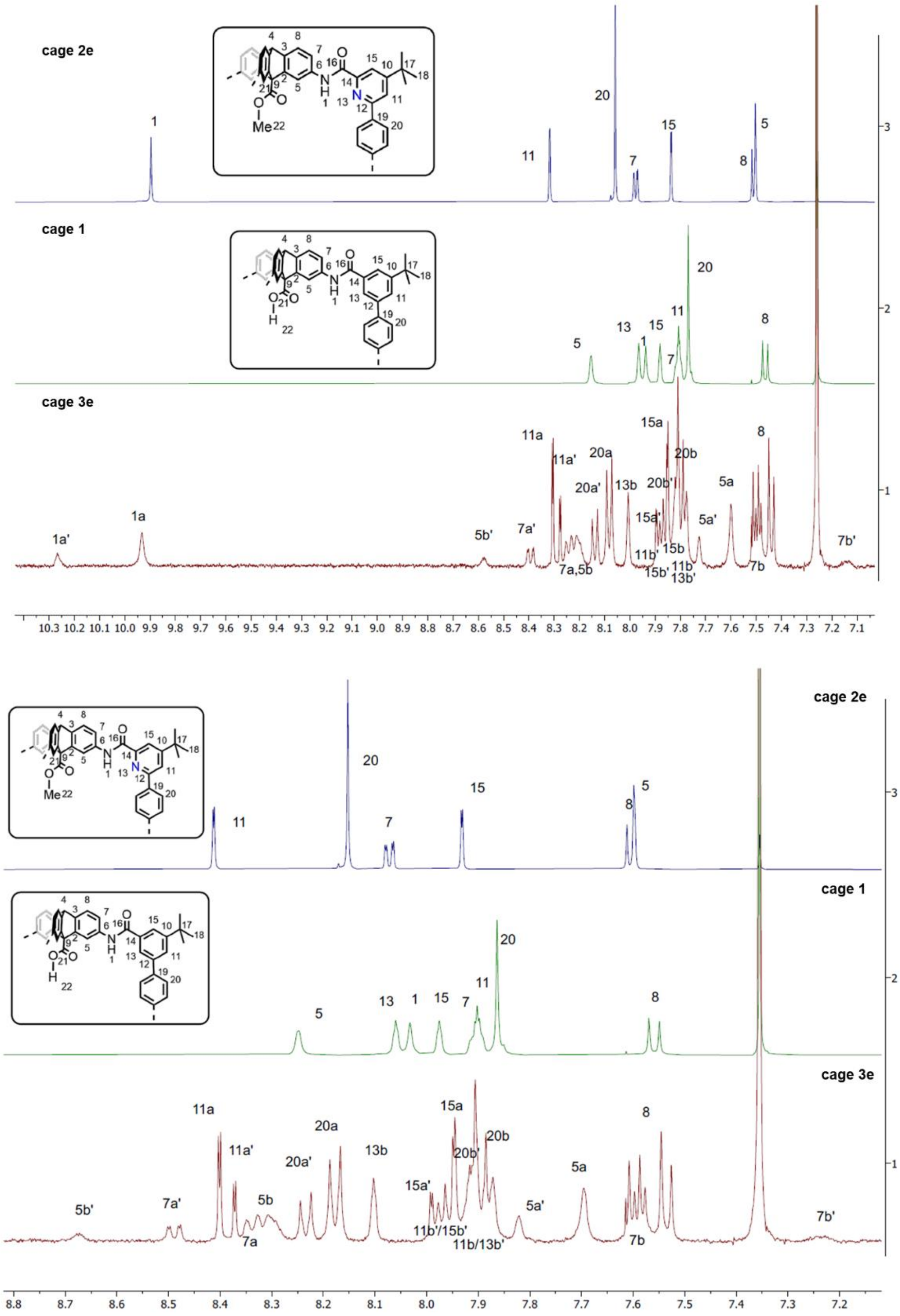

Figure S3. ^{1}H-NMR spectra ($CDCl_3$) of cage **3e** (compared to cages **2e** and **1**), showing expected 2:1 ratio of signals consistent with the **UUD** configuration and the **C5** conformation. (bottom, slight zoom in of top).

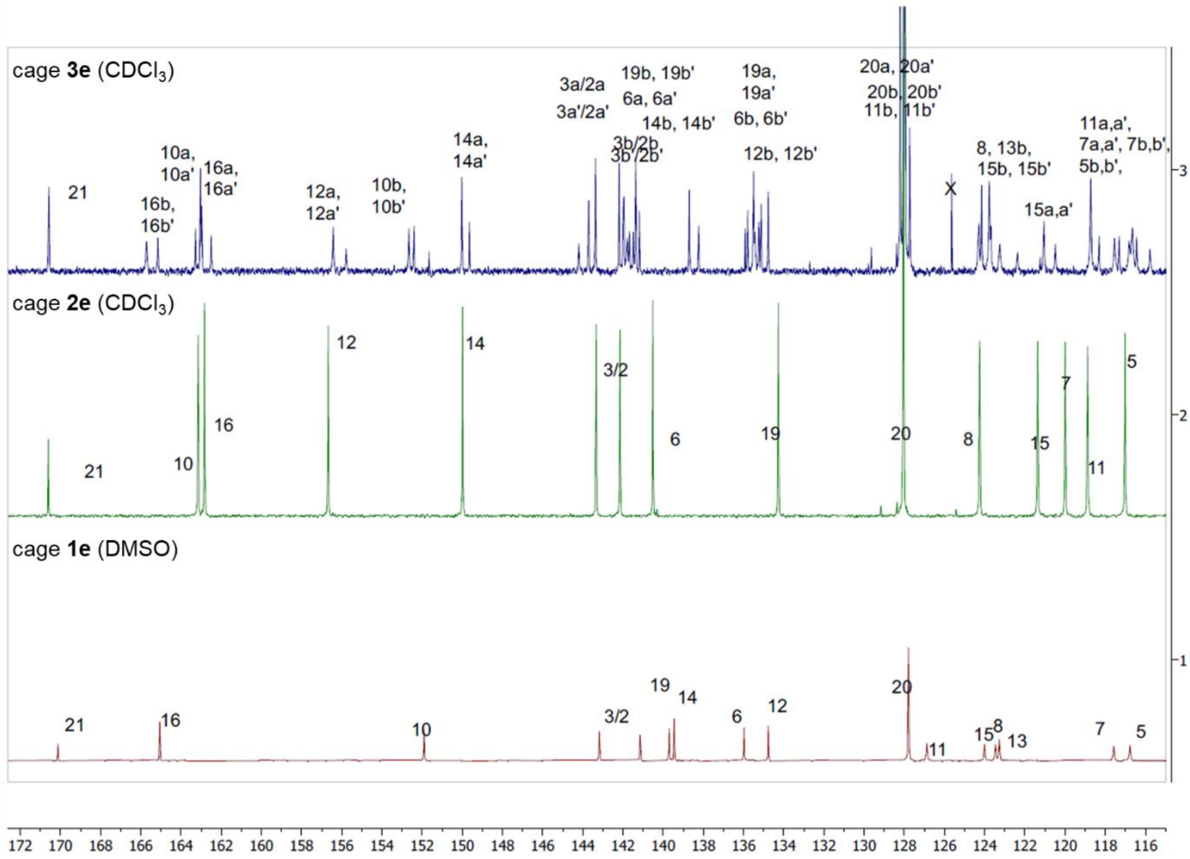

Figure S4. ^{13}C-NMR spectra of cage **3e** (compared to cages **2e** and **1**).

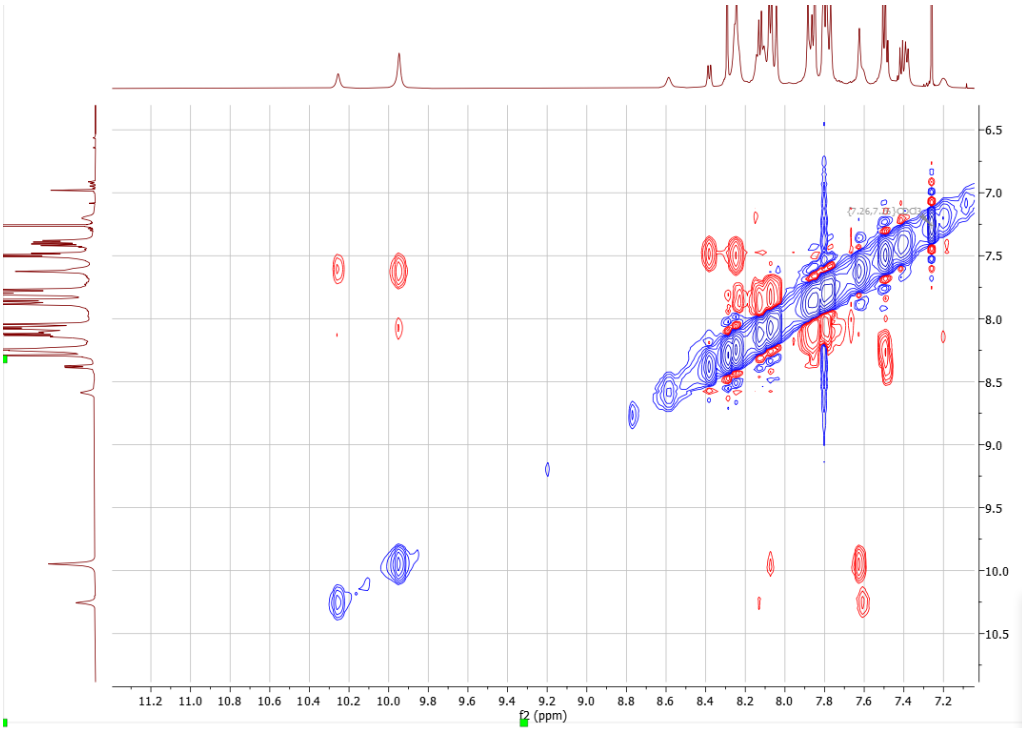

Figure S5. *2D-EXSY experiments* suggest no rotation / interconversion of environments by the pyridine groups (the pyridyl amide NH groups at ~10 ppm are not in exchange).

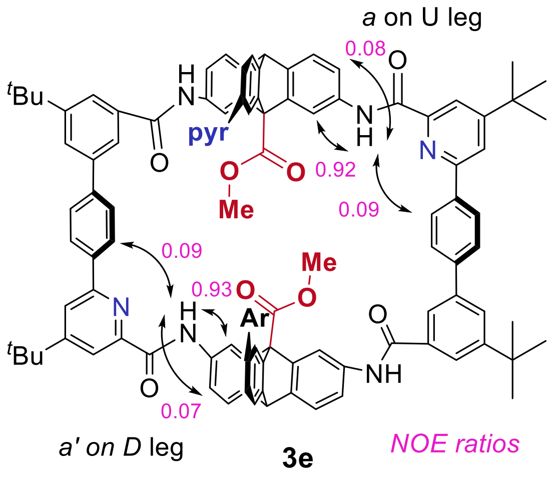

Figure S6. *Cage **3e** NOE analysis for amide NH environments a (U leg) and a' (D leg). The values strongly indicate "carbonyl out" amide orientations. See also Figures S28-33 for NOE values compared to other cages. ^{1}H-NMR ($CDCl_3$) 298 K.*

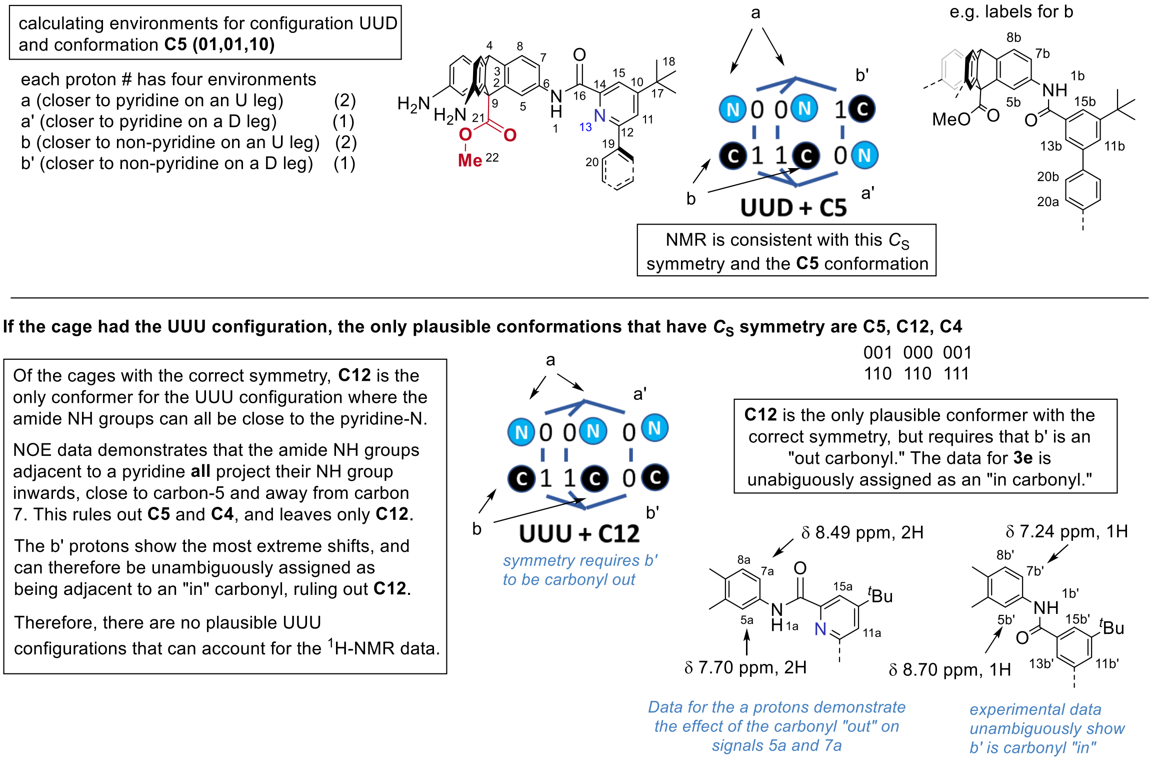

Figure S7. *NMR analysis demonstrates that the UUD configuration and C5 conformer are the only plausible candidates for the identity and major conformation of cage **3e**. There are no conformations of the UUU configuration of the cage that have the required symmetry to match the environment ratios for cage **3e** that have the b' protons assigned as being adjacent to an "in carbonyl", which is unambiguously evident from the chemical shifts.*

Synthesis of the anthracene bisaldehyde **8**

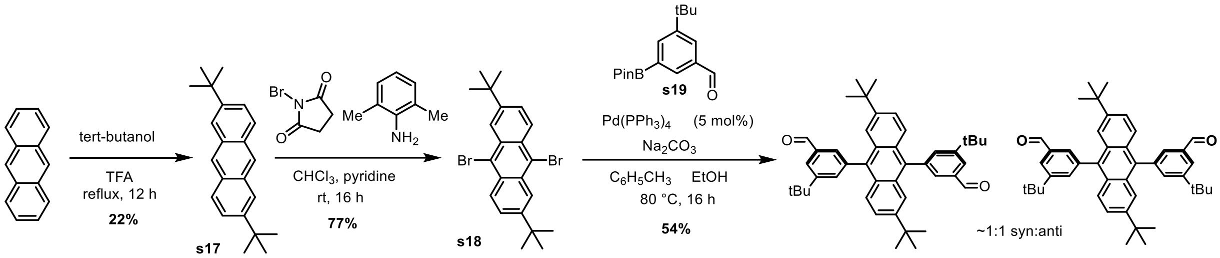

8 *(mixture of atropisomers)*

2,6-di-tert-butylanthracene s17: According to an adapted literature procedure,[12] anthracene (4.00 g, 22.4 mmol) was suspended in trifluoroacetic acid (22.4 mL) and added was *t*-butanol as a solid (5.94 g, 80.1 mmol). The reaction was stirred at reflux (80 °C) under a nitrogen balloon atmosphere for 16 h. After cooling, the greenish grey solid was filtered, and dissolved in petrol (with a small amount of dichloromethane and THF to aid solubility) (150 mL total). The solution was washed with saturated aqueous sodium bicarbonate solution, dried over magnesium sulfate, filtered and concentrated. The solid was crystallised from hot ethyl acetate and hexane to give a white solid (94% purity) which was recrystallised in boiling hexanes using ethyl acetate as an anti-solvent to give 4 crops of **s17** (total: 1.47 g, 22%) with data consistent with the literature.[12] **^{1}H NMR** (400 MHz, $CDCl_3$) δ 8.32 (s, 2H), 7.93 (d, *J* = 8.9 Hz, 2H), 7.87 (d, *J* = 2.0 Hz, 2H), 7.55 (dd, *J* = 8.9, 2.0 Hz, 2H), 1.45 (s, 18H).

9,10-dibromo-2,6-di-tert-butylanthracene s18: 2,6-Di-tert-butylanthracene (800 mg, 4.49 mmol) was dissolved in dichloromethane (22 mL) and added was 2,6-dimethylaniline (5.5 µL, 1 mol%) as a catalyst. The solution was cooled to 0 °C and added was *N*-bromosuccicinimide (1.76 g, 9.87 mmol, 2.2 eq.) and the reaction stirred at rt for 16 h. The reaction was quenched with acetone (2 mL), concentrated, and the resulting mixture triturated with pentane/dichloromethane (3:1). The white solid (mostly succinimide by-product) was filtered off, and the trituration solution concentrated. The resulting residue was triturated with methanol to give a pale yellow solid (550 mg, 47%). The white succinimide by-product was found to contain some product, and trituration of this solid in methanol gave a clean sample of product as a yellow crystalline solid (340 mg, 30%). The overall yield of **s18** was 77%. The solid had data consistent with the literature:[12] **^{1}H NMR** (400 MHz, $CDCl_3$) δ 8.51 (dd, *J* = 9.2, 0.6 Hz, 2H), 8.46 (dd, *J* = 1.9, 0.6 Hz, 2H), 7.71 (dd, *J* = 9.2, 1.9 Hz, 2H), 1.49 (s, 18H).

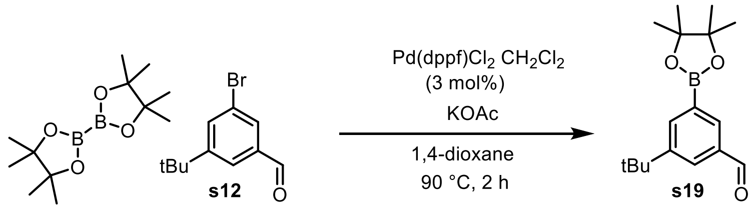

3-(tert-butyl)-5-(4,4,5,5-tetramethyl-1,3,2-dioxaborolan-2-yl)benzaldehyde (3-(tert-butyl)-5-(B-pin)benzaldehyde) s19: In a round-bottomed flask (100 mL) was combined 3-bromo-5-(tert-butyl)benzaldehyde (1.20 g, 4.98 mmol), bis(pinacolato)diboron (1.39 g, 5.47 mmol), [1,1′-bis(diphenylphosphino)ferrocene]dichloropalladium(II) complex with dichloromethane (122 mg, 0.149 mmol) and potassium acetate (1.47 g, 14.9 mmol) in 1,4-dioxane (20 mL). The solution was briefly degassed with three vacuum/argon cycles and then heated at 90 °C for 2 h. After this time, ^{1}H-NMR analysis suggested 93% desired product, with 7% of the presumed cross-coupled dimer. After cooling, the reaction was filtered through Celite, washing the filter bed with ether. The organic solution was washed with water and brine, dried over magnesium sulfate, filtered and concentrated. The residue was loaded onto a short silica pad and the desired eluted with dichloromethane/petrol (1:3). The resulting white solid (1.33 g, 93%) of **s19** was used as was: **^{1}H NMR** (400 MHz, $CDCl_3$) δ 10.04 (s, 1H), 8.12 (dd, 1H, *J* = 1.6, 1.1 Hz), 8.09 (dd, *J* = 2.2, 1.1 Hz, 1H), 8.01 (dd, *J* = 2.2, 1.6 Hz, 1H), 1.38 (s, 9H), 1.37 (s,

12H); **AT-IR** (neat, cm^{-1}): 2968, 1700, 1590, 1460, 1370, 1262, 1190, 1143; **HR-MS** ***m/z*** (ESI+): 288.2006, 289.1790 (100%) ($C_{17}H_{26}BO_3$, $[M+H]^+$ requires 288.2006, 289.1790).

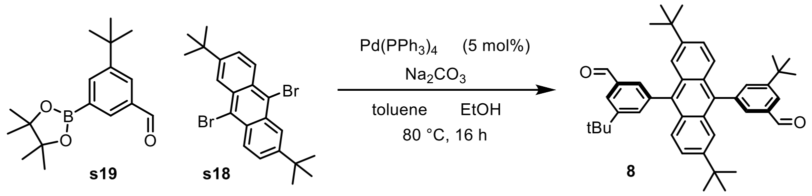

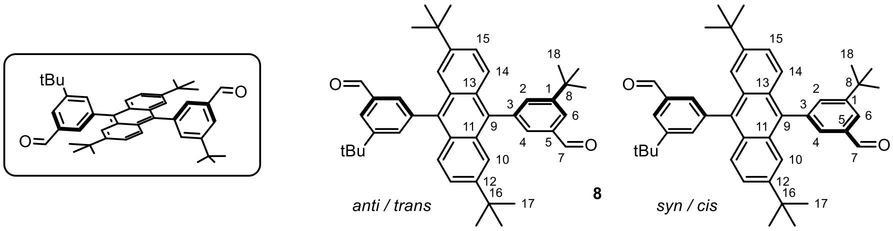

5,5'-(2,6-di-tert-butylanthracene-9,10-diyl)bis(3-(tert-butyl)benzaldehyde) (bisaldehyde 8): In a round-bottomed flask (50 mL) was combined boronic ester **s19** (500 mg, 1.73 mmol, 2.5 eq.), dibromoanthracene **s18** (311 mg, 0.694 mmol), and tetrakis(triphenylphosphine)palladium(0) (56.0 mg, 0.489 mmol) were combined in toluene (4.5 mL and ethanol (2.0 mL) in a flask fitted with a condenser under nitrogen. Added was an aqueous Na_2CO_3 solution (260 mg in 4.5 mL) and the reaction deaerated by three vacuum/argon cycles. The reaction was stirred at 85 °C for 16 h. The reaction was not complete, so a further portion of tetrakis(triphenylphosphine)palladium(0) (56.0 mg, 0.489 mmol) was added. After a further 16 h, the reaction had become black, was cooled, and diluted with ethyl acetate. The solution was filtered through Celite, and the organic solution washed with HCl(aq) (1 M), K_2CO_3(aq) (2 M), and brine, dried over magnesium sulfate, filtered, and concentrated. The residue was purified by flash column chromatography (Biotage, 25 g HP-SNAP cartridge, 0-5% EtOAc in petrol) to give **8** as a pale yellow powder (375 mg, 54%). The solid was an approximately 1:1 mixture of two magnetically inequivalent atropisomer environments, displaying two signals for all proton environments in chloroform, benzene, or DMSO. ^{13}C in benzene shows two distinct shifts for most carbon environments. At certain concentrations, the material reversibly gave an unexpected additional set of peaks, assumed to be due to aggregation of two molecules.

^{1}H NMR (400 MHz, $CDCl_3$) δ 10.18 (s, 2H), 10.17 (s, 2H), 8.14 (app q, *J* = 1.6 Hz, 4H), 7.87 (t, *J* = 1.5 Hz, 2H), 7.86 (p, *J* = 1.6 Hz, 4H), 7.83 (t, *J* = 1.8 Hz, 2H), 7.67 (dd, *J* = 1.5, 0.7 Hz, 2H), 7.65 (dd, *J* = 1.5, 0.7 Hz, 2H), 7.54 (d, *J* = 2.0 Hz, 4H), 7.52 – 7.49 (t, 2H), 7.49 – 7.47 (t, 2H), 1.48 (s, 18H), 1.47 (s, 18H), 1.28 (s, 36H); **^{1}H NMR** (400 MHz, DMSO-d_6) δ 10.18 (s, 2H), 10.18 (s, 2H), 8.16 (dd, *J* = 1.5, 1.5 Hz, 4H), 7.89 (t, *J* = 1.7 Hz, 2H), 7.87 (t, *J* = 1.5 Hz, 2H), 7.81 (dt, *J* = 2.8, 1.6 Hz, 4H), 7.64 – 7.55 (m, 8H), 7.48 – 7.44 (m, 4H), 1.42 (d, *J* = 3.6 Hz, 36H), 1.20 (s, 36H); **^{1}H NMR** (500 MHz, C_6D_6**)** δ 9.85 (s, 2H), 9.81 (s, 2H), 8.18 (dt, *J* = 9.4, 1.7 Hz, 4H), 7.90 – 7.84 (m, 15H), 7.83 (t, *J* = 1.5 Hz, 2H), 7.37 (dt, *J* = 9.2, 2.3 Hz, 4H), 1.23 (s, 18H, H^{18}), 1.22 (s, 18H, H^{18}), 1.21 (s, 18H, H^{17}), 1.21 (s, 18H, H^{17}); **^{13}C NMR** (126 MHz, C_6D_6) δ 191.8, 191.7, 152.9, 152.8, 147.9, 147.9, 140.5, 137.6, 137.6, 136.3, 136.3, 135.0, 135.0, 131.5, 131.3, 130.4, 130.4, 129.4, 129.4, 127.0, 127.0, 125.5, 125.5, 124.8, 124.6, 121.3, 35.1, 35.0, 35.0, 31.2, 31.2, 30.8; **HR-MS** ***m/z*** (ESI+): 611.3881 ($C_{44}H_{51}O_2$, M^+ requires 611.3884).

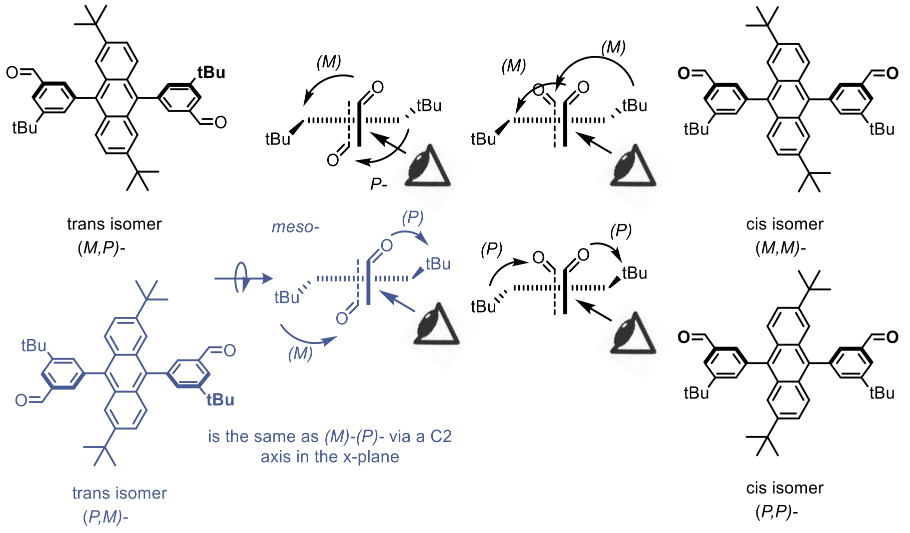

Figure S8. *Depiction of the atropisomers of anthracenyl bisaldehyde **8**.* Assignment of atropsiomers discussed here.[13]

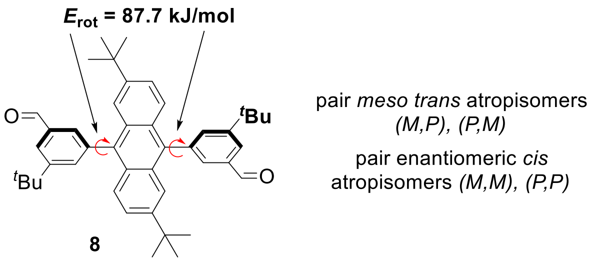

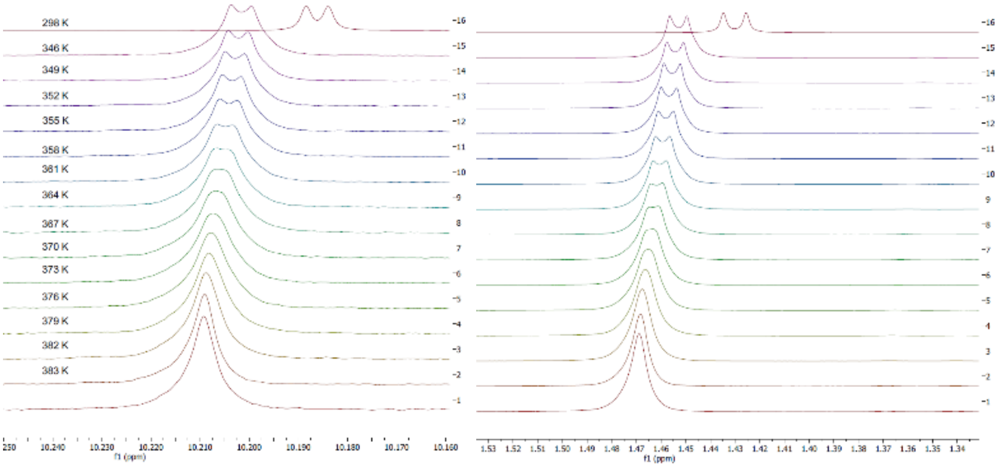

Figure S9. *Variable temperature (25-112 °C) ^{1}H-NMR spectra (DMSO-d6) of the anthracene bisaldehyde **8**.* ***RIGHT:*** *Aldehyde CH coalescence (367 K).* ***LEFT:*** *^{t}Bu CH_3 coalescence (376 K).*

The free energy barrier to rotation was estimated[14] using equation se1:

$$\frac{\Delta G^{\ddagger}}{RT_c} = 22.96 + \ln\left(\frac{T_c}{\delta_v}\right) \qquad \text{(eq. se1)}$$

Where T_c is the correlation temperature (K), R is the ideal gas constant (8.3145 J/mol/K), and δ_v is the low temperature chemical shift difference in Hz.

Table S1. *Calculation of rotational barrier of bisaldehyde* ***8***

**Signal**	δ_v **(Hz)**	T_c **/ K**	**RHS**	**Δ*G* (J/mol)**	**Δ*G* (kJ/mol)**
aldehyde	2.30	367	28.0	85539	85.5
^{t}Bu	4.62	376	27.4	85532	85.5
				average ΔG =	85.5

The rate (frequency) term was divided by two, because there are two rotatable bonds contributing to the overall measured rate. This gave slightly higher values for *ΔG.*

**Signal**	δ_v **(Hz)**	T_c **/ K**	**RHS**	**Δ*G* (J/mol)**	**Δ*G* (kJ/mol)**
aldehyde	1.15	367	28.7	87654	87.7
^{t}Bu	2.31	376	28.1	87699	87.7
				average ΔG =	87.7

Eyring analysis of this rotational barrier (87.7 kJ/mol) was conducted using equations se2-se3 to obtain an approximate half-life of the atropisomer.

$$k = \kappa \frac{k_B T}{h} e^{\frac{-\Delta G^{\ddagger}}{RT}} \qquad \text{(eq. se2)}$$

$$t_{1/2} = \ln \frac{2}{k} \qquad \text{(eq. se3)}$$

Where k_B is the Boltzmann constant, T is temperature (e.g. 298 K), h is the Planck constant, R is the ideal gas constant and k is the rate constant, and the transmission constant κ is assumed to be 1. As is common, this analysis assumed ΔS = 0.

The half-life $t_{1/2}$ of the syn-anti atropisomers varies between 1.8 min at 298 K and 84 ms at 373 K.

Synthesis of anthracene cage **4**

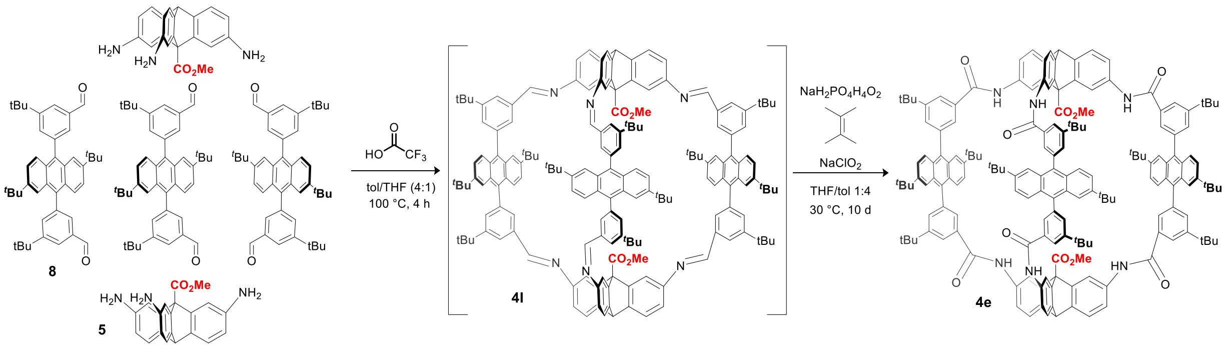

Anthracene cage 4i: Trisamino triptycene **5** (28.7 mg, 80.3 µmol) was dissolved in THF (15 mL) and added was 30 mL of a stock solution [toluene (100 mL) containing trifluoroacetic acid (1 µL)]. Anthracene bisaldehyde **8** (73.6 mg, 120 µmol) was added as a solid, and the reaction heated to 110 °C. The solid dissolved after a few minutes. After stirring at 110 °C for 4 h, the reaction mixture was analysed by MALDI-ToF and analytical GPC, showing high conversion to a uniformly sized species with mass consistent with the imine cage. The reaction was cooled to 21 °C and added immediately subjected to the Pinnick oxidation reaction.

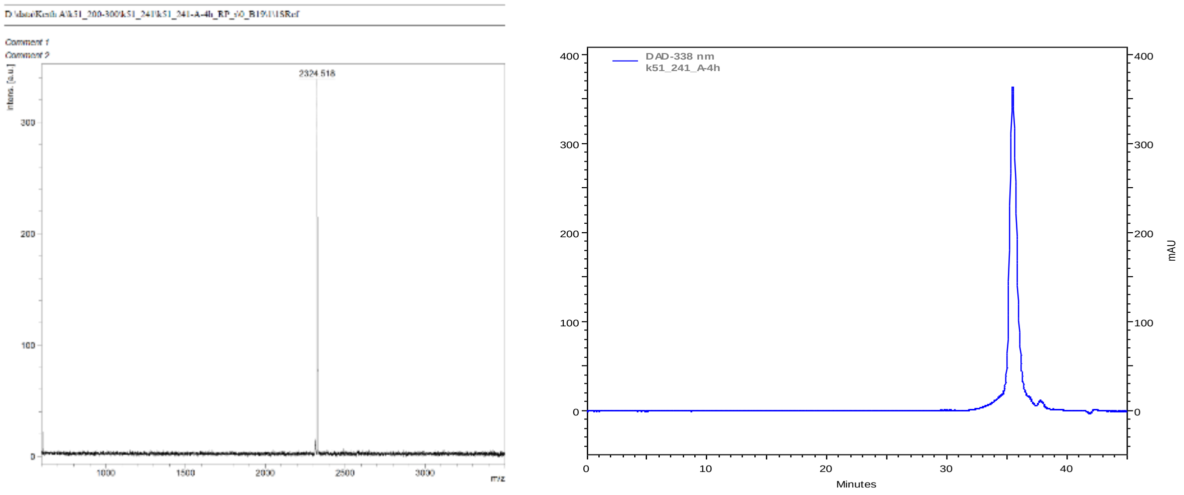

***Figure S10.** MALDI-TOF and analytical GPC showing high conversion to anthracene imine cage **4i**.*

Anthracene dimethyl ester cage 4e: To the solution of anthracenyl hexaimine cage **4i** (max: 97.9 mg, 40.1 µmol) at 30 °C was added 2,3-dimethyl-2-butene (328 µL, 180 eq), sodium chlorite (208 mg, 2.30 mmol, 150 eq), and anhydrous sodium dihydrogen phosphate (309 mg, 168 eq.). The heterogeneous reaction was stirred vigorously in the dark for 10 days. The reaction was diluted with ethyl acetate and water and extracted three times with ethyl acetate. The organics were washed with aqueous sodium bicarbonate and brine, and the resulting organics purified by recycling gel-permeation chromatography (THF, 6 x 90 min cycles). The resulting solid was triturated with diethyl ether (to remove impurities from the THF) to give a yellow solid **4e** (62.5 mg, 61% over two steps) with data indicating a mixture of regioisomers (estimated at 2:1): **^{1}H NMR** (500 MHz, DMSO-d_6) δ 10.17 – 10.09 (m, 6H), 8.30 – 8.23 (m, 6H), 8.16 – 8.02 (m, 12H), 7.86 (dd, *J* = 11.8, 1.9 Hz, 3H), 7.73 (t, *J* = 2.0 Hz, 3H), 7.68 – 7.62 (m, 6H), 7.58 – 7.39 (m, 24H), 5.63 – 5.59 (m, 2H), 3.88 – 3.84 (m, 6H), 1.39 (s, 54H), 1.14 – 1.10 (m, 54H); **AT-IR** (neat, cm^{-1}): 2961, 2905, 2869, 1746, 1673, 1627, 1595, 1519, 1475, 1397; **MS *m/z*** (MALDI-ToF-RP) 2533.1 ($C_{176}H_{176}N_6O_{10}$, M$^+$ requires 2533.3).

*Hydrolysis of anthracene cage **4e** to form **4***

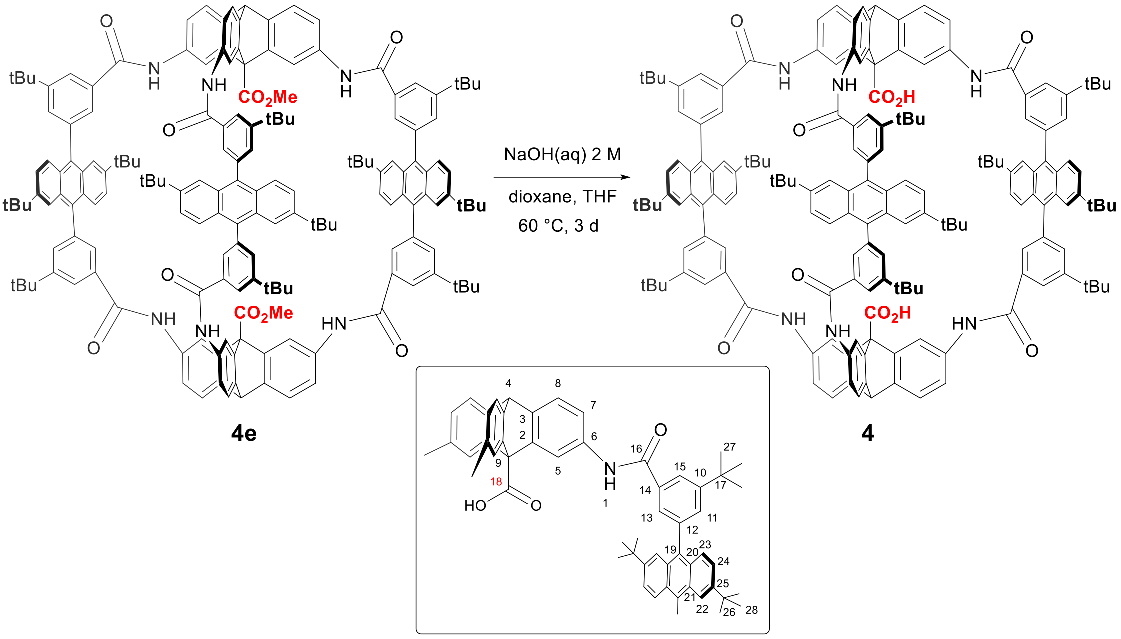

Anthracene cage 4: Anthracene dimethyl ester cage **4e** (20.0 mg, 7.89 µmol) was dissolved in dioxane (0.35 mL) and THF (0.4 mL). Added was aqueous sodium hydroxide (0.6 mL, 2 M). The reaction was stirred at 60 °C for 3 d, adding more THF if the cage was seen to precipitate. When TLC and MALDI-ToF analysis (after aliquot acidification) indicated full conversion, the reaction was cooled, diluted with ethyl acetate, and acidified to pH 6 with aqueous HCl. The aqueous layer was extracted with ethyl acetate, and the combined organic layers washed with water. The organics were dried over magnesium sulfate, filtered, concentrated, and the resulting residue triturated with petrol/diethyl ether (9:1) to give a pale-yellow solid (19 mg, 95%). The solid has a fluorescent purple tinge in solution. The data, given below, indicate a ~1:3 statistical mixture of the two expected isomers.

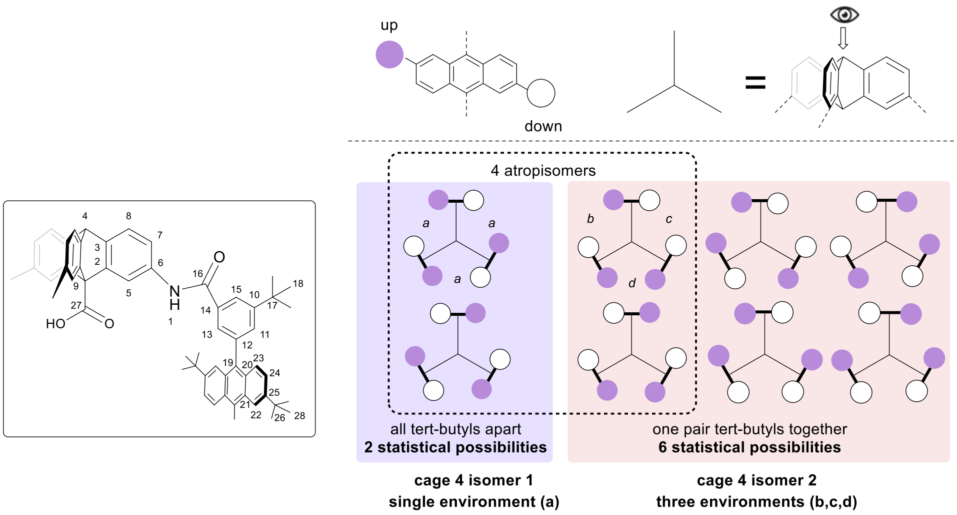

Figure S11. *Depiction of the possible atropisomers of tert-butyl anthracene units in cage **4e**.*

The anthracene cage is expected to appear as a pair of atropisomers by NMR-spectroscopy due to restricted rotation around the anthracene-phenyl bonds, as observed for the anthracenyl precursor **8**. These isomers differ in the position of the anthracenyl tert-butyl groups relative to each other: tert-butyl vectors can be alternating (apart), or two can clash (together). There are 8 possible statistical configurations (four of which are degenerate by rotation/symmetry) corresponding to a 1:3 statistical

mixture of alternating:clashing. Of the remaining four species, there are two pairs of enantiomers (so two spectrally distinct species). ^{1}H-NMR analysis shows up to four environments per atom, consistent with **isomer1**:**isomer2** in a 1:3 ratio. Given **isomer2** has different 3-edge environments, this means the mixture is roughly statistical with no significant energy preference between isomers **1**&**2** (assuming interconversion is possible): **^{1}H NMR** (600 MHz, THF-d_8) δ 9.62 – 9.46 (m, 6H, H^{1}), 8.39 – 8.28 (m, 6H^{15}), 8.13 (d, *J* = 1.7 Hz, ~1.5H^{5}), 8.11 (d, *J* = 1.6 Hz, ~1.5H^{5}), 8.06 (d, *J* = 2.1 Hz, ~1.5H^{5}), 8.05 (d, *J* = 2.0 Hz, ~1.5H^{5}), 8.00 – 7.96 (m, 2H, 4H^{7}), 7.96 – 7.95 (m, 4H^{13}), 7.95 – 7.93 (m, 2H, 2H^{13}), 7.93 – 7.90 (m, 2H, 2H^{7}), 7.67 – 7.64 (m, 6H, H^{11}), 7.62 (d, *J* = 9.4 Hz, 6H^{23}), 7.59 – 7.53 (m, 6H, H^{24}), 7.43 – 7.39 (m, 6H, H^{22}), 7.40 – 7.34 (m, 6H, H^{8}), 5.43 – 5.39 (m, 2H, H^{4}), 1.46 – 1.41 (m, 54H, H^{18}), 1.19 – 1.13 (m, 54H, H^{28}); **^{13}C NMR** (151 MHz, THF-d_8) δ 171.13, 171.11 (C^{27}); 165.33, 165.31, 165.28, 165.26 (C^{16}); 152.69 – 152.61 (m, C^{10}); 147.99 (C^{25}), 144.89, 144.86, 144.80, 144.78 (C^{3}); 142.72, 142.56, 142.54, 142.39 (C^{2}); 139.96, 139.92, 139.87 (C^{12}); 137.64, 137.59, 137.58, 137.54 (C^{6}); 137.34, 137.31 (C^{14}); 136.12, 136.10, 136.08, 136.07 (C^{19}); 132.69, 132.66, 132.65, 132.59 (C^{11}); 130.72, 130.71, 130.69 (C^{21}); 129.67 (C^{20}); 127.97, 127.92, 127.89 (C^{13}); 127.58 – 127.48 (m) (C^{23}); 125.57 (C^{22}); 125.27, 125.21, 125.17 (C^{15}); 123.94, 123.90, 123.87 (C^{8}); 121.93, 121.91, 121.88 (C^{24}); 117.99, 117.93, 117.87 (C^{7}); 117.52, 117.44, 117.39, 117.35 (C^{5}); 63.16, 63.14 (C^{9}); 53.97 (C^{4}); 35.92 (C^{17}); 35.72 – 35.64 (m) (C^{26}); 31.86 (C^{18}); 31.22, 31.21, 31.20 (C^{28}); **AT-IR** (neat, cm^{-1}): 3656, 2981, 2971, 2888, 2360, 2341, 1664, 1595, 1521, 1473, 1383; **MS *m/z*** (MALDI-ToF-RP) 2505.1 ($C_{174}H_{172}N_6O_{10}$, M^+ requires 2505.3).

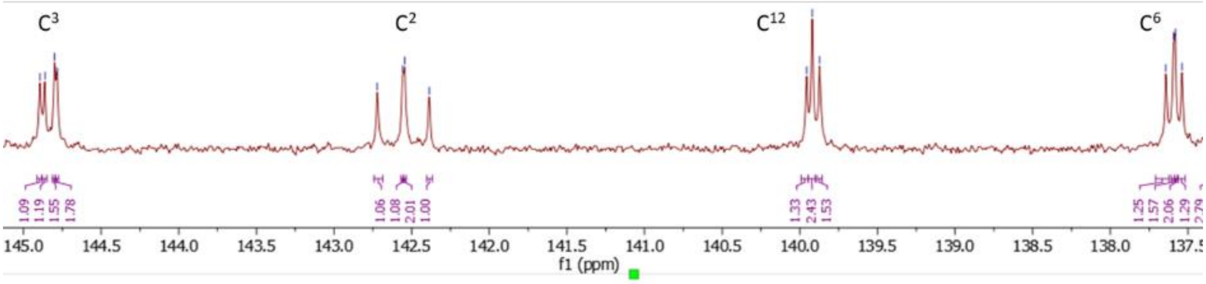

Figure S12. *Selected regions of ^{13}C-NMR spectra of cage* ***4*** *displaying evidence of the two isomers. Examples of carbon environments with 4 signals, each signal displaying similar integration. This indicates a 3:1 ratio of isomer2:isomer1, where isomer 2 has three environments as expected, and isomer 1 has 1 environment.*

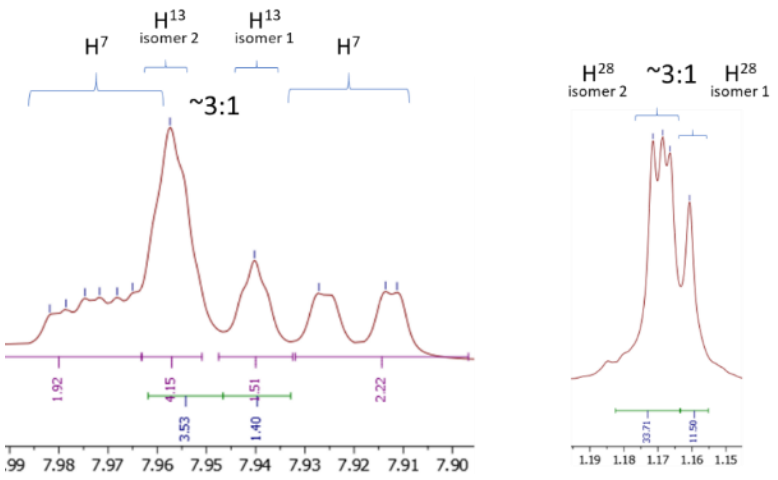

Figure S13. *Selected regions of* ^{1}*H-NMR spectra of cage* ***4*** *displaying evidence of the two isomers.* Examples of proton signals from ^{1}H-NMR of cage **4**, split into roughly 3:1 groups, indicating a 1:1 ratio of isomer1:isomer2.

Binding titrations of cages **1** and **4** with bisamines

Synthesis of guests for binding titrations

All guests were purchased and used as received, except **G3**:

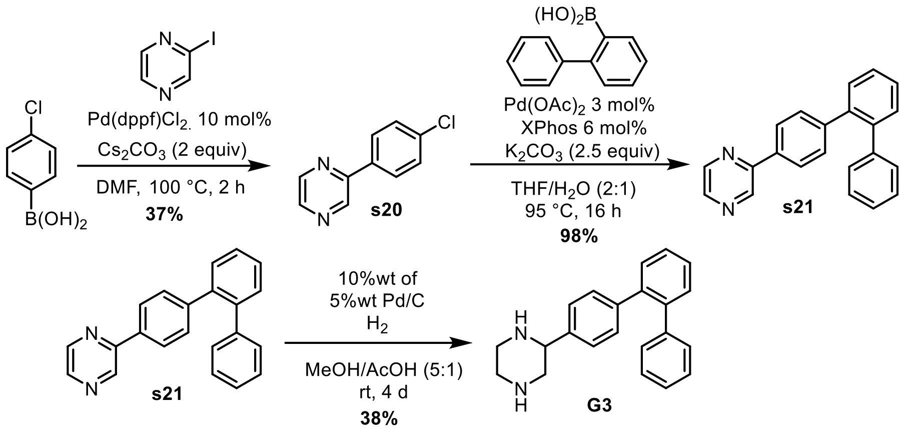

2-(4-chlorophenyl)pyrazine s20: (4-Chlorophenyl)boronic acid (0.160 g, 1.02 mmol), 2-iodopyrazine (211 mg, 1.02 mmol), cesium carbonate (667 mg, 2.05 mmol) and [1,1′-bis(diphenylphosphino)ferrocene]dichloropalladium(II), complex with dichloromethane (83.6 mg, 102 µmol) were dissolved in dry *N,N*-dimethylformamide (1.1 mL), degassed briefly with 3 vacuum/argon cycles, and then refluxed with stirring at 100 °C for 2 h. The solution was cooled, diluted with ethyl acetate, and washed with brine x3, dried over magnesium sulfate, filtered and concentrated under reduced pressure. The residue was purified by flash column chromatography (0-40% EtOAc/petrol) to give **s20** as an off-white solid, 2-(4-chlorophenyl)pyrazine (73 mg, 0.38 mmol, 37%) with data consistent with the literature.[15] **^{1}H NMR** (400 MHz, $CDCl_3$) δ 9.02 (d, *J* = 1.5 Hz, 1H), 8.64 (dd, *J* = 2.5, 1.5 Hz, 1H), 8.53 (d, *J* = 2.5 Hz, 1H), 8.02 – 7.94 (m, 2H), 7.53 – 7.43 (m, 2H); **^{13}C NMR** (126 MHz, $CDCl_3$) δ 151.9, 144.4, 143.1, 141.9, 136.5, 134.8, 129.5, 128.4; **MS** ESI(+): 191.0, 193.0, ($C_{10}H_8ClN_2$, MH^+ requires 191.0, 193.0).

2-([1,1':2',1''-terphenyl]-4-yl)pyrazine s21 2-(4-Chlorophenyl)pyrazine (71.0 mg, 372 µmol), [1,1'-biphenyl]-2-ylboronic acid (73.8 mg, 372 µmol), palladium(II) acetate (2.51 mg, 3 mol%, 11.2 µmol), and XPhos (10.7 mg, 6 mol%, 22.3 µmol) were combined in a flask with THF (2 mL). The mixture was degassed with 3x vacuum/argon cycles, and added was potassium carbonate (129 mg, 931 µmol) dissolved in water (1 mL). The reaction was stirred at 95 °C for 16 h, cooled, extracted with ethyl acetate, and the organics washed with saturated aqueous sodium bicarbonate solution and brine, dried over magnesium sulfate, filtered and concentrated. The crude residue was purified by flash column chromatography (Biotage, sfar, 10 g, 0-25% EtOAc/petrol) to yield an off-white solid **s21** (112 mg, 98%); **^{1}H NMR** (400 MHz, $CDCl_3$) δ 8.99 (d, *J* = 1.4 Hz, 1H), 8.59 (dd, *J* = 2.5, 1.6 Hz, 1H), 8.46 (d, *J* = 2.5 Hz, 1H), 7.94 – 7.85 (m, 2H), 7.52 – 7.39 (m, 4H), 7.34 – 7.28 (m, 2H), 7.27 – 7.15 (m, 5H); **^{13}C NMR** (126 MHz, $CDCl_3$) δ 152.6, 144.2, 143.5, 142.8, 142.1, 141.3, 140.7, 139.7, 134.4, 130.8, 130.7, 130.6, 130.0, 128.1, 128.0, 127.7, 126.7, 126.5; **HR-MS *m/z*** (ESI+): 309.1385 ($C_{22}H_{17}N_2$, $[M+H]^+$ requires 309.1386).

2-([1,1':2',1''-terphenyl]-4-yl)piperazine G3: 2-([1,1':2',1''-terphenyl]-4-yl)pyrazine (110 mg, 357 µmol) was suspended in methanol (1.8 mL) and acetic acid (0.4 mL) and put under an atmosphere of argon. Added was palladium(5% wt)/carbon (11 mg, 357 µmol), and the mixture put under a hydrogen atmosphere with three vacuum/hydrogen cycles. The reaction was then stirred vigorously for 4 d under a double hydrogen balloon positive pressure at 21 °C. After this time, the mixture was carefully filtered through Celite (preventing the Pd residue from drying out) under a nitrogen funnel, and the residue concentrated, redissolved in ethyl acetate, washed with saturated aqueous sodium bicarbonate solution, dried over magnesium sulfate, filtered and concentrated. The residue was purified by flash

column chromatography (0-100% EtOAc/petrol; then 0-20% methanol/DCM+2% pyridine) to give **G3** a pale yellow oil (43.0 mg, 137 µmol, 38%). **^{1}H NMR** (500 MHz, $CDCl_3$) δ 7.49 – 7.35 (m, 4H), 7.25 – 7.15 (m, 5H), 7.15 – 7.05 (m, 4H), 4.01 (d, *J* = 10.1 Hz, 1H), 3.32 – 3.10 (m, 4H), 3.10 – 2.92 (m, 1H), 2.82 (t, *J* = 11.4 Hz, 1H); **^{13}C NMR** (126 MHz, $CDCl_3$) δ 141.7, 141.5, 140.7, 140.0, 138.4, 130.8, 130.6, 130.3, 130.0, 128.0, 127.7, 127.6, 126.7, 126.6, 59.0, 51.3, 45.1, 44.4; **HR-MS *m/z*** (ESI+): 315.1859 ($C_{22}H_{23}N_2$, $[M+H]^+$ requires 315.1856).

^{1}H-NMR Binding Titration Experiments - Protocol

Host-guest binding events were measured by ^{1}H-NMR (Bruker AVIII HD 500).

Outline method: a solution containing cage host (0.251-0.555 mM) and guest was titrated by microsyringe into a solution of host and internal standard (1,1,2,2-tetrachloroethane, δ 6.5074 ppm (2H, s) in THF-d_8) in an NMR tube such that the concentration of host remained constant. The tube was equilibrated by vigorous shaking, and ^{1}H-NMR spectra (16 scans, T=298.15 K) were recorded between titration additions, with automatic locking/shimming for each measurement. The total volume varied between 0.55 and ~1.1 mL, and the concentration of guest varied from zero to ~20-30 equivalents, depending on rough binding strength from a preliminary titration. Approximately 16 data points per titration were recorded, with the highest density of points recorded close to the “knee” as determined by a preliminary titration. A minimum of three consistent titrations were performed for each host/guest combination. ^{1}H-NMR chemical shift changes (Δδ) were determined for multiple cage proton environments. The guest shifts were largely too broad or obscured by solvent to use. Binding was in fast exchange (only one set of chemical environments was observed for the cage/guest) on the ^{1}H-NMR (500 MHz) timescale. The “Alignment Shifts Graph” advanced data-processing facility in the MestReNova software (v 14.2, x86_64) was used to aid extraction of ^{1}H-NMR chemical shift changes (Δδ). Individual binding curves were checked by eye to identify extraction errors. These Δδ shift changes were fit as a function of host-guest concentration using equilibrium binding models as enacted in the ReactLab software (v1.1, Build 11). The concentration of host was reappraised relative to the internal standard. Within the ReactLab software, global fitting of multiple Δδ shift changes (minimum of 2 per titration) to 1:1 and 1:2 host:guest binding models was trialled. For equilibrium constants >10^4 M^{-1}, good fitting required allowing the host concentration to be fit as an additional parameter starting from the “known” concentration (a common and powerful technique).[16] ^{1}H-NMR titrations can provide accurate binding constants up to about 10^5 M^{-1}, but the use of sub-millimolar concentrations of cage mean values on the order of 10^6 M^{-1} are still informative.

Detailed method: All solids and liquids were accurately weighed to ±0.00005 g. Cage compound (0.70-1.20 mg) was dissolved in THF-d_8 (~1.1000 mL, weighed). This solution was divided into two by weight, to give solution A and solution B. To solution A (host) was added ~28.00 µL (weighed) of a stock solution of internal standard 1,1,2,2-tetratchloroethane (~1.000 µL, weighed, dissolved in THF-d_8 ~1.000 mL, weighed). To solution B (host/guest) was added ~28.00 µL (weighed) of a stock solution of guest. This gave two solutions with equal and known concentrations of host. All accurate weights from the volumes given were used to calculate the theoretical concentration of host and guest and internal standard. The titrations were performed by manual addition of aliquots of solution B (host/guest) (1.0-100.0 µL aliquots) to solution A in an NMR tube. All solutions were kept in small, tightly sealed vials to avoid evaporation losses, and were prepared directly before use.

Values of ΔG were calculated according to equation se4, where *T* is temperature (298.15 K) and *R* is the ideal gas constant (8.314 J/mol/K).

$$\Delta G = -RT\ln K \quad \text{(eq. se4)}$$

Titration Binding Data Summary

Several fitting protocols are provided here to contextualise the goodness of the fitting to the different models and how allowing different parameters to fit changes the result. The results are consistent enough that no changes in conclusions occur between approaches. The data presented in the tables below are as follows:

- **Table S2:** Terphenyl cage **1**, fit to a 1:1 host:guest model, with some host concentration fitting allowed.
- **Table S3:** Terphenyl cage **1**, fit to a 1:2 host:guest model, with some host concentration fitting allowed.
- **Table S4:** Terphenyl cage **1**, fit to a 1:1 host:guest model, with ligand concentration fitting allowed.
- **Table S5:** Terphenyl cage **1**, fit to a 1:2 host:guest model, with ligand concentration fitting allowed.

- **Table S6:** Anthracene cage **4**, fit to a 1:1 host:guest model, with some host concentration fitting allowed.
- **Table S7:** Anthracene cage **4**, fit to a 1:2 host:guest model, with some host concentration fitting allowed.
- **Table S8:** Anthracene cage **4**, fit to a 1:1 host:guest model, with ligand concentration fitting allowed.
- **Table S9:** Anthracene cage **4**, fit to a 1:2 host:guest model, with ligand concentration fitting allowed.

NOTES: *Confidence intervals (error estimation) are typically large for strong binding guests close to the binding sensitivity limit of NMR. Values for weaker secondary associations (K_2) are small in relative magnitude, but large as quoted by % due to their strong dependence on the variance in the strong binding constant, K_1. The binding constants quoted are stepwise for the first and then second binding events. Although the confidence intervals are variable, there is typically a very good agreement between constants calculated by fitting the guest, fitting the host, and between 1:1 and 1:2 models. These data are therefore considered sensible and consistent with a strong initial binding, and a weaker secondary binding. The number of NMR signals used in the fitting is shown for each guest under "shifts fit", and was determined by clear available shifts for a given host:guest combination.*

Table S2. *Titration binding data for cage* ***1*** *with* ***G1-3*** *fitting to a 1:1 model. Host concentration fitting allowed as indicated.*

Entry	GUEST	shifts fit	K_1 (M^{-1}) [b]		ΔG_1 (kJ/mol)
1 [a]	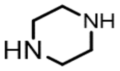	2	1.31E+05	*(± 29.4%)*	-29.2
2	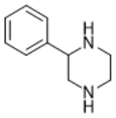	1	6.21E+03	*(± 5.3%)*	-21.7
3	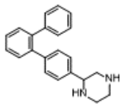	2	1.75E+03	*(± 35.2%)*	-18.5

[a] *host concentration was a fitted parameter;* [b]*error quoted as confidence internal at 95% for three data points.*

Table S3. *Titration binding data for cage* ***1*** *fitting to a 1:2 (host:guest) model. Host concentration fitting allowed for strongly binding guests.* K_1 *and* K_2 *are stepwise association constants.*

Entry	GUEST	shifts fit	K_1 (M^{-1}) [b]		K_2 (M^{-1}) [b]		ΔG_1 (kJ/mol)
1 [a]	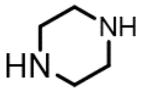	2	1.89E+05	*(± 12.5%)*	3.08E+02	*(± 81.5%)*	-30.1
2 [a]	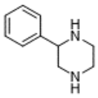	4	6.08E+03	*(± 7.8%)*	4.42E+01	*(± 29.5%)*	-21.6
3 [a]	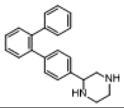	5	1.63E+03	*(± 51.6%)*	1.21E+02	*(± 81.6%)*	-18.3

[a] *host concentration was a fitted parameter;* [b]*error quoted as confidence internal at 95% for three data points.*

Table S4. *Titration binding data for cage **1** fitting to a 1:1 (host:guest) model. Ligand concentration fitting performed for all guests. [host concentration calculated by internal standard].*

Entry	GUEST	shifts fit	K_1 (M^{-1}) ^{b}	ΔG_1 (kJ/mol)
2	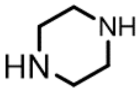	2	1.01E+05 *(± 28.9%)*	-28.6
5	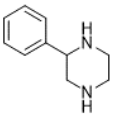	1	6.65E+03 *(± 12.2%)*	-21.8
6	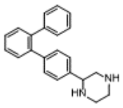	2	2.52E+04 *(± 16.6%)*	-25.1

[b]error quoted as confidence internal at 95% for three data points.

Table S5. *Titration binding data for cage **1** fitting to a 1:2 (host:guest) model. Ligand concentration fitting performed for all guests. [host concentration calculated by internal standard].*

Entry	GUEST	shifts fit	K_1 (M^{-1}) ^{b}		K_2 (M^{-1}) ^{b}		ΔG_1 (kJ/mol)
2	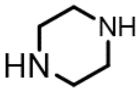	2	1.45E+05	*(± 5.7%)*	2.30E+02	*(± 74.6%)*	-29.5
5	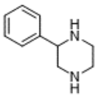	4	6.04E+03	*(± 13.7%)*	4.34E+01	*(± 23.8%)*	-21.6
6	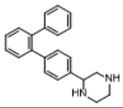	5	8.35E+03	*(± 31.5%)*	2.68E+02	*(± 92.9%)*	-22.4

[b]error quoted as confidence internal at 95% for three data points.

Table S6. *Titration binding data for anthracene cage **4** fitting to a 1:1 (host:guest) model. Host concentration fitting allowed for strongly binding guests.*

Entry	GUEST	shifts fit	K_1 (M^{-1}) [b]		ΔG_1 (kJ/mol)
1[a]	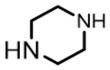	2	6.82E+05	*(± 28.0%)*	-33.3
2	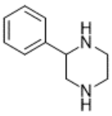	1	1.85E+04	*(± 57.6%)*	-24.3
3	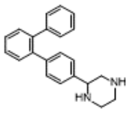	2	4.31E+02	*(± 8.3%)*	-15.0

[a] *host concentration was a fitted parameter;* [b]*error quoted as confidence internal at 95% for three data points.*

Table S7. *Titration binding data for anthracene cage **4** fitting to a 1:2 (host:guest) model. Host concentration fitting allowed for strongly binding guests.*

Entry	GUEST	shifts fit	K_1 (M^{-1}) [b]		K_2 (M^{-1}) [b]		ΔG_1 (kJ/mol)
1[a]	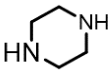	4	7.10E+05	*(± 40.3%)*	9.63E+01	*(± 133%)*	-33.4
2 [a]	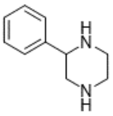	2,4,4	1.49E+04	*(± 16.9%)*	1.05E+01	*(± 180%)*	-23.8
3	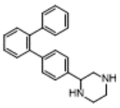	6	3.01E+02	*(± 37.6%)*	7.76E+01	*(± 122%)*	-14.1

[a] *host concentration was a fitted parameter;* [b]*error quoted as confidence internal at 95% for three data points.*

Table S8. *Titration binding data for anthracene cage **4** fitting to a 1:1 (host:guest) model. Ligand concentration fitting performed for all guests. [host concentration calculated by internal standard].*

**Entry**	**GUEST**	**shifts fit**	**K_1 (M^{-1})** [b]		**ΔG_1 (kJ/mol)**
1	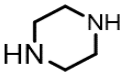	2	8.14E+05	*(± 62.6%)*	-33.7
2	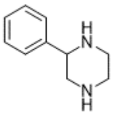	1	1.86E+04	*(± 28.4%)*	-24.4
3	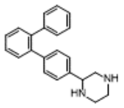	2	9.63E+02	*(± 8.5%)*	-17.0

[b]*error quoted as confidence internal at 95% for three data points.*

Table S9. *Titration binding data for anthracene cage **4** fitting to a 1:2 (host:guest) model. Ligand concentration fitting performed for all guests. [Host concentration calculated by internal standard].*

**Entry**	**GUEST**	**shifts fit**	**K_1 (M^{-1})** [b]		**K_2 (M^{-1})** [b]		**ΔG_1 (kJ/mol)**
1	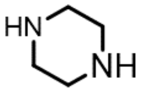	4	8.71E+05	*(± 75.9%)*	7.67E+01	*(± 131%)*	-33.9
2	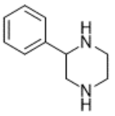	4	2.20E+04	*(± 18.6%)*	1.49E+01	*(± 177%)*	-24.8
3	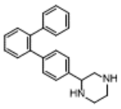	6	7.26E+02	*(± 34.1%)*	1.82E+02	*(± 149%)*	-16.3

[b]*error quoted as confidence internal at 95% for three data points.*

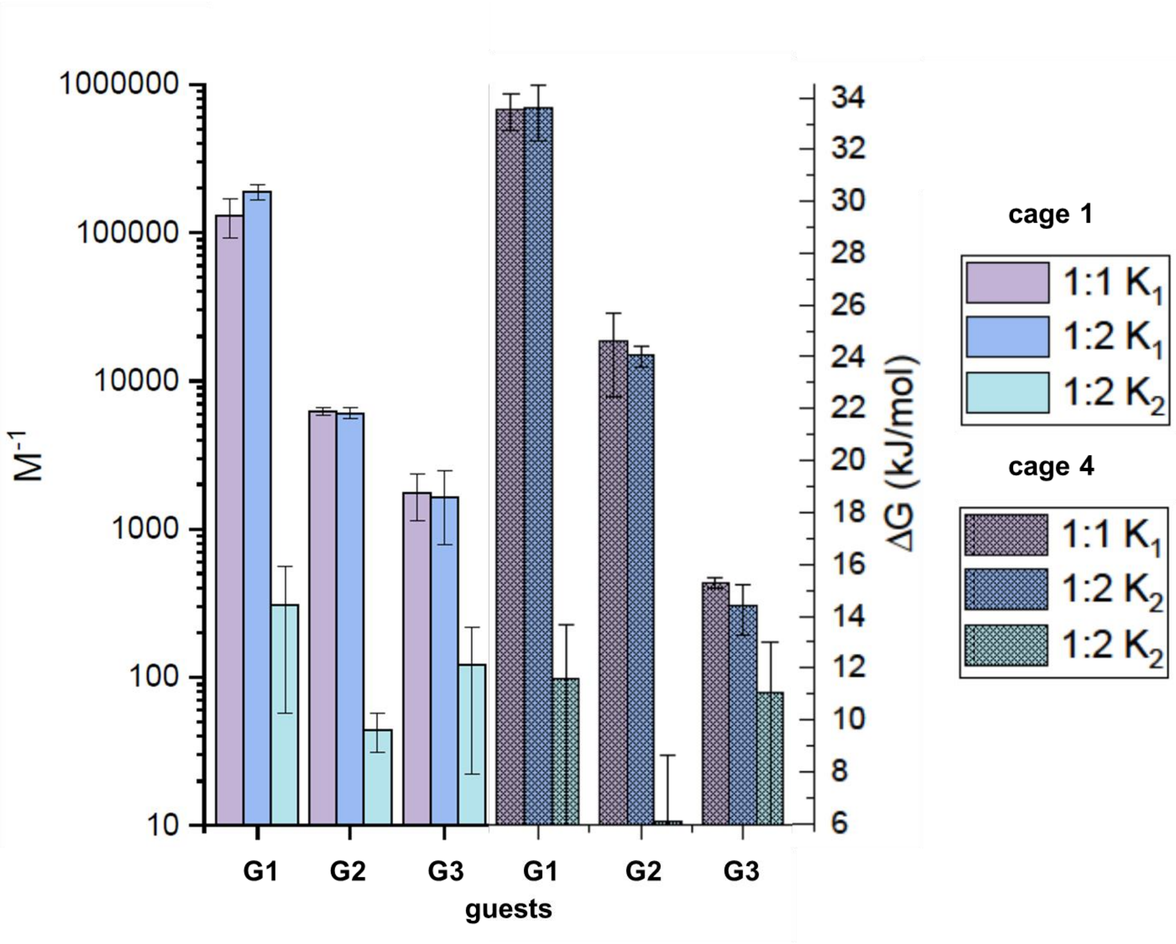

Figure S14. *^{1}H-NMR binding constant* data *(THF-d8) for the guests with cage* ***1*** *or cage* ***4*** *(with errors shown as 95% confidence intervals from 3 measurements) with data fit to 1:1 or 1:2 host:guest equilibrium binding models, plotted on a log scale.*

Example of titration data, fitted values, and chemical shift changes

The following titration protocol (cage **1** with **G2**) is representative.

1. Perform titration as described above, and process ^{1}H-NMR spectra with phase correction and baseline correction in MestReNova. (v. 14.2.0-26256, released 2020-09-25)

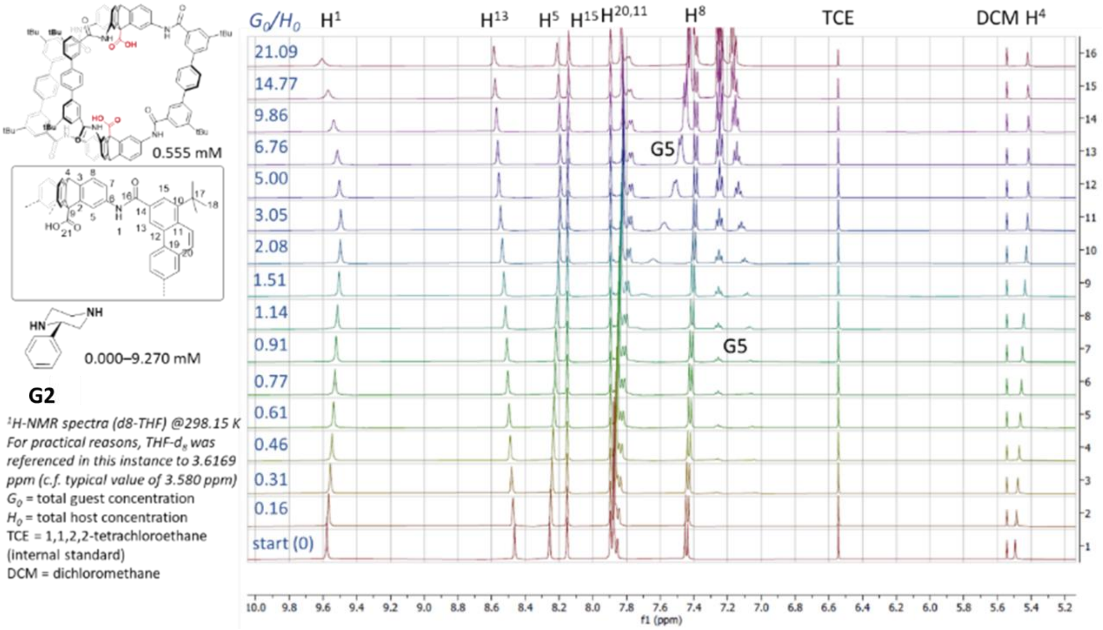

Figure S15. *Example ^{1}H-NMR host-guest binding titration data*

2. Extract shift changes for all non-obscured peaks using "Alignment Shifts Graph" tool in MestReNova, which peak-fits each included signal in a stack of spectra, follows the shift changes for each included signal between spectra, and reports a table of Δδ ppm values against the internal time metadata for the spectra. This data is replotted against the ratio of total guest concentration ($[G]_0$) to total host concentration $[H_0]$.

Table. S10. Example of host-guest binding titration chemical shift data.

	Δδ ppm values from starting shifts (ppm) of cage signals						
$[G]_0/[H]_0$	10.5	8.5	8.23	8.15	7.8	7.5	5.5
0.00	0.0000	0.0000	0.0000	0.0000	0.0000	0.0000	0.0000
0.16	0.0104	0.0060	0.0094	0.0025	0.0106	0.0083	0.0097
0.31	0.0208	0.0118	0.0183	0.0050	0.0223	0.0165	0.0188
0.46	0.0308	0.0172	0.0273	0.0075	0.0329	0.0239	0.0275
0.62	0.0400	0.0222	0.0351	0.0075	0.0424	0.0309	0.0354
0.77	0.0479	0.0267	0.0425	0.0100	0.0509	0.0374	0.0427
0.92	0.0549	0.0309	0.0488	0.0125	0.0588	0.0431	0.0490
1.14	0.0629	0.0363	0.0562	0.0125	0.0668	0.0496	0.0566
1.51	0.0726	0.0427	0.0655	0.0175	0.0721	0.0583	0.0664
2.08	0.0806	0.0493	0.0745	0.0226	0.0774	0.0670	0.0758
3.05	0.0827	0.0562	0.0808	0.0276	0.0827	0.0740	0.0827
4.99	0.0742	0.0632	0.0827	0.0376	0.0827	0.0797	0.0817
6.76	0.0620	0.0676	0.0808	0.0451	0.0827	0.0814	0.0806
9.86	0.0406	0.0728	0.0761	0.0551	0.0827	0.0827	0.0792
14.77	0.0073	0.0784	0.0671	0.0702	0.0822	0.0827	0.0782
21.10	-0.0287	0.0827	0.0570	0.0827	0.0811	0.0666	0.0771

3. Globally fit selected data to 1:1 or 1:2 models in ReactLab, with or without host or guest fitting, as described above. Data for 1:1 fitting selected on basis of not displaying evidence of secondary binding interference (i.e. 1:1 fitting uses shift data with clear asymptote, e.g. top right trace in diagram below, which is the triptycene bridgehead proton). Otherwise, data selected if extraction from spectra gives a clean curve. This process provided association constants, K_1, K_2.

Reactants	Reaction Type	Products	Label	Parameters logK/logβ	±	Fit ☑
M+L	=	ML		3.818	0.014	☑
ML+L	=	ML2		1.495	0.045	☑

Label	Auxiliary Parameters	±	Fit ☑
M[beaker]	0.000555	0.000	☑
L[burette]	0.020210		☐
M[burette]	0.000551		☐

n_species	4
n_par	2
n_aux_par	3

σr	3.22E-04
ssq	4.97E-06

logKw	-14.000

Species	M	L	ML	ML2
Spectrum	known	non-abs	colored	colored
Init[ ]	M	L		
Beaker	5.55E-04	0.0000		
Burette	0.000551	0.020210		
Vtot (ml)	0.5692			

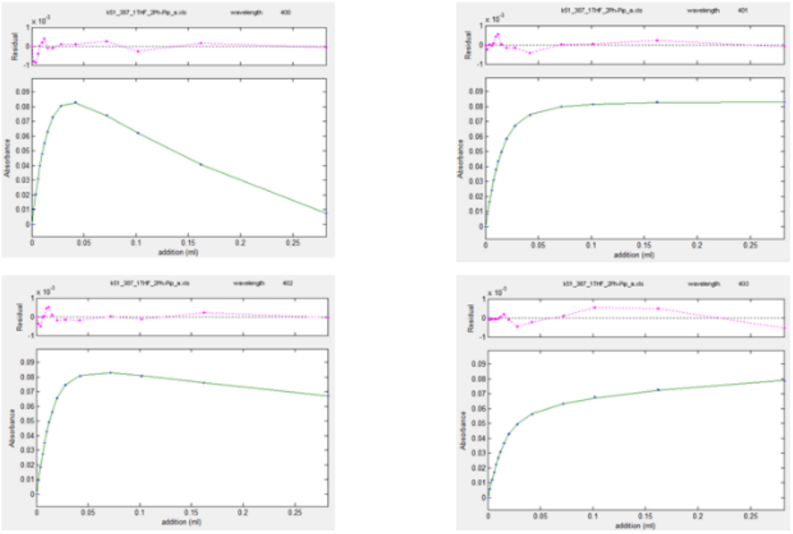

Figure S16. *Fitting the triptycenyl bridgehead proton to the 1:1 model. The top-right graph of the four shows the clean 1:1 binding curve.*

4. Data fits checked to ensure residual errors are small, and don't contain significant systematic trends (pink lines in traces above).

Conformational studies of cage **1**

Demonstration of cage **1** conformer permutations – **C1-C13**

The cage carbonyls can be pointing in (1) or out (0) in the most stable trans ~planar amide geometry. Each of the three edge pieces has a pair of carbonyls that defines the geometry of that edge. For four types of edge piece, *n*, [11,00,10,01], and three edges, *r*, where the order of the three items is unimportant (by axial rotation) but order within an edge is important (top/bottom), there are 20 permutations of cage conformer, *p*, by:

$$p = \frac{(r+n-1)!}{r!\,(n-1)!}$$

FULL LIST: {11,11,11} {11,11,00} {11,11,10} {11,11,01} **{11,00,00}** {11,00,10} {11,00,01} {11,10,10} **{11,10,01}** {11,01,01} **{00,00,00}** {00,00,10} {00,00,01} {00,10,10} **{00,10,01}** {00,01,01} {10,10,10} {10,10,01} {10,01,01} {01,01,01}

Of these 20, 6 do not map to another by rotation, and 14 have degenerate pairs by rotation.

UNIQUE: {11,11,11} {00,00,00} {11,11,00} {11,00,00} {11,10,01} {00,10,01}

DEGENERATE PAIRS: {11,11,10} {11,11,01} - {10,10,10} {01,01,01} - {11,10,10} {11,01,01} - {00,10,10} {00,01,01} - {10,10,01} {10,01,01} - {00,00,10} {00,00,01} - {11,00,01} {11,00,10}

FINAL 13 UNIQUE CAGE CONFORMERS: {11,11,11} {00,00,00} {11,11,00} {11,00,00} {11,10,01} {00,10,01} {11,11,10} {10,10,10} {11,10,10} {00,10,10} {10,10,01} {00,00,10} {11,00,10}

STATISTICAL SYMMETRY: If all cage conformers were freely interconverting and had the same energy, the populations would nonetheless be skewed due to statistics, calculated by their symmetry/degeneracy. These degeneracies were used in Boltzmann weighting of cages.

(for instance {00,00,10} can be {00,00,01} {00,01,00} {00,10,00} {01,00,00} {10,00,00})

degeneracy = 1 : {00,00,00} {11,11,11}

degeneracy = 2 : {10,10,10}

degeneracy = 3 : {11,11,00} {11,00,00}

degeneracy = 6 : {11,10,01} {00,10,01} {11,11,10} {11,10,10} {00,10,10} {10,10,01} {00,00,10}

degeneracy = 12 : {11,00,10}

The 13 conformers **C1-13** were formalised as their “lowest” ordered state (e.g. {10,11,00} is the same conformer as {00,01,11}, which is the alphabetically lowest representation). It is possible to convert degenerate codes into these lowest ordered states using this pseudo code:

Input data: $[\#_t\#_b][\#_t\#_b][\#_t\#_b]$ *where # = 0 (out carbonyl) or 1 (in carbonyl), and each* $[\#_t\#_b]$ *is one edge piece, and the first index* $\#_t$ *of each [##] is the ‘top’ carbonyl, and the second index* $\#_b$ *is the ‘bottom’ carbonyl.*

1) For each $[\#_t\#_b][\#_t\#_b][\#_t\#_b]$, if count([10]) > count([01]), then for each $[\#_b\#_b]$, set $[\#_t\#_b]$ = $[\#_b\#_t]$ *equivalent to inverting the cage (so top becomes bottom) to alphabeticise .*
2) Sort($[\#_t\#_b][\#_t\#_b][\#_t\#_b]$) alphabetically (i.e. [00],[0,1],[1,0],[1,1]). *Equivalent to rotating the cage along the long axis so the lowest alphabetic state is read first, or performing a reflection to swap the order of two edge pieces.*

Example: step 1: {10,11,00} becomes {01,11,00}

Example: step 2: {01,11,00} becomes {00,01,11}

We then assigned conformer numbers after sorting these 13 states from high to low.

Table S11. *Conformer label assignment of cage* ***1****,* ***C1-C13***

ID	$conf_{C=O}$	$conf_{C=O}$	degeneracy
**C1**	111111	11-11-11	1
**C2**	011111	01-11-11	6
**C3**	011011	01-10-11	6
**C4**	010111	01-01-11	6
**C5**	010110	01-01-10	6
**C6**	010101	01-01-01	2
**C7**	001111	00-11-11	3
**C8**	000111	00-01-11	12
**C9**	000110	00-01-10	6
**C10**	000101	00-01-01	6
**C11**	000011	00-00-11	3
**C12**	000001	00-00-01	6
**C13**	000000	00-00-00	1

Boltzmann weighting of a population of *i* members was performed using equation se5,

$$f_i = \frac{\exp\left(-\frac{E_i}{RT}\right)}{\sum_i \exp\left(-\frac{E_i}{RT}\right)} \qquad \text{(eq. se5)}$$

where f_i is the fraction contribution of conformer *i*, the *R* is the ideal gas constant, *T* is the temperature, E_i is the energy of *i* relative to the lowest energy member of the population.

Variable Temperature ^{1}H-NMR (VT-NMR) of cage **1** – **Figure S17, S18**

VT-NMR of cage **1** in THF-d_8 indicated cage conformers were still quickly interconverting at –80 °C as only one set of signals was seen.

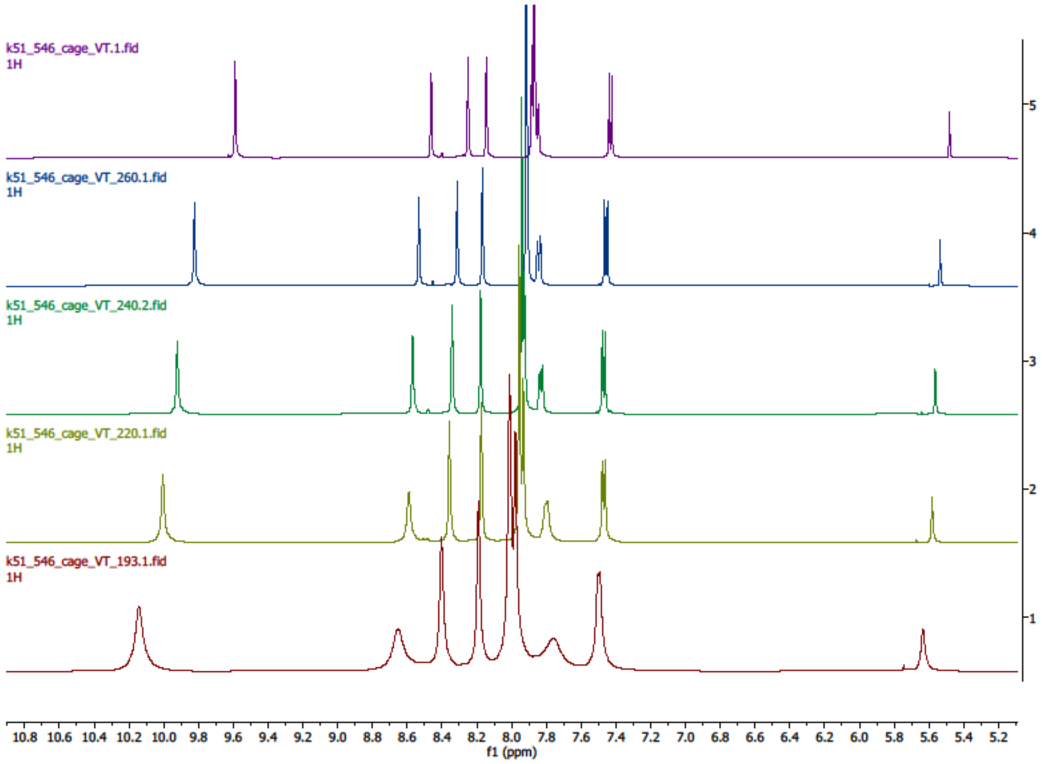

Figure S17. Region of ^{1}H-NMR spectra (THF-d_8) of diacid cage **1** at 298, 260, 240, 220, 193 K.

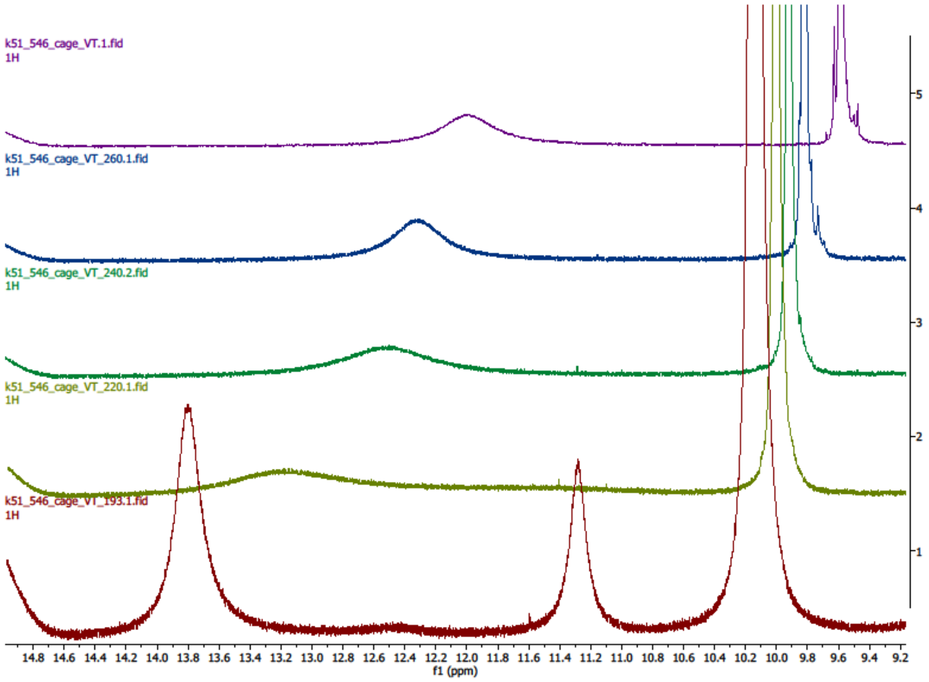

Figure S18. Low field region of ^{1}H-NMR spectra (THF-d_8) of diacid cage **1** at 298, 260, 240, 220, 193 K.

Crystallisation Methodology

Crystallisation was performed using the double vial method, with the outer vial containing methanol (bp = 64.7 °C), which was allowed to slowly diffuse by evaporation into an inner vial containing a solution of THF (bp = 66 °C) containing cage (0.5 mg in ~100-200 µL). The vials were stored in a dark cupboard at ambient temperature (~20 °C) away from light/vibrations. Crystals grew over 1-2 weeks.

Cage **1**: Cage **1** was grown by vapor diffusion of methanol into a solution of cage in THF), which crystallised in the triclinic space group *P*1 with four molecules in the unit cell. Cage **1** crystallised in conformer **C9**. (In one of the two independent cages in the crystal, there is disorder around one of the amide groups, meaning a small contribution to the crystal from conformer **C12**). The "twisted", axially chiral conformation **C9**, which has a single C_2 rotational axis (point group C_2), was present as a pair of (conformational) enantiomers in the crystal of the diacid cage **1**, as indicated by a Flack parameter of 0.48. (The Flack parameter (range: 0–1) is a measure of the (absolute) chirality in a crystal; 0.5 indicates a racemic/twinned mixture).[17]

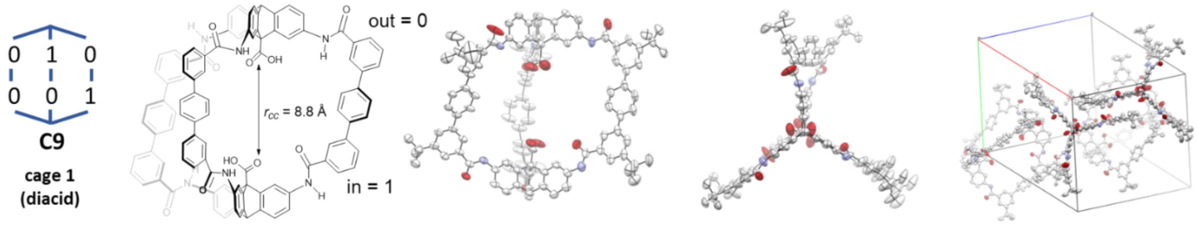

Figure S19. *Crystallography details for cage **1***

Intermolecular hydrogen bonding between cage units for cage **1** is weak, with bond lengths between 2.1-3.0 Å for some of the externally positioned amide carbonyl groups, or externally aligned amide NH groups. We note that cage **1** shows little difference to the previously reported **1e**, suggesting that ester hydrolysis has a negligible effect on the cage conformation.

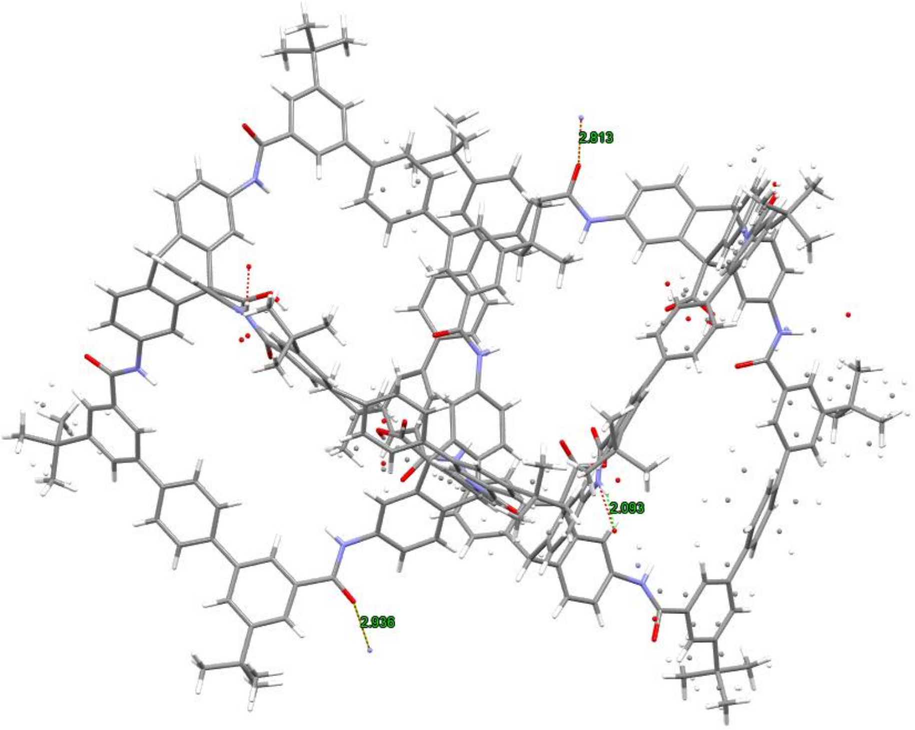

Figure S20. *Intramolecular hydrogen bond contacts for cage **1**.*

Cage **2e**: Single crystals of cage **2e** were grown by vapour diffusion of *n*-pentane into a solution of **2e** in ethyl acetate/THF. Cage **2e** crystallised in the monoclinic space group *P*1*c*1 with two cage molecules in the unit cell. Crystal Data for 2. $C_{182}H_{230}N_{12}O_{31}$, Mr = 3081.77, monoclinic, Pn (No. 7), a = 20.2715(6) Å, b = 42.387(3) Å, c = 25.2850(7) Å, b = 91.715(2)°, a = g = 90°, V = 21716.5(18) Å3, T = 100(2) K, Z = 4, Z' = 2, m(Cu Ka) = 0.516 mm-1, 47982 reflections measured, 13807 unique (Rint = 0.0420) which were used in all calculations. The final wR2 was 0.2739 (all data) and R1 was 0.0898 (I≥2 s(I)).

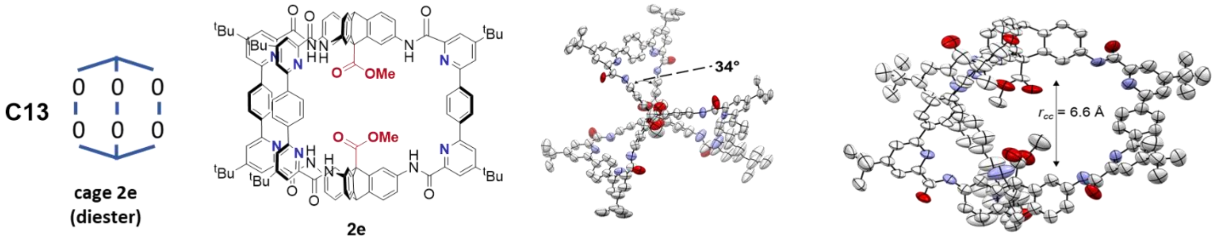

Figure S21. *Crystallography details for cage* ***2e.***

Cage **2e**, of course, has no externally projected NH donors, and so no intramolecular hydrogen bonding is possible. The internal NH→pyridine distances are 2.3 Å.

Cage **4e**: Anthracene cage **4e** crystallized (vapor diffusion of methanol into a solution of cage in THF) in the monoclinic space group *C*2 with four molecules in the unit cell. Anthracene cage **4e** crystallized (**Figure 5iii**) as a pair of (conformational) enantiomers with roughly D_3 symmetry (i.e. with all anthracenyl groups rotated the same way with respect to their two ^{t}-Bu groups) and all six carbonyl groups oriented with oxygen outwards, **C13**. This conformer has a large cap-cap twist angle of ~32°.

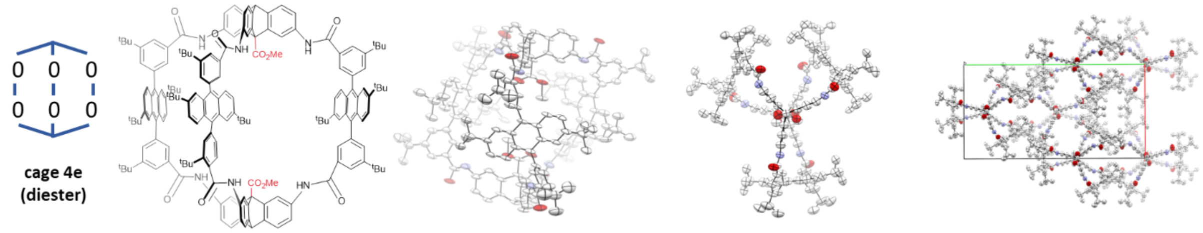

Figure S22. *Crystallography details for cage* ***4e.***

Cage **4e**, of course, has no externally projected NH donors, and so no intramolecular hydrogen bonding is possible.

Full crystallography data is available in separate crystallography files.

DFT study of geometry of cage **1**

Empty cage generation, conformational screening and optimisation

Example cages drawn in Chem3D 21.0.0 (PerkinElmer) were minimised with MM2 molecular mechanics, then MOPAC[18,19] PM7[20] and submitted for conformational screening (simulated annealing) with Grimme's CREST program (Version 2.10.2)[21,22] with xTB (6.4.1-intel-2021a)[23] using implicit THF, and the gfn force-field[24] using the input command:

```
crest input.xyz -T 48 -chrg 0 -g THF --gfnff
```

The resulting conformer xyz file was split into discrete conformers, the single point energies calculated, and the conformers ranked. The cages were evaluated according to amide conformation and the lowest energy molecules for each unique conformer subjected to geometry optimisation at DFT level (see tables below for functionals), and energy, distance and angle parameters extracted. Missing cages were generated manually from existing structures, and optimised.

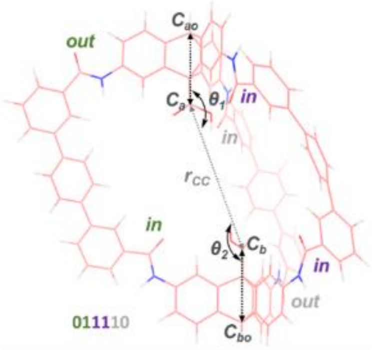

Tables S12-15: *cage **1** parameters generated using different DFT functionals*

Table S12. Parameters of the 13 amide conformations of empty cage **1** (DFT/PBE0/def2-svp-D3BJ (THF)).

ID	*conf$_{C=O}$*	*E_{rel}* (kJ/mol)	*r_{cc}* (Å)	$\bar{\theta}$ (°)	C=O in (%)	Pop (%)
5	010110	0	9.3	167	50	84.8
9	000110	6	8.8	169	33	8.5
3	011011	8	9.6	169	67	4.3
12	000001	10	8.3	172	17	1.1
10	000101	11	8.8	172	33	0.9
13	000000	15	7.8	176	0	0.3
8	000111	18	9.2	170	50	0.1
4	010111	21	9.7	172	67	0.0
11	000011	22	8.7	173	33	0.0
6	010101	23	9.4	178	50	0.0
2	011111	26	10.0	172	83	0.0
7	001111	27	9.6	173	67	0.0
1	111111	28	10.3	177	100	0.0

There exist 13 unique permutations of relative carbonyl geometries, encoded here with six figures (*e.g.* 011110, 1=in, 0=out) given in pairs of carbonyls (top,bottom of a given edge piece). The distance between the two carboxylic acid carbons is denoted by r_{cc}. The angle between the outer triptycene bridgehead carbon C_{ao}, carboxylic acid carbon C_a, and carboxylic acid carbon C_b is denoted θ_1. θ_2, is the analogous angle between C_{bo},C_b,C_a. $\bar{\theta}$ is the mean average of θ_1 and θ_2. Pop is the Boltzmann weighted contribution of this conformer ID to the population calculated using equation se5 (including degeneracy correction).

Table S13. Parameters of the 13 amide conformations of empty cage **1** (DFT/M06-2X/def2-svp (THF)).

ID	*conf$_{C=O}$*	*E_{rel} (kJ/mol)*	*r_{cc} (Å)*	*$\bar{\theta}$ (°)*	**C=O in (%)**	**Pop (%)**
5	010110	0	9.3	168	50	64.1
9	000110	2	8.8	169	33	27.5
3	011011	8	9.6	170	67	2.9
12	000001	7	8.3	172	17	1.3
10	000101	10	8.9	172	33	3.5
13	000000	11	7.9	177	0	0.5
8	000111	14	9.2	170	50	0.1
4	010111	17	9.7	171	67	0.1
11	000011	19	8.7	173	33	0.0
6	010101	17	9.4	177	50	0.0
2	011111	20	10.0	172	83	0.0
7	001111	23	9.7	173	67	0.0
1	111111	24	10.3	178	100	0.0

See Table S12.

Table S14. Parameters of the 13 amide conformations of empty cage **1** (DFT/B3LYP/def2-svp-D3BJ (THF)).

ID	*conf$_{C=O}$*	*E_{rel} (kJ/mol)*	*r_{cc} (Å)*	*$\bar{\theta}$ (°)*	**C=O in (%)**	**Pop (%)**
5	010110	0	9.3	167	50	85.0
9	000110	5	8.8	169	33	9.5
3	011011	9	9.6	168	67	2.5
12	000001	11	8.3	172	17	1.0
10	000101	11	8.8	172	33	0.9
13	000000	7	7.7	177	0	0.9
8	000111	17	9.2	170	50	0.2
4	010111	20	9.7	171	67	0.0
11	000011	22	8.7	173	33	0.0
6	010101	20	9.4	178	50	0.0
2	011111	24	10.0	171	83	0.0
7	001111	26	9.7	173	67	0.0
1	111111	28	10.3	177	100	0.0

See Table S12.

Table S15. Average values of the three DFT methods above, for Boltzman and symmetry corrected population and energy of the 13 amide conformations of empty cage **1**.

ID	*conf$_{C=O}$*	Pop (%)	*E_{rel} (kJ/mol)*
**C5**	010110	78.0	0
**C9**	000110	15.2	4
**C3**	011011	3.2	8
**C12**	000001	1.1	11
**C10**	000101	1.8	9
**C13**	000000	0.5	11
**C8**	000111	0.1	16
**C4**	010111	0.1	19
**C11**	000011	0.0	21
**C6**	010101	0.0	20
**C2**	011111	0.0	23
**C7**	001111	0.0	25
**C1**	111111	0.0	27

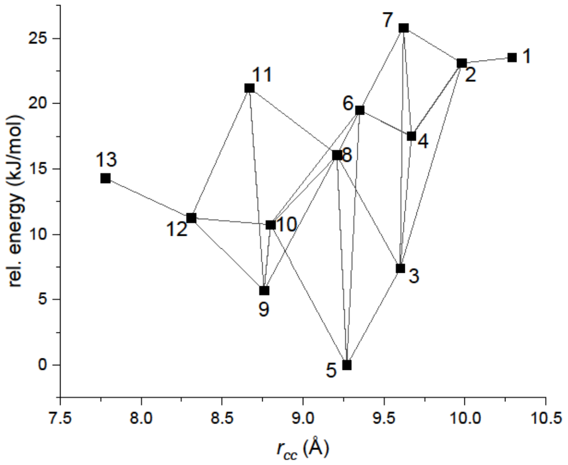

Figure S23. *Connectivity map showing which cage conformers are connected* by a single amide unit rotation, and their cavity heights and relative energies (DFT/PBE0/def2-SVP-D3BJ (THF)).

Molecular Dynamics of cage **1**

The 13 optimised empty conformers of cage **1** [DFT/b3lyp/6-31G(d,p)] were used to generate atom charges for the RESP method of charge generation. The charges were used to generate topologies suitable for use in GROMACS/2020.4-foss-2020a.

Gaussian route card for RESP charge generation:

```
# hf/6-31g* pop=mk iop(6/33=2,6/41=10,6/42=17) scf=tight
```

AMBER/antechamber commandline commands for generating topologies from the resulting Gaussian output files (GaussianOutput.log) (MAC OS 10.14.6):

- antechamber -fi gout -fo mol2 -c resp -i GaussianOutput.log -o res.mol2 -at amber -pl 15
- parmchk2 -i res.mol2 -f mol2 -o cage.frcmod
- tleap -f oldff/leaprc.ff99SB
- source leaprc.gaff
- MOL = loadmol2 res.mol2
- loadamberparams cage.frcmod
- saveoff MOL mol.lib
- saveamberparm MOL cage.prmtop cage.inpcrd
- saveamberparm MOL cage.prmtop cage.rst7
- saveamberparm MOL cage.top cage.rst7
- quit

Then using python:

- python
- import parmed as pmd
- amber = pmd.load_file('/…/ cage.prmtop', '/…/ cage.inpcrd')
- amber.save('/…/gromacs.top')
- amber.save('/…/gromacs.gro')
- quit()

Then using GROMACS:

(THF parameters (109-99-9-liq.pdb) obtained here: http://virtualchemistry.org/ff.php)

- gmx editconf -f 109-99-9-liq.pdb -o THF_1box.gro -d 0.26 -bt cubic
    - delete all molecules except one → THF_single.gro
- gmx editconf -f Cage.gro -o CageBox.gro -c -d 1.0 -bt cubic
- gmx insert-molecules -ci THF_single.gro -f CageBox.gro -nmol 2300 -try 2 -o cage_in_THF

more solvent molecules can be added iteratively:

- *gmx insert-molecules -ci THF_single.gro -f cage_in_THF.gro -nmol 2300 -try 2 -o cage_in_THF*

Perform GROMACs admin to avoid errors:

- manually edited the file cage_in_THF.gro to include 496THF and 6617 atoms (for consistency between cage conformers)
- edited MOL label to THF in various files:
    - THF.itp (change residue name near top)
    - Cage_THF.gro (edit 2MOL, 3MOL etc to 2THF, 3THF, etc) (e.g. (find/replace 1MOL ->>> MMMM, then MOL —> THF, then MMMM —> 1MOL)

- added the THF atom parameters to the Cage_in_THF topology file and removed them from the THF.itp file

```
c3    c3    0.0000  0.0000  A   3.39967e-01  4.57730e-01
h1    h1    0.0000  0.0000  A   2.47135e-01  6.56888e-02
hc    hc    0.0000  0.0000  A   2.64953e-01  6.56888e-02
os    os    0.0000  0.0000  A   3.00001e-01  7.11280e-01
```

- manually edited Cage.top to Cage_THF.top by altering THF molecules to 496 at bottom

```
MOL        1
THF      496
```

- deleted the [defaults] section from THF.itp file

➢ check parameter files (included in supporting files)
  - minim.mdp file
  - THF.itp file
  - nvt.mdp
  - npt.mdp
  - md.mdp

RUN GROMACS EQUILIBRATION STEPS

➢ gmx grompp -f minim.mdp -c cage_in_THF.gro -p Cage_THF.top -o em.tpr
➢ gmx mdrun -v -deffnm em

After initial minimisation, add position restraints onto the cage to stop unwanted isomerisation during equilibration:

➢ gmx make_ndx -f em.tpr -o ndx.ndx
➢ q
➢ gmx genrestr -f cage_in_THF.gro -n ndx.ndx -o cage_in_THF.itp
  - Chose MOL (2) group to restrain only the cage, and not the THF solvent.
  - Note: if this command doesn't offer a second group, the MOL and THF are not correctly distinguished in all the files.
  - May need to also: manually edit cage_in_THF.itp to have the right number of atom restraints (182 for this cage) by deleting the extras.
  - Edit bottom of topology file (Cage_THF.top)

```
; Include Position restraint file
#ifdef POSRES
#include "cage_in_THF.itp"
#endif

#include “THF.itp"

[ system ]
; Name
Generic title

[ molecules ]
; Compound       #mols
MOL                1
THF              496
```

Equilibration steps:

See included parameter files for details.

➢ gmx grompp -f nvt.mdp -n ndx.ndx -c em.gro -r em.gro -p Cage_THF.top -o nvt.tpr

- gmx mdrun -deffnm nvt
- gmx grompp -f npt.mdp -c nvt.gro -r nvt.gro -t nvt.cpt -p Cage_THF.top -o npt.tpr
- gmx mdrun -deffnm npt
- gmx grompp -f md.mdp -c npt.gro -t npt.cpt -p Cage_THF.top -o md_0_1.tpr
- gmx mdrun -deffnm md_0_1

Alternatively, the final production md run was performed on a cluster using mpi threads (GROMACS/2020.4-foss-2020a).

- gmx_mpi grompp -f md.mdp -o md_0_1.tpr -c npt.gro -t npt.cpt -p Cage_THF.top
- gmx_mpi mdrun -deffnm md_0_1 -npme 16

The BASH script included:

```
#SBATCH --nodes=1
#SBATCH --ntasks-per-node=48
#SBATCH --mem-per-cpu=7G
```

NOTES: The cage topology files were inspected. Atom charges generated using RESP were consistent by symmetry within a cage structure, but showed slight variations between different starting cage conformations. These charge differences (absolute average difference = ±0.03) were not considered to disrupt the accuracy of the conformational searching experiment performed. However, it reinforced the decision to calculate a separate topology for each initial (amide) conformer.

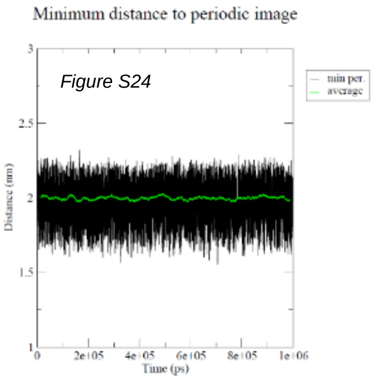

Figure S24

The 13 topologies were each placed in a cuboid box and 496 molecules of THF added; the resulting boxes were approximately the same size, which was ~4.3 nm^3. The average distance between the cage molecule and itself in a periodic system was measured at 2.0 nm (with a minimum distance as measured by the GROMACS mindist function as 1.55 nm), which is sufficient that the cage is not "seeing itself" in a way that would influence calculation.

Each solvated cage conformer was equilibrated using typical energy minimisation (emtol = 50 kJ/mol/nm, 10^{10} steps), volume ($5*10^4$ steps, dt = 0.002 ns, cage restrained*, V-rescale (modified Berendsen thermostat)), and pressure equilibration ($5*10^5$ steps, dt = 0.001 ns, cage restrained*, Parrinello-Rahman coupling). [*The cage geometry was restrained in cases where equilibration altered the conformer of the cage away from the desired starting conformer (**C10, 3, 7, 2, 4**)]. Each cage was subjected to 1 µs production run times (10^{10} steps, dt = 0.001 ns, V-rescale temp coupling, Parrinello-Rahman pressure coupling, 300 K).

Using the GROMACS xtc output file, the conformer geometry was sampled every 5 ps by measuring the distance between the amide carbonyl oxygen atoms and the external ortho-triptycene proton (H^7) using a python script (xvg.process.py; included in supporting files) to analyse pairwise atom data generated using the GROMACS distance tool. A threshold of 0.36 nm was used to trigger report of a new conformer by amide rotation (< 0.36 nm means carbonyl is out). The orientations were converted into normalised "Cage ID" values which were sorted alphabetically (first within each pair of edge carbonyls, retaining top/bottom information across the three edges; then reordering the edges alphabetically, which is possible due to the cage symmetry) to ensure cages were symmetry normalised for comparison. (see: demonstration of cage **1** conformer permutations – **C1-C13**).

Each production data run took ~2.5 days (MPI) (11533259.988 core seconds) using 48 cores with 7 Gb per CPU, and produced ~5 Gb data.

This provided for each conformer a table of orientations over the microsecond production run.

Table S16. *Example of cage conformational data scraping from MD file.*

ID	time (ns)	O_{amide} - H_{trip} distance (nm) 1a	1b	2a	2b	3a	3b	C=O orientation 1a	1b	2a	2b	3a	3b	new?	Cage ID
0	0	0.457	0.454	0.231	0.242	0.237	0.238	1	1	0	0	0	0	1	000011
1	5	0.458	0.460	0.220	0.236	0.242	0.235	1	1	0	0	0	0	0	000011
2	10	0.470	0.431	0.237	0.249	0.237	0.251	1	1	0	0	0	0	0	000011
3	15	0.446	0.473	0.254	0.229	0.262	0.252	1	1	0	0	0	0	0	000011
4	20	0.453	0.470	0.241	0.225	0.246	0.238	1	1	0	0	0	0	0	000011
…	…	…	…	…	…	…	…	…	…	…	…	…	…	…	…
33	165	0.418	0.436	0.228	0.247	0.253	0.236	1	1	0	0	0	0	0	000011
34	170	0.473	0.444	0.242	0.244	0.236	0.236	1	1	0	0	0	0	0	000011
35	175	0.242	0.487	0.254	0.236	0.250	0.236	0	1	0	0	0	0	1	000001
36	180	0.219	0.463	0.238	0.25	0.228	0.240	0	1	0	0	0	0	0	000001
37	185	0.251	0.439	0.231	0.249	0.248	0.248	0	1	0	0	0	0	0	000001
38	190	0.242	0.455	0.224	0.225	0.254	0.252	0	1	0	0	0	0	0	000001
39	195	0.245	0.455	0.225	0.249	0.245	0.262	0	1	0	0	0	0	0	000001
…	…	…	…	…	…	…	…	…	…	…	…	…	…	…	…

Each change in conformation was noted, and checked manually to ensure a genuine conformation change had occurred (there are occasional moments during partial twisting where the threshold is exceeded momentarily but the amide does not complete rotation.) All transitions from the 13 runs are summarised in the following tables (one table per run, each table starting at a different conformer). Shown in Table S17 are the frames/time in ps at which changes were observed, which cage was sampled, and the frequency/population of that cage during the entire simulation.

Table S17. *Molecular dynamics: 13 x 1 µs simulations of cage* ***1*** *showing transitions between conformers.*

frame	time (ps)	cage ID	Freq	pop (%)
0	0	111111	527	0.3
527	2635	011111	49	0
576	2880	011011	330	0.2
906	4530	010110	199095	99.5

conformer 13 [111111]

frame	time (ps)	cage ID	Freq	pop (%)
0	0	000101	272	0.1
272	1360	010110	199728	99.9

conformer 10 [000101]

frame	time (ps)	cage ID	Freq	pop (%)
0	0	000110	1527	0.8
1527	7635	010110	198473	99.2

conformer 9 [000110]

frame	time (ps)	cage ID	Freq	pop (%)
0	0	010110	197519	98.8
14987	74935	000110	2471	1.2
16409	82045	010110		
41332	206660	000110		
41780	208900	010110		
41781	208905	000110		
42380	211900	010110		
116771	583855	011011	11	0
116782	583910	010110		
141502	707510	000110		
141503	707515	010110		

conformer 5 [010110]

frame	time (ps)	cage ID	Freq	pop (%)
0	0	011011	31	0
31	155	010110	199970	100

conformer 3 [011011]

frame	time (ps)	cage ID	Freq	pop (%)
0	0	000001	3549	1.8
676	3380	000110	3670	1.8
677	3385	000001		
3550	17750	000110		
6324	31620	010110	192782	96.4
6326	31630	000110		
7219	36095	010110		
27327	136635	000110		
27328	136640	010110		
63397	316985	000110		
63398	316990	010110		

conformer 12 [000001]

frame	time (ps)	cage ID	Freq	pop (%)
0	0	000011	35	0.0
35	175	000001	1568	0.8
1603	8015	000110	892	0.4
1824	9120	010110	197505	98.8
116921	584605	000110		

conformer 11 [000011]

frame	time (ps)	cage ID	Freq	pop (%)
0	0	000000	18014	9
18014	90070	000001	26746	13.4
44761	223805	000110	22015	11
65673	328365	010110	133225	66.6
90750	453750	000110		
91810	459050	010110		

122584	612920	000110
122627	613135	010110

conformer 13 [000000]

frame	time (ps)	cage ID	Freq	pop (%)
0	0	000111	116	0.1
116	580	000110	2119	1.1
804	4020	010110	197764	98.9
2932	14660	000101	2	0
2934	14670	010110		
6074	30370	000110		
6337	31685	010110		
47921	239605	000110		
49088	245440	010110		

conformer 8 [000111]

frame	time (ps)	cage ID	Freq	pop (%)
0	0	001111	105	0.1
105	525	011111	34	0
139	695	010111	45	0
183	915	010110	199816	99.9
55480	277400	011011	1	0
55481	277405	010110		
87101	435505	010111		
87102	435510	010110		

conformer 7 [001111]

from conf_127 (001111)

frame	time (ps)	cage ID	Freq	pop (%)
0	0	011111	148	0.1
148	740	011011	501	0.3
649	3245	010110	199246	99.6
159903	799515	000110	106	0.1
160009	800045	010110		

conformer 2 [011111]

frame	time (ps)	cage ID	Freq	pop (%)
0	0	010101	1050	0.5
1050	5250	000101	17	0
1067	5335	010110	196982	98.5
145249	726245	000110	805	0.4
145277	726385	000001	1147	0.6
146424	732120	000110		
146889	734445	010110		
187718	938590	000110		
188030	940150	010110		

conformer 6 [010101]

frame	time (ps)	cage ID	Freq	pop (%)
0	0	010111	58	0
58	290	010110	198925	99.5
54362	271810	000110	1018	0.5
55380	276900	010110		

conformer 4 [010111]

This provided a total of 13 microseconds. The frame frequency of each conformer over the 13 microseconds was collated and analysed to provide the overall population statistics for the difference cage conformers (Table S18).

Table S18. *Molecular Dynamics populations. Populations (% time the cage was in this amide conformation) of different cage conformations (**C1-C13**, for cage **1**) observed during 13 microseconds of molecular dynamics (298 K, THF), when starting equally from each possible conformer.*

conformer ID	cage ID	frame freq	pop (%)
5	010110	2511030	96.578
9	000110	34623	1.332
12	000001	33010	1.270
13	000000	18014	0.693
6	010101	1050	0.040
3	011011	874	0.034
1	111111	527	0.020
10	000101	291	0.011
2	011111	231	0.009
8	000111	116	0.004
7	001111	105	0.004
4	010111	103	0.004
11	000011	35	0.001

frames are 5 ps/frame

GENERAL OBSERVATIONS

- 68 transitions observed; 35 from carbonyl in to carbonyl out; 33 from carbonyl out to carbonyl in.
- That is: 5.23 transitions per cage molecule per μs^{-1}.

Input commands for processing the output data

Using GROMACS: After converting the output trajectory with trjconv, pairs of atoms are defined in an index file for distance calculation. The distance function then returns the distances between pairs of atoms for the desired timestep.

- gmx trjconv -f md_0_1.xtc -s md_0_1.gro -n ndx.ndx -pbc mol -ur compact -center -o md_center.gro
- gmx make_ndx -f md_0_1.gro -o ndx_pairs.ndx
    - a O7 H56
    - a O4 H12
    - a O8 H58
    - a O5 H14
    - a O6 H54
    - a O3 H10

    - del0
    - del0
    - del0
    - del0
- q
- gmx distance -f md_0_1.xtc -s md_0_1.gro -n ndx_pairs.ndx -oav carbonyls.xvg
    - (selected all pairs)
- ctrl+D

The meta data was removed manually from the start of the carbonyls.xvg file to leave just the numeric table of distances between the pairs of define atoms.

Then in python (using the script xvg.process.py in the supporting files):

- python xvg.process.py

identifies all amide unit rotations, to give the output data shown above (defining carbonyl orientation), ready for manual checking for false positives at the output time values.

Cage Conformer Molecular Dynamics Transition Pathways

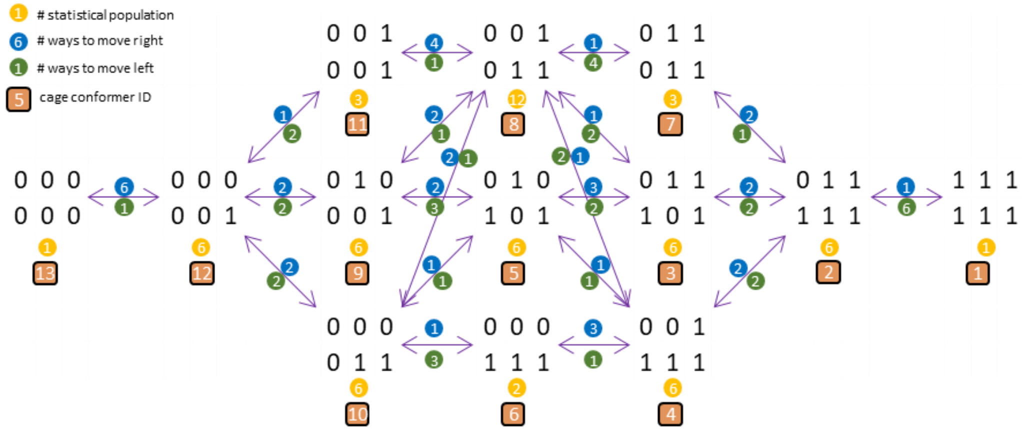

Figure S25. Depiction of the stepwise interconversion network between cage conformers assuming only single amide rotations. Also shown are the cage conformer labels, the relative statistical population of each conformer (i.e. permutations of 0 (carbonyl out) or 1 (carbonyl in) that access an indistinguishable (degenerate) isomer), and how many of the six carbonyls can rotate to traverse a given pathway, “right or left”.

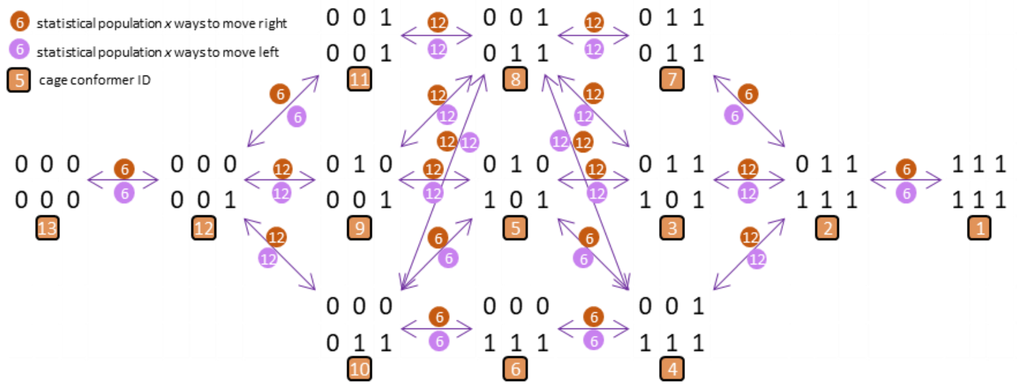

Figure S26. Depiction of the stepwise interconversion network between cage conformers and statistical-population-normalised relative transition probability.

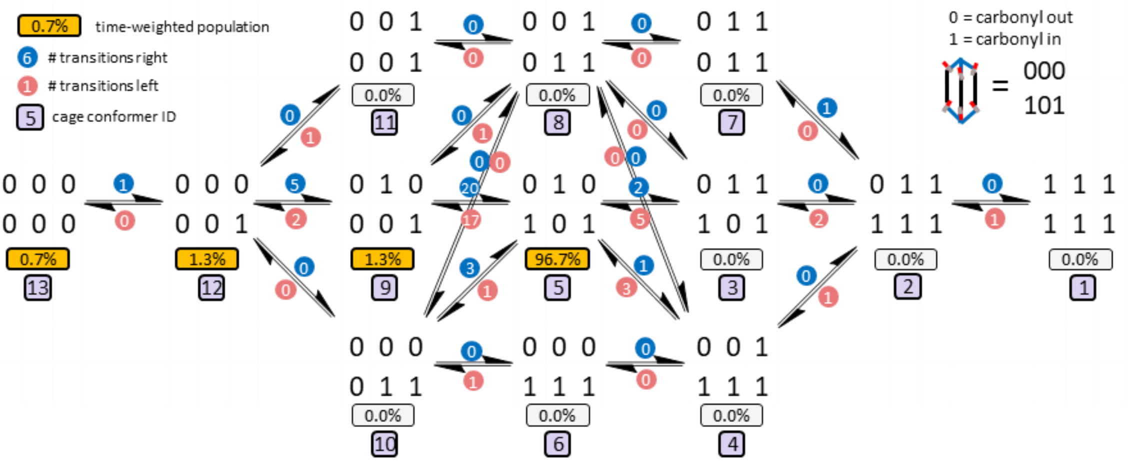

Figure S27. Depiction of number of transitions between conformers observed in the 13 µs molecular dynamics run, and the populations of each cage conformer observed.

Table S19. Number of transitions between conformers [from (row) to (col)] observed during the 13 µs MD simulations of cage **1**.

		to												
	conformer ID	**1**	**2**	**3**	**4**	**5**	**6**	**7**	**8**	**9**	**10**	**11**	**12**	**13**
from	**1**	0	1	0	0	0	0	0	0	0	0	0	0	0
	**2**	0	0	2	1	0	0	0	0	0	0	0	0	0
	**3**	0	0	0	0	5	0	0	0	0	0	0	0	0
	**4**	0	0	0	0	3	0	0	0	0	0	0	0	0
	**5**	0	0	2	1	0	0	0	0	17	1	0	0	0
	**6**	0	0	0	0	0	0	0	0	0	1	0	0	0
	**7**	0	1	0	0	0	0	0	0	0	0	0	0	0
	**8**	0	0	0	0	0	0	0	0	1	0	0	0	0
	**9**	0	0	0	0	20	0	0	0	0	0	0	2	0
	**10**	0	0	0	0	3	0	0	0	0	0	0	0	0
	**11**	0	0	0	0	0	0	0	0	0	0	0	1	0
	**12**	0	0	0	0	0	0	0	0	5	0	0	0	0
	**13**	0	0	0	0	0	0	0	0	0	0	0	1	0

NOE data – **Figures S28-33**

NOE data can give an indication of average distance between two protons in a molecule. If the cages had a preference for the amide carbonyl oxygens to be oriented outwards, the NH proton signal might be expected to exchange more magnetisation with the inner protons, H^5 and H^{13}, as compared to the corresponding external protons at the same distance, H^7 and H^{15}, respectively.

NOE data for cage 1e

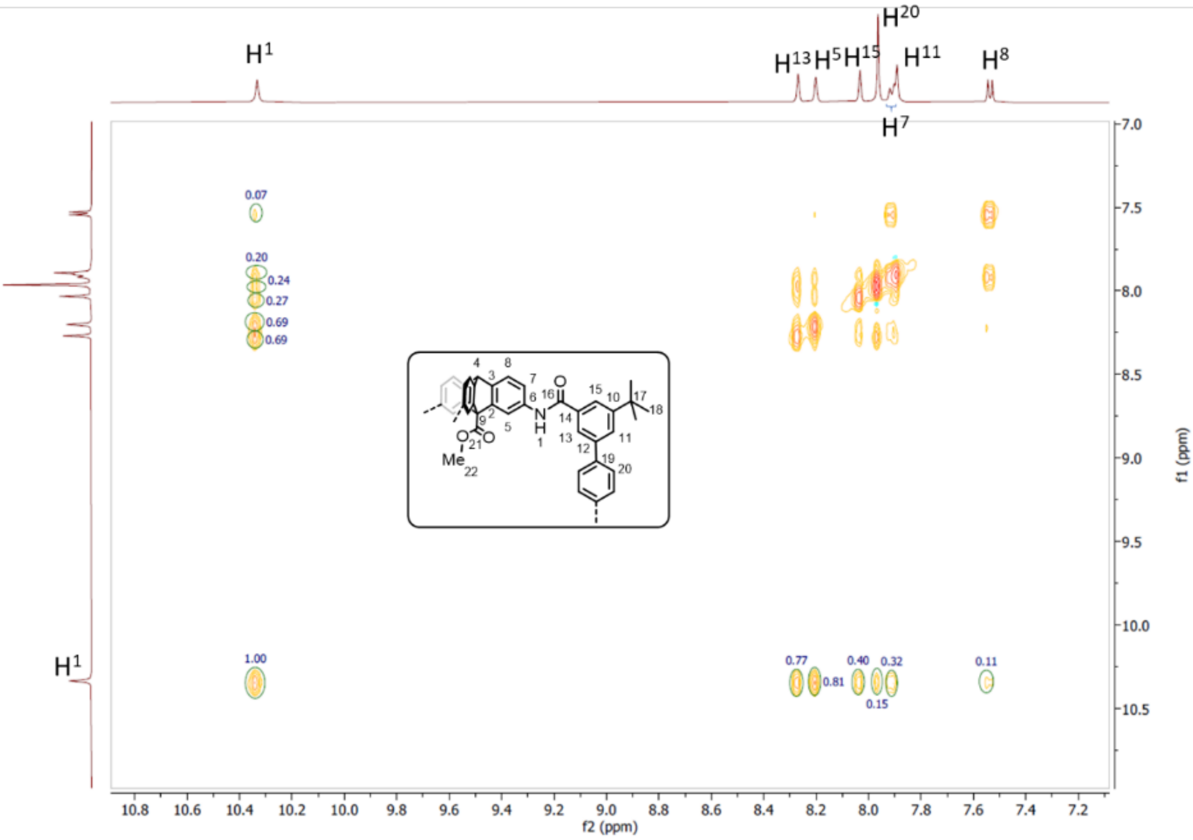

Figure S28. *^{1}H-NOE NMR experiments (DMSO-d_6) of cage **1e** show NOE values of: H^1-H^{13}:H^1-H^{15} (0.77:0.40) and H^1-H^5:H^1-H^7 (0.81:0.32). This equates to a ~2:1 preference for NOE transfer from the NH to inner protons, which could indicate the population has mostly carbonyls oriented outwards.*

NOE data for cage 1_{HH}

For reference, the NOE data for the separately reported analogue of cage **1** without internal carboxylate groups.

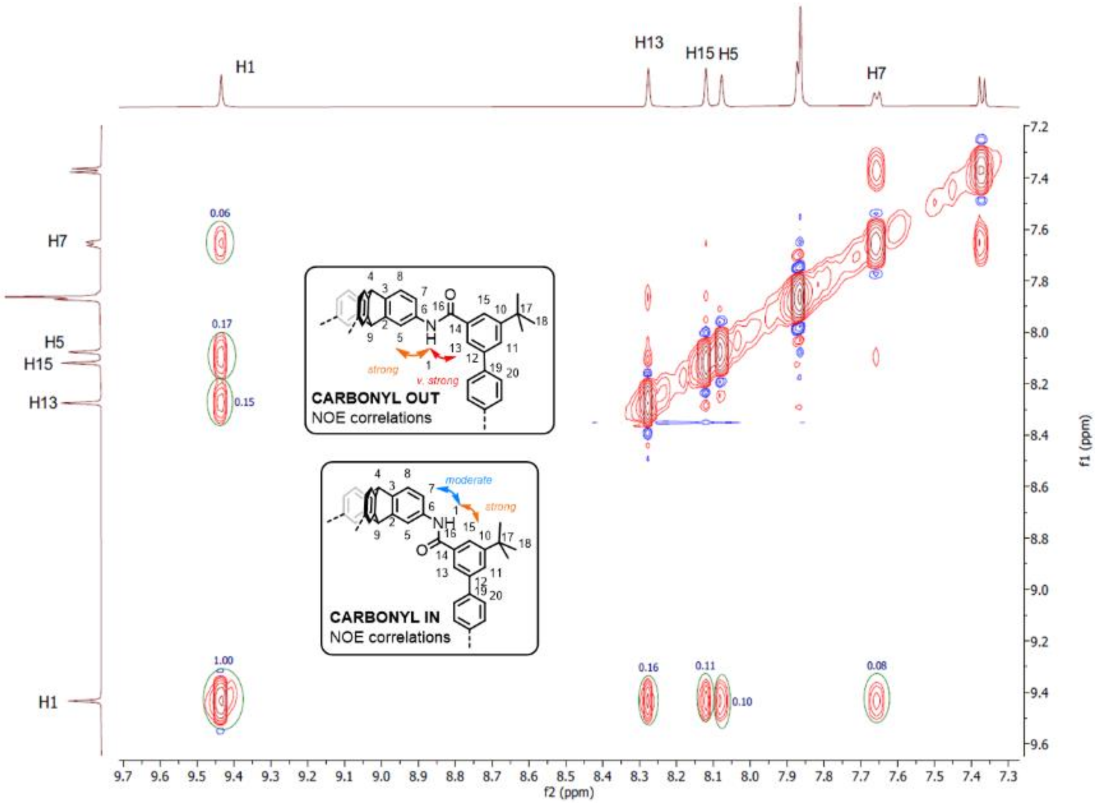

Figure S29. *^{1}H-NOE NMR experiments (THF-d_8) cage* ***1_{HH}*** *show NOE values of: H^1-H^{13}:H^1-H^{15} (0.16:0.11) and H^1-H^5:H^1-H^7 (0.10:0.08). This equates to a ~4:3 preference for NOE transfer from the NH to inner protons, which could indicate the population has mostly carbonyls oriented outwards.*

NOE data for cage 1

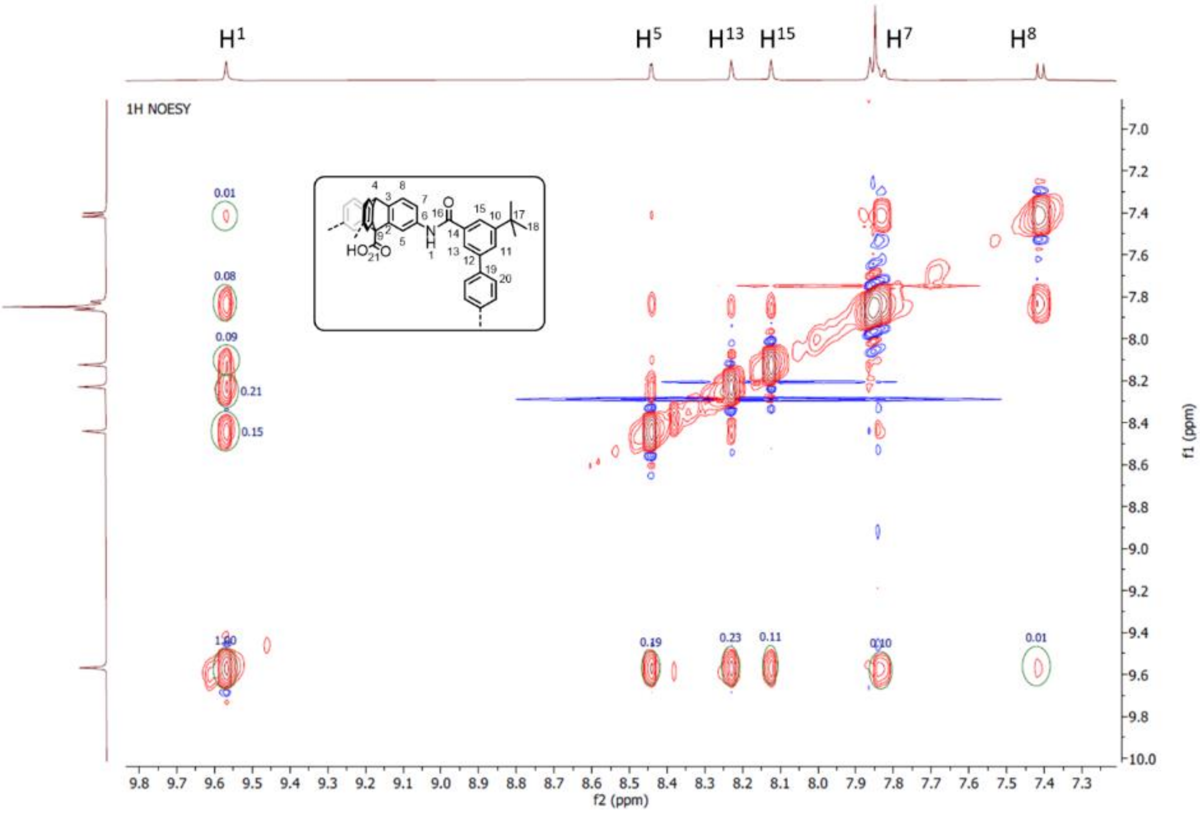

Figure S30. *^{1}H-NOE NMR data (THF-d$_8$) cage* ***1*** *show NOE values of: H^1-H^{13}:H^1-H^{15} (0.23:0.11) and H^1-H^5:H^1-H^7 (0.19:0.10). This equates to a ~2:1 preference for NOE transfer from the NH to inner protons, which could indicate the population has mostly carbonyls oriented outwards.*

NOE data for cage 2_{HH}

For reference, the NOE data for the separately reported analogue of hexapyridine cage **2** without internal carboxylate groups.

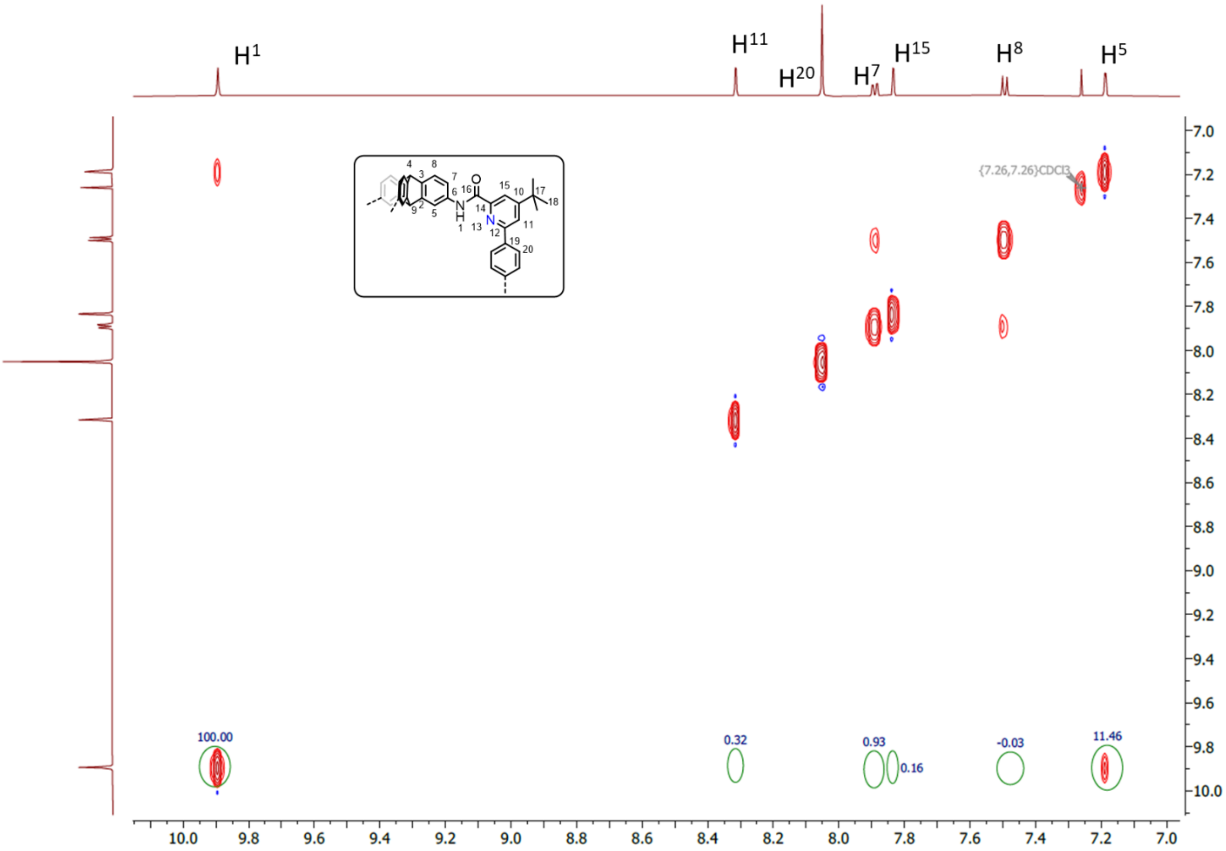

Figure S31. *^{1}H-NOE NMR data (THF-d_8) cage* $\mathbf{2_{HH}}$ *shows NOE values of H^1-H^5:H^1-H^7 (11.46:0.93). This equates to a ~92:8 preference for NOE transfer from the NH to inner protons, which strongly indicates the population has mostly carbonyls oriented outwards.*

NOE data for cage 3e

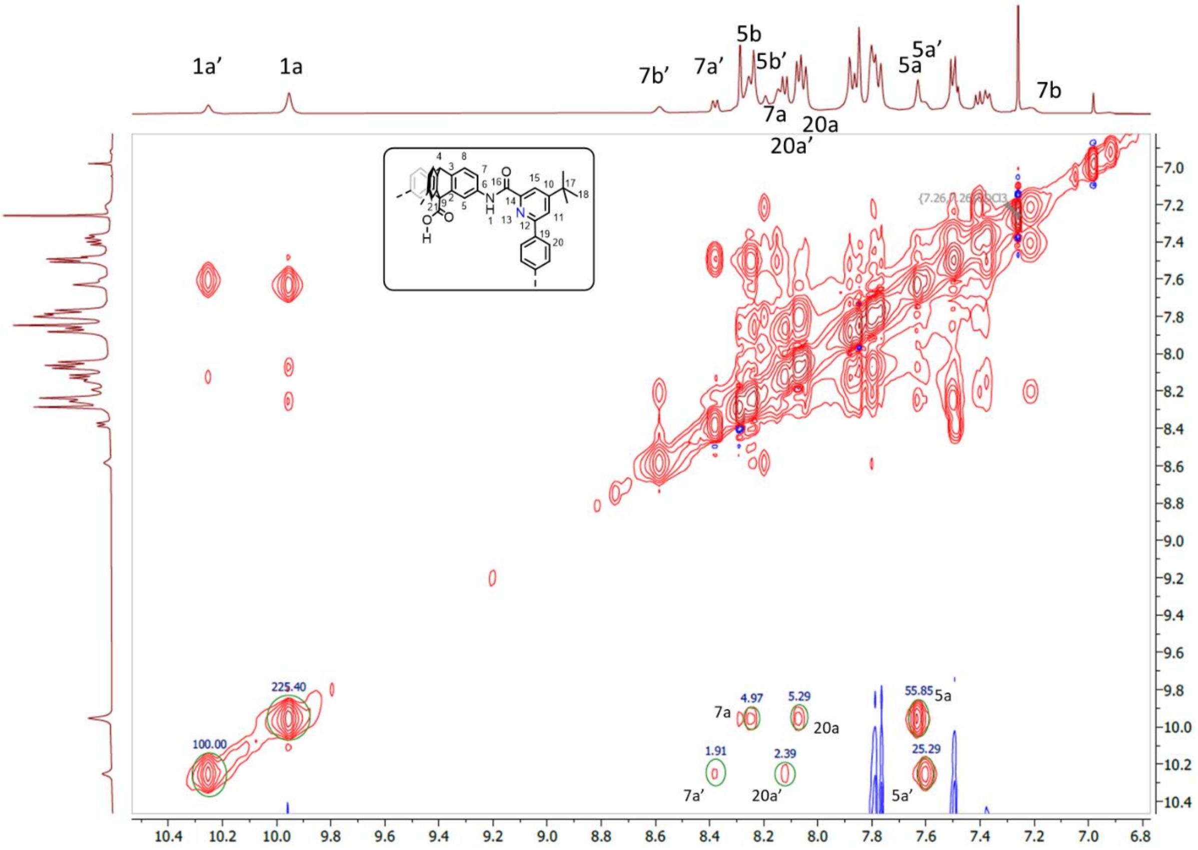

Figure S32. *^{1}H-NOE NMR data (THF-d_8) cage **3e** shows NOE values of H^{1a}-H^{5a}:H^{1a}-H^{7a} (55.85:4.97). This equates to a ~92:8 preference for NOE transfer from the NH to inner protons. Additionally, the ratio of $H^{1a'}$-$H^{5a'}$:$H^{1a'}$-$H^{7a'}$ (25.29:1.91) gives 93:7.These strongly support the assignment of a majority population of conformer **C5**. The non-pyridyl amide NH groups are not sufficiently resolved for analysis.*

NOE data for cage 4

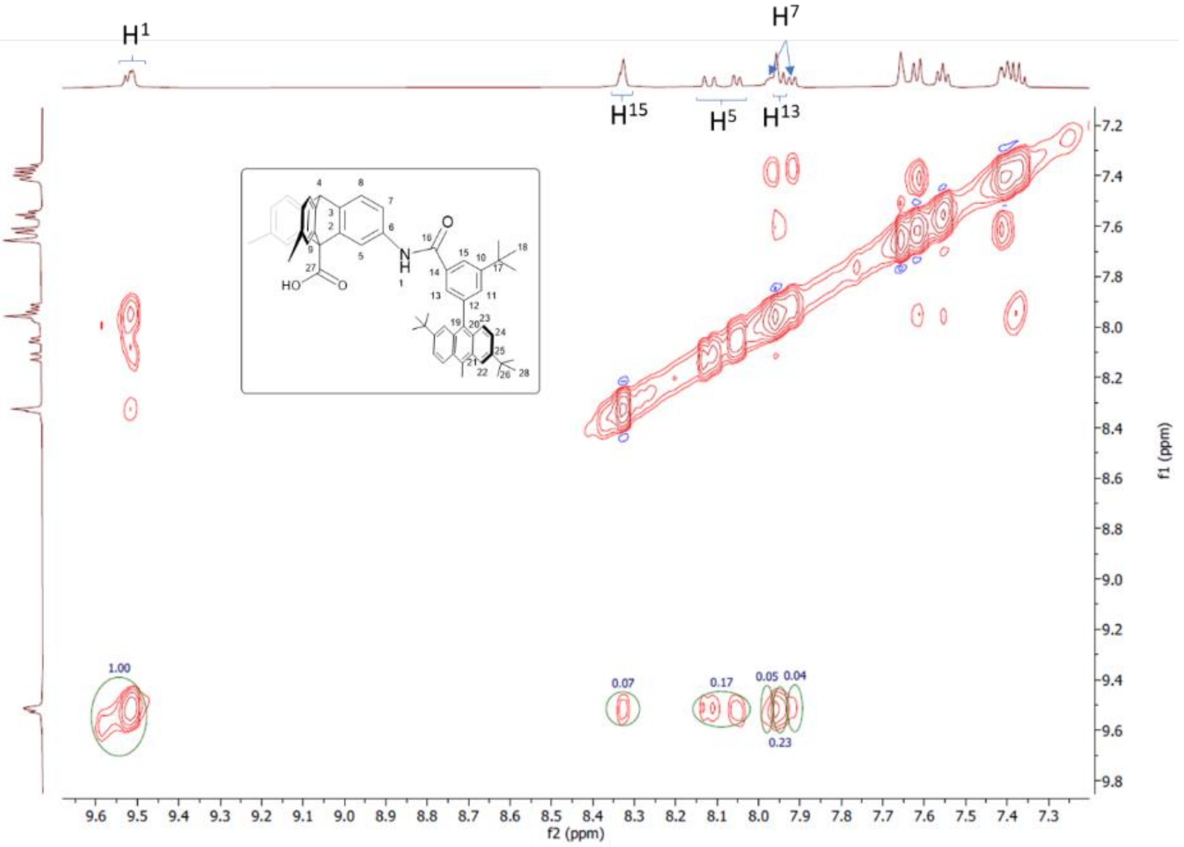

Figure S33. *^{1}H-NOE NMR data (THF-d_8) cage **4** shows NOE values of H^1-H^{13}:H^1-H^{15} (0.23:0.07) and H^1-H^5:H^1-H^7 (0.17:0.09). This equates to a ~5:2 preference for NOE transfer from the NH to inner protons, which could indicate the population has mostly carbonyls oriented outwards in the solution state.*

References for supporting information

1. Richards, A. (2015). University of Oxford Advanced Research Computing. Univ. Oxford Adv. Res. Comput., *783*, 1–4. https://doi.org/10.5281/ZENODO.22558

2. O'Boyle, N. M., Banck, M., James, C. A., Morley, C., Vandermeersch, T., & Hutchison, G. R. (2011). Open Babel: An Open chemical toolbox. J. Cheminform., *3*, 1–14. https://doi.org/10.1186/1758-2946-3-33

3. Young, T. A., Silcock, J. J., Sterling, A. J., & Duarte, F. (2021). autodE: Automated Calculation of Reaction Energy Profiles— Application to Organic and Organometallic Reactions. Angew. Chem. Int. Ed., *60*, 4266–4274. https://doi.org/10.1002/ANIE.202011941

4. Samanta, R. C., & Yamamoto, H. (2015). Selective Halogenation Using an Aniline Catalyst. Chem. Eur. J., *21*, 11976–11979. https://doi.org/10.1002/chem.201502234

5. Xiong, X., Tan, F., & Yeung, Y. Y. (2017). Zwitterionic-Salt-Catalyzed Site-Selective Monobromination of Arenes. Org. Lett., *19*, 4243–4246. https://doi.org/10.1021/acs.orglett.7b01899

6. Friedman, L., & Logullo, F. M. (1963). Benzynes via aprotic diazotization of anthranilic acids: A convenient synthesis of triptycene and derivatives. J. Am. Chem. Soc., *85*, 1549. https://doi.org/10.1021/ja00893a045

7. Granda, J. M., Grabowski, J., & Jurczak, J. (2015). Synthesis, Structure, and Complexation Properties of a C3-Symmetrical Triptycene-Based Anion Receptor: Selectivity for Dihydrogen Phosphate. Org. Lett., *17*, 5882–5885. https://doi.org/10.1021/acs.orglett.5b03066

8. Quast, H., Eckert, P., & Seiferling, B. (1985). Brückenkopfazide, 5. Photolyse von 9-Azidotriptycenen. – Addition von Methanol oder Wasser an extrem gespannte intermediäre Brückenkopfimine. Liebigs Ann. Der Chemie, *1985*, 696–707. https://doi.org/10.1002/JLAC.198519850406

9. Yoon, I., Suh, S. E., Barros, S. A., & Chenoweth, D. M. (2016). Synthesis of 9-Substituted Triptycene Building Blocks for Solid-Phase Diversification and Nucleic Acid Junction Targeting. Org. Lett., *18*, 1096–1099. https://doi.org/10.1021/acs.orglett.6b00169

10. Andrews, K. G., & Christensen, K. E. (2023). Access to Amide-linked Organic Cages by in situ Trapping of Metastable Imine Assemblies: Solution Phase Bisamine Recognition. Chem. – A Eur. J., e202300063. https://doi.org/10.1002/chem.202300063

11. Buonomo, J. A., Everson, D. A., & Weix, D. J. (2013). Substituted 2,2′-Bipyridines by Nickel Catalysis: 4,4′-Di-tert-butyl-2,2′-bipyridine. Synthesis (Stuttg)., *45*, 3099–3102. https://doi.org/10.1055/S-0033-1338520

12. Wang, J., Wan, W., Jiang, H., Gao, Y., Jiang, X., Lin, H., Zhao, W., & Hao, J. (2010). C-9 fluorenyl substituted anthracenes: A promising new family of blue luminescent materials. Org. Lett., *12*, 3874–3877. https://doi.org/10.1021/ol101553a

13. Bringmann, G., Mortimer, A. J. P., Keller, P. A., Gresser, M. J., Garner, J., & Breuning, M. (2005). Atroposelective Synthesis of Axially Chiral Biaryl Compounds. Angew. Chem. Int. Ed., *44*, 5384–5427. https://doi.org/10.1002/ANIE.200462661

14. Aitken, R. A., Smith, M. H., & Wilson, H. S. (2016). Variable temperature 1H and 13C NMR study of restricted rotation in N,N-bis(2-hydroxyethyl)acetamide. J. Mol. Struct., *1113*, 171–173. https://doi.org/10.1016/j.molstruc.2016.02.030

15. Nozawa-Kumada, K., Iwakawa, Y., Onuma, S., Shigeno, M., & Kondo, Y. (2020). NaH-mediated direct C–H arylation in the presence of 1,10-phenanthroline. Chem. Commun., *56*, 7773–7776. https://doi.org/10.1039/D0CC00730G

16. Thordarson, P. (2011). Determining association constants from titration experiments in

supramolecular chemistry. Chem. Soc. Rev., *40*, 1305–1323. https://doi.org/10.1039/C0CS00062K

17. Flack, H. D. (1983). On enantiomorph-polarity estimation. Acta Crystallogr. Sect. A, *39*, 876–881. https://doi.org/10.1107/S0108767383001762

18. Stewart, J. J. P. (1996). Application of localized molecular orbitals to the solution of semiempirical self-consistent field equations. Int. J. Quantum Chem., *58*, 133–146. https://doi.org/10.1002/(SICI)1097-461X(1996)58:2<133::AID-QUA2>3.0.CO;2-Z

19. Stewart, J. J. P. (2016). *MOPAC2016*. Stewart Computational Chemistry. http://openmopac.net

20. Stewart, J. J. P. (2013). Optimization of parameters for semiempirical methods VI: more modifications to the NDDO approximations and re-optimization of parameters. J. Mol. Model., *19*, 1–32. https://doi.org/10.1007/s00894-012-1667-x

21. Pracht, P., Bohle, F., & Grimme, S. (2020). Automated exploration of the low-energy chemical space with fast quantum chemical methods. Phys. Chem. Chem. Phys., *22*, 7169–7192. https://doi.org/10.1039/c9cp06869d

22. Grimme, S. (2019). Exploration of Chemical Compound, Conformer, and Reaction Space with Meta-Dynamics Simulations Based on Tight-Binding Quantum Chemical Calculations. J. Chem. Theory Comput., *15*, 2847–2862. https://doi.org/10.1021/acs.jctc.9b00143

23. Bannwarth, C., Caldeweyher, E., Ehlert, S., Hansen, A., Pracht, P., Seibert, J., Spicher, S., & Grimme, S. (2021). Extended tight-binding quantum chemistry methods. Wiley Interdiscip. Rev. Comput. Mol. Sci., *11*, e1493. https://doi.org/10.1002/wcms.1493

24. Spicher, S., & Grimme, S. (2020). Robust Atomistic Modeling of Materials, Organometallic, and Biochemical Systems. Angew. Chem. Int. Ed., *59*, 15665–15673. https://doi.org/10.1002/ANIE.202004239

Spectra

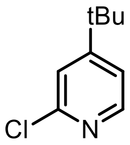

s14

s14 ^{1}H NMR (400 MHz, $CDCl_3$)

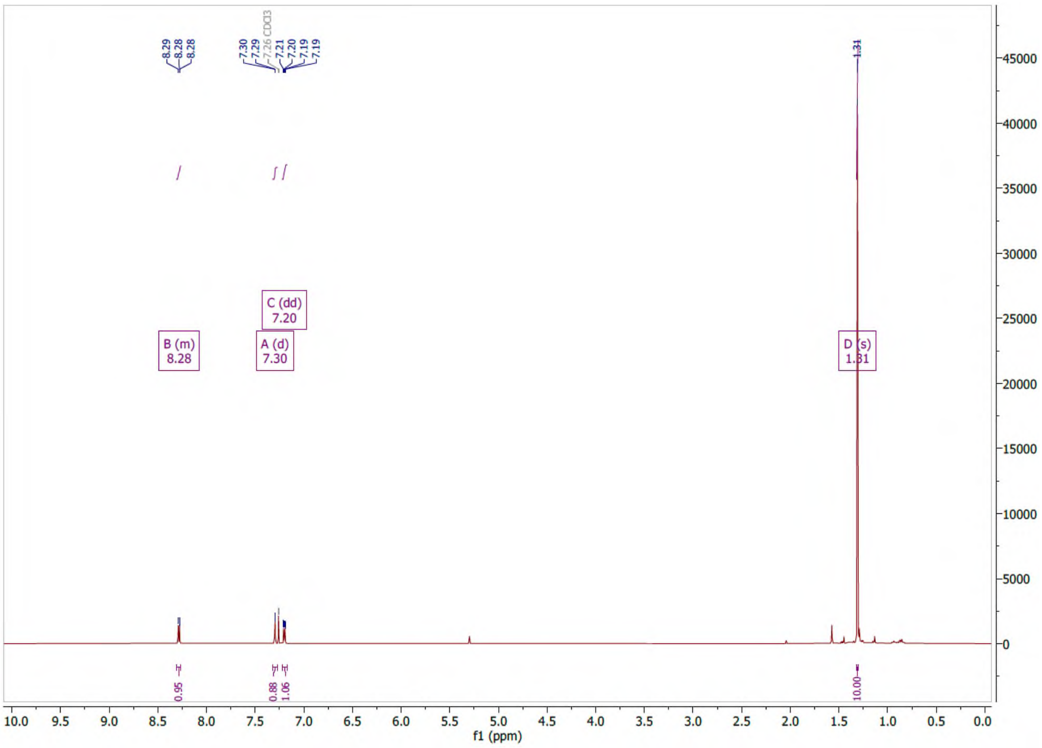

^{13}C NMR (101 MHz, $CDCl_3$)

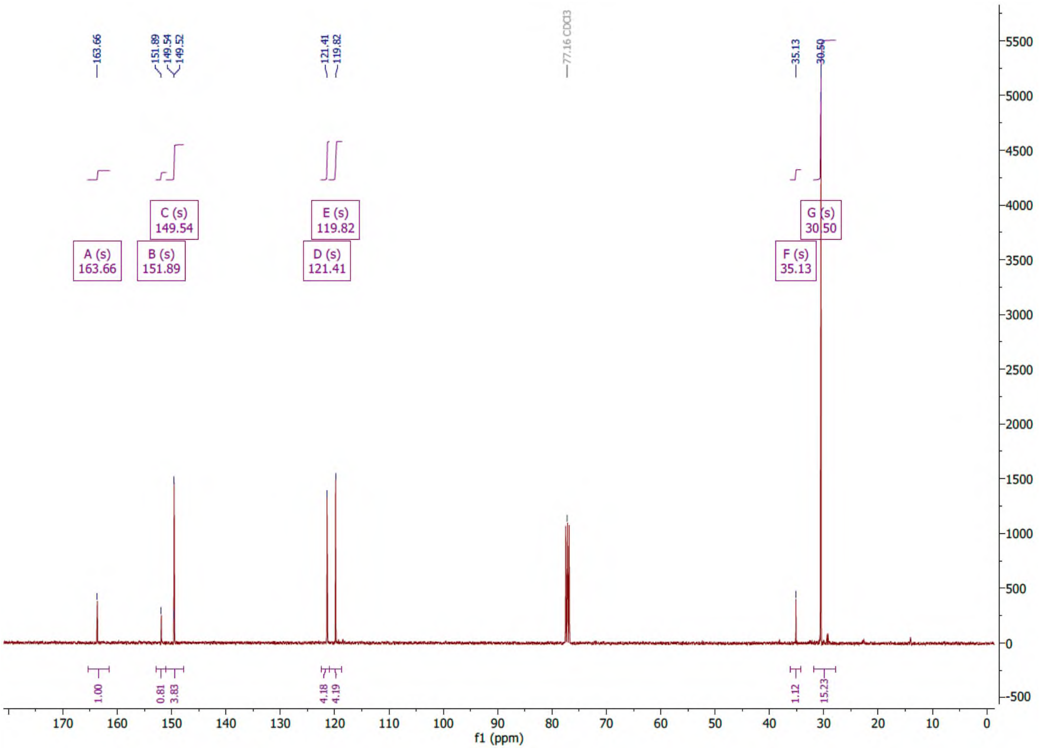

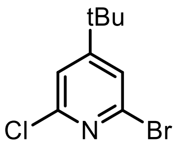

s15

s15 ^{1}H NMR (400 MHz, $CDCl_3$)

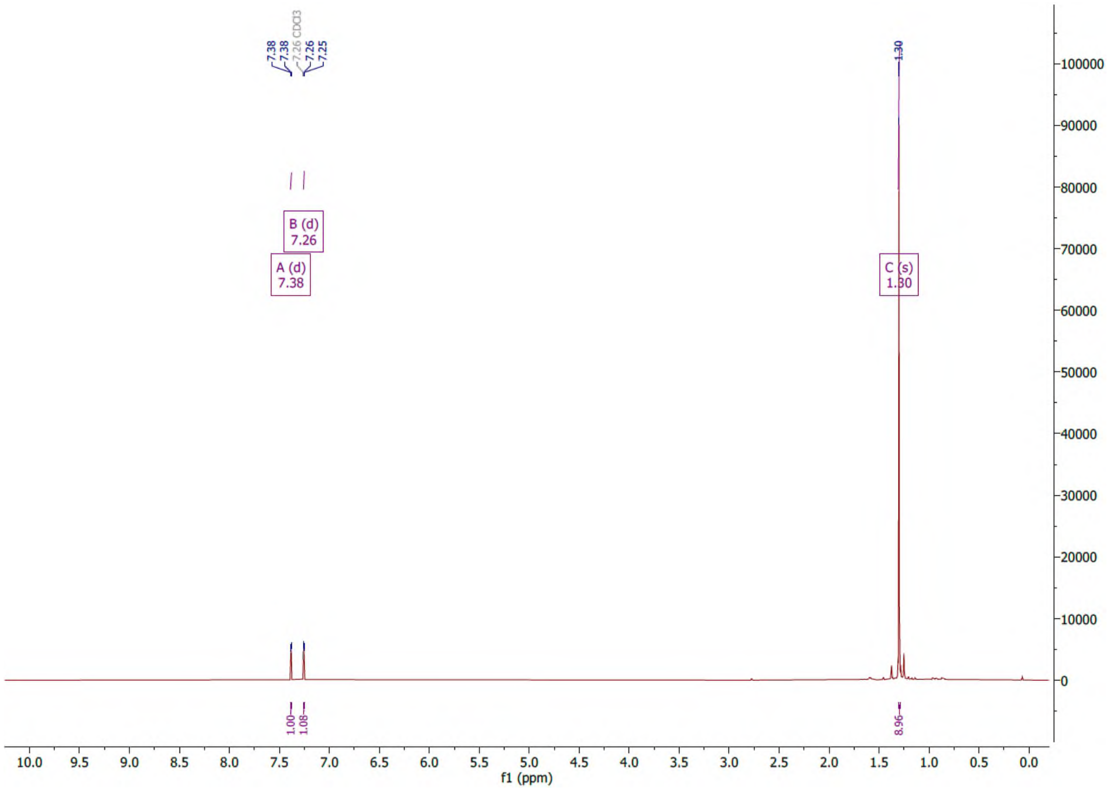

^{13}C NMR (101 MHz, $CDCl_3$)

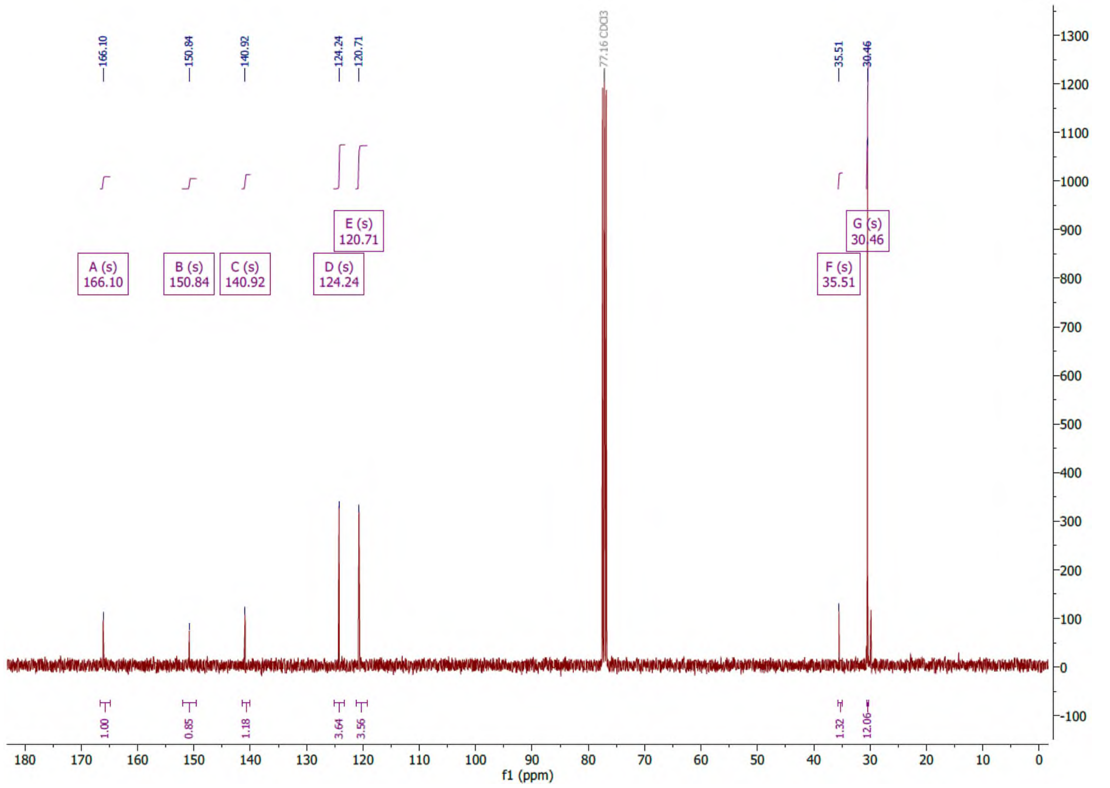

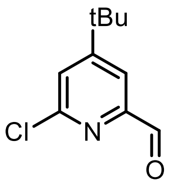

s16

s16 ^{1}H NMR (400 MHz, $CDCl_3$)

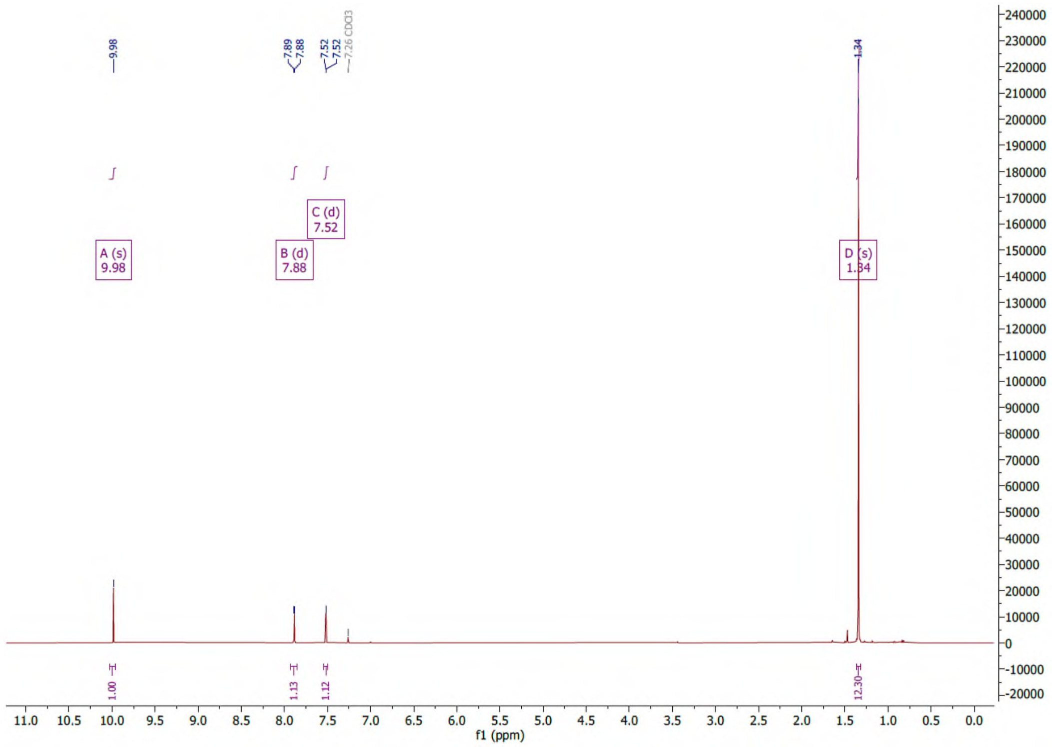

^{13}C NMR (101 MHz, $CDCl_3$)

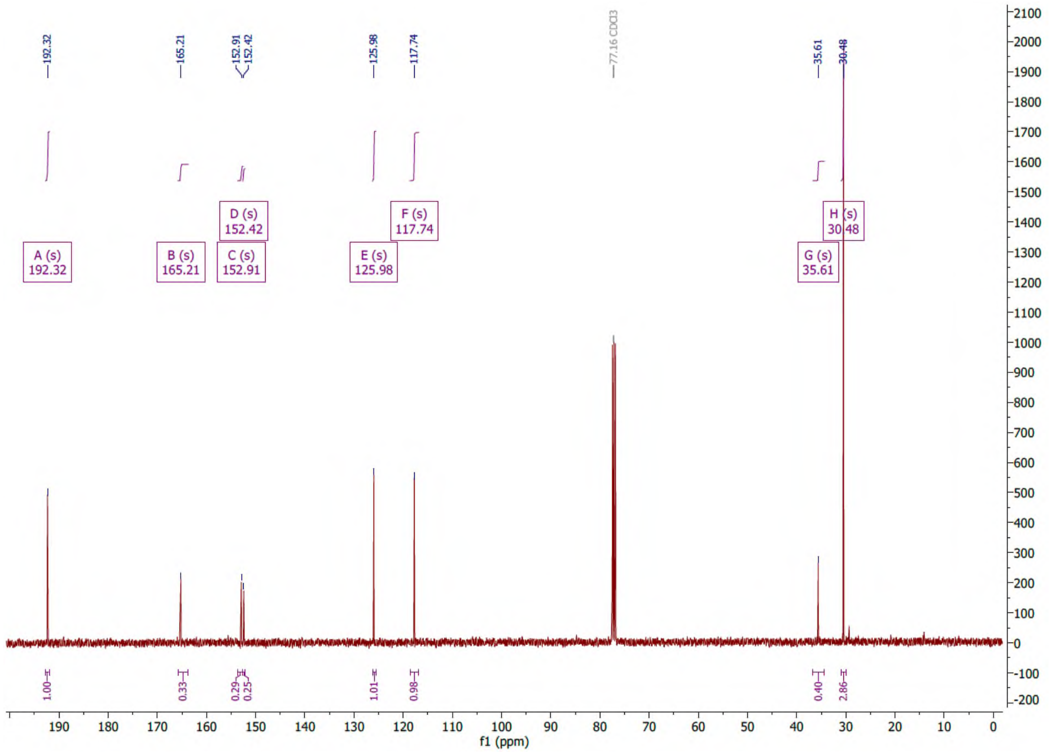

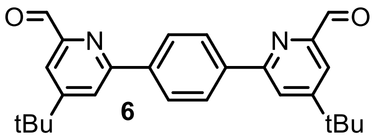

6 ¹H NMR (400 MHz, $CDCl_3$)

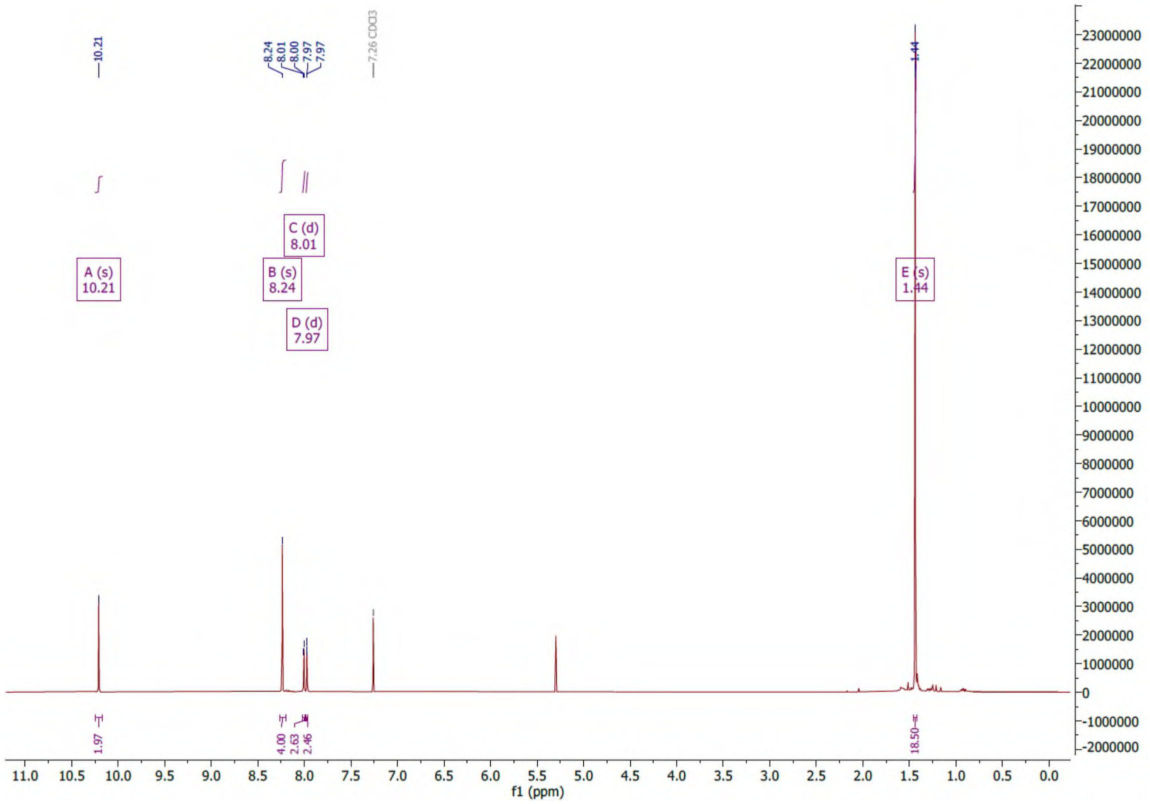

¹³C NMR (101 MHz, $CDCl_3$)

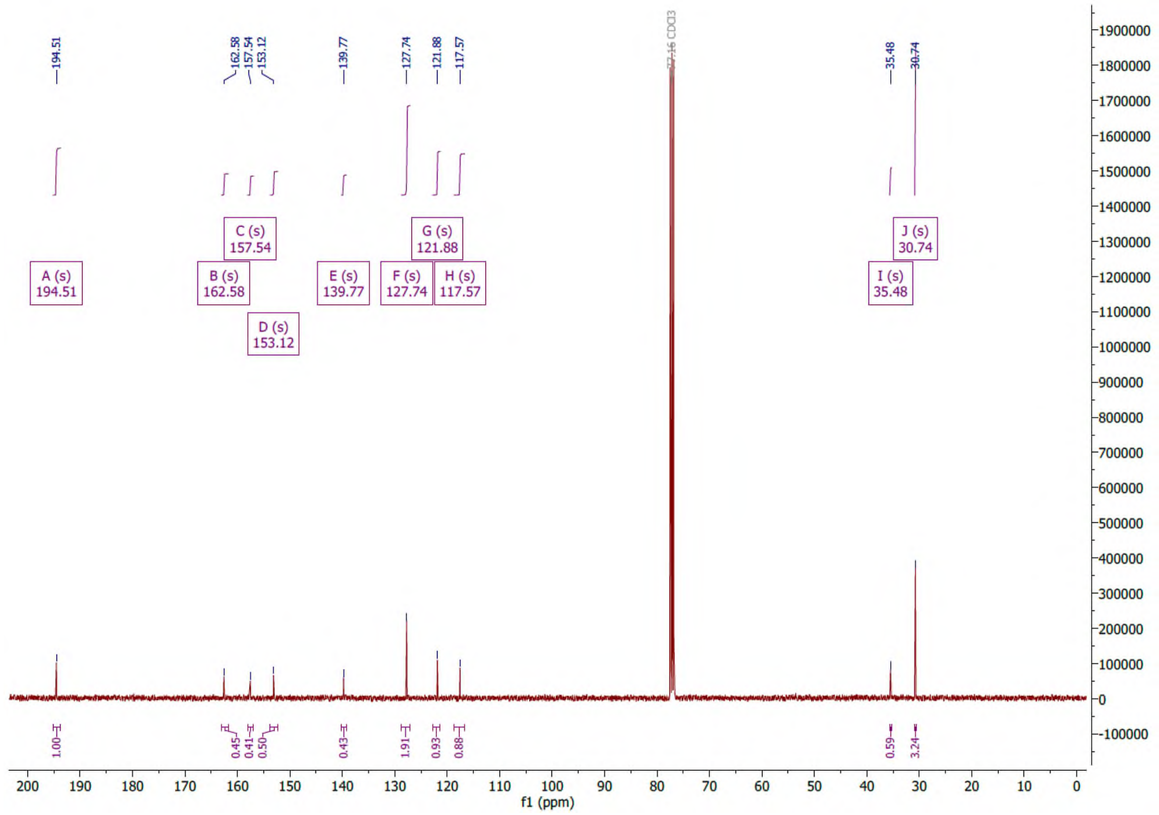

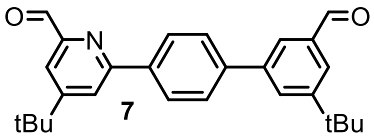

7 ^{1}H NMR (400 MHz, $CDCl_3$)

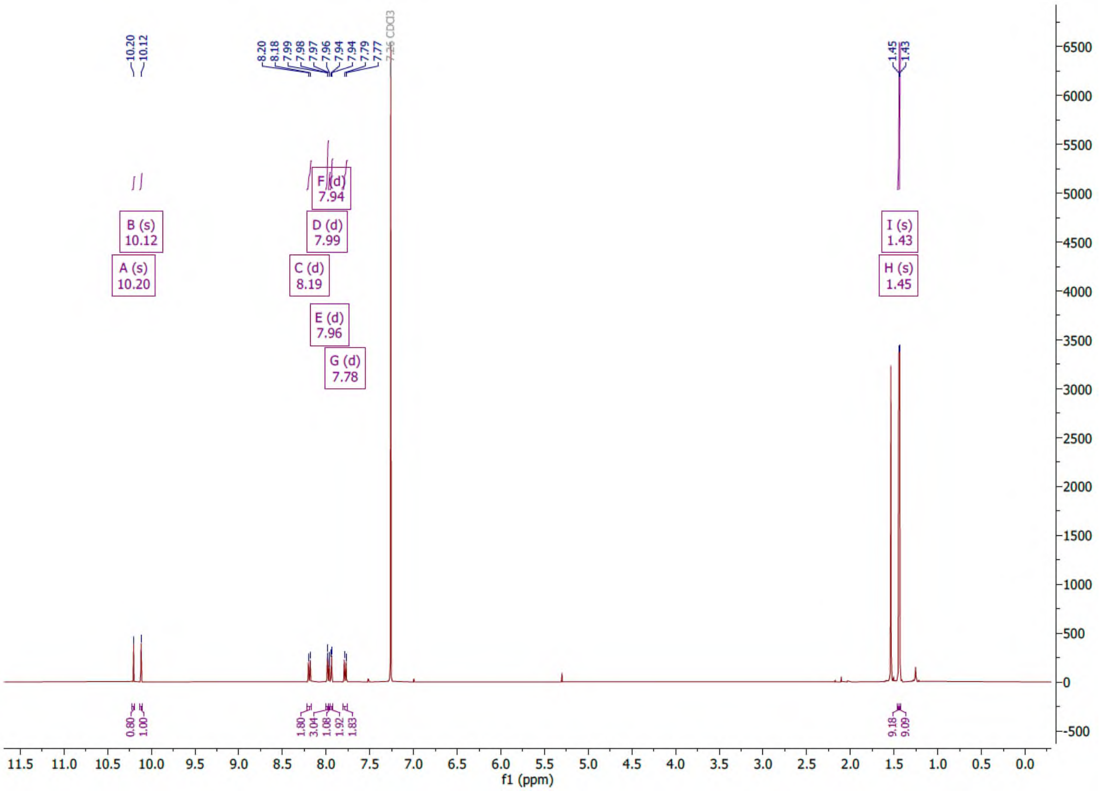

^{13}C NMR (101 MHz, $CDCl_3$)

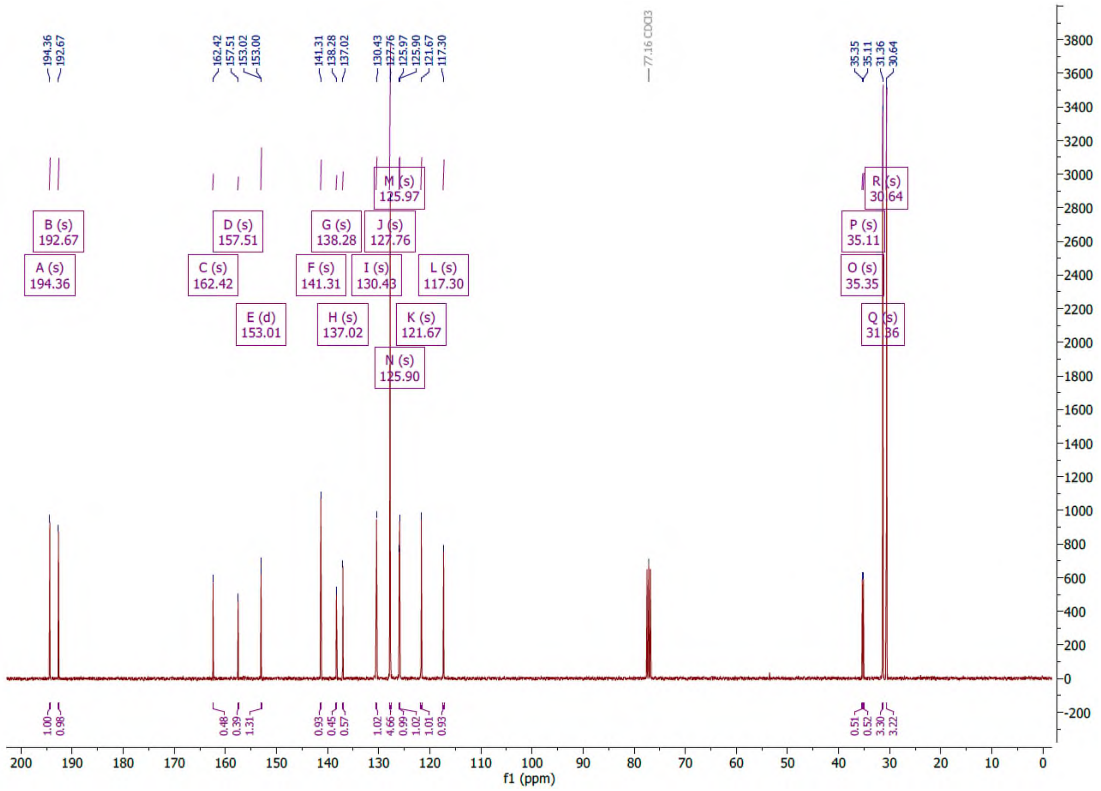

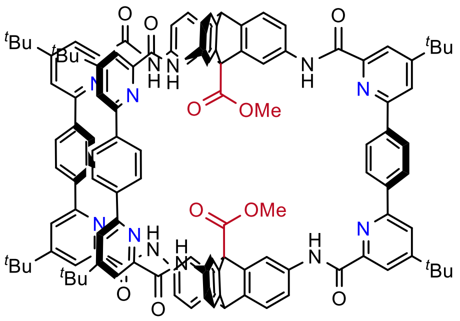

2e

2e ^{1}H NMR (400 MHz, $CDCl_3$)

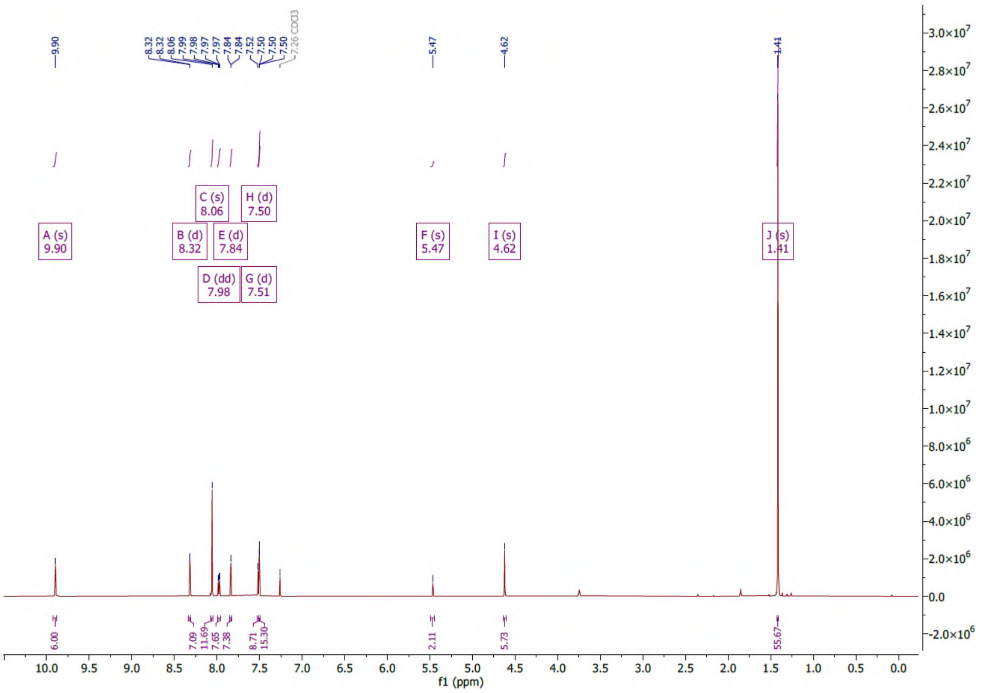

2e ^{13}C NMR (101 MHz, $CDCl_3$)

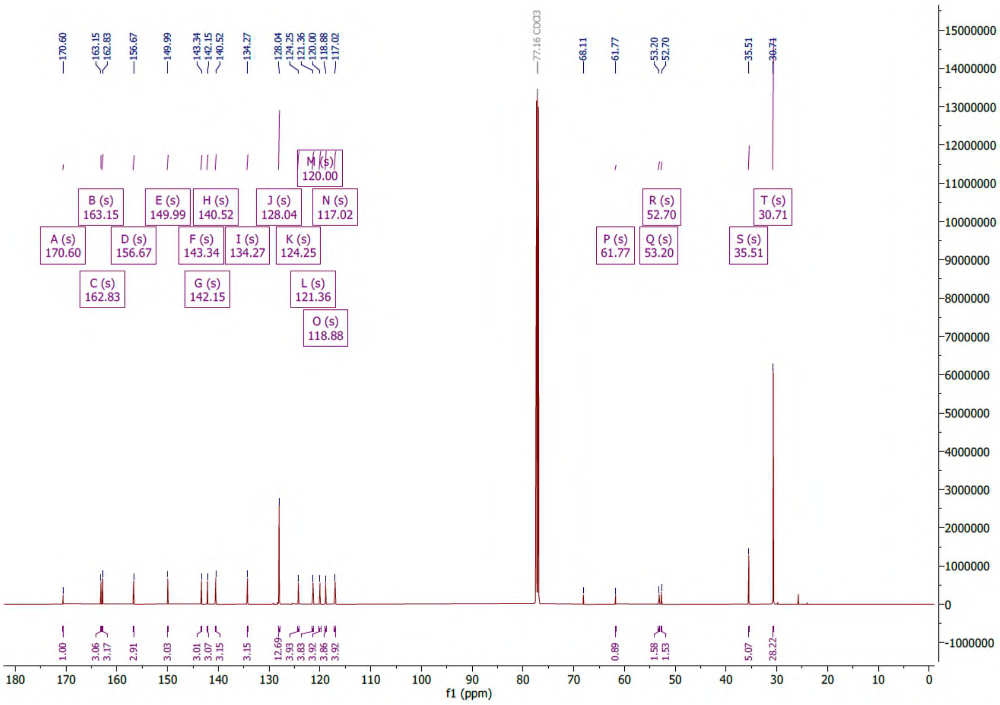

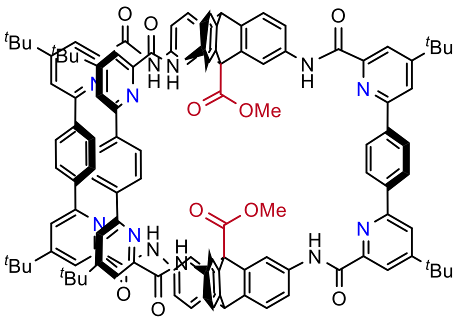

2

2e MS *m/z* (MALDI-ToF-RP) (DCTB matrix) M+H

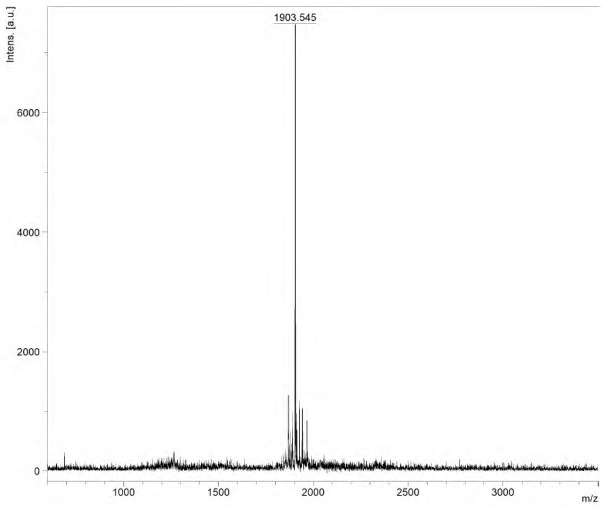

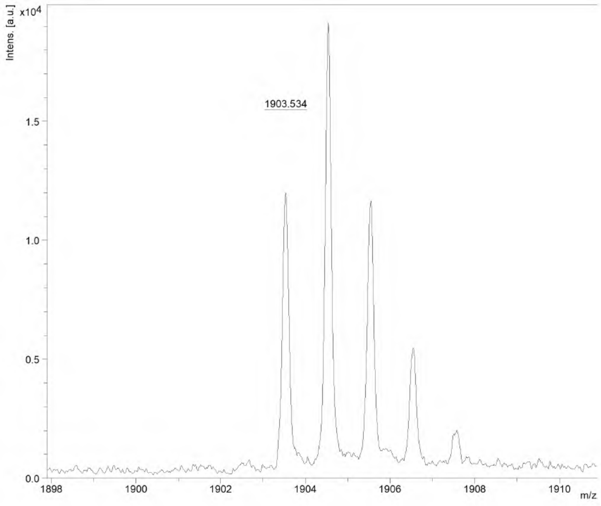

Calculated isotope pattern (top); measured (bottom)

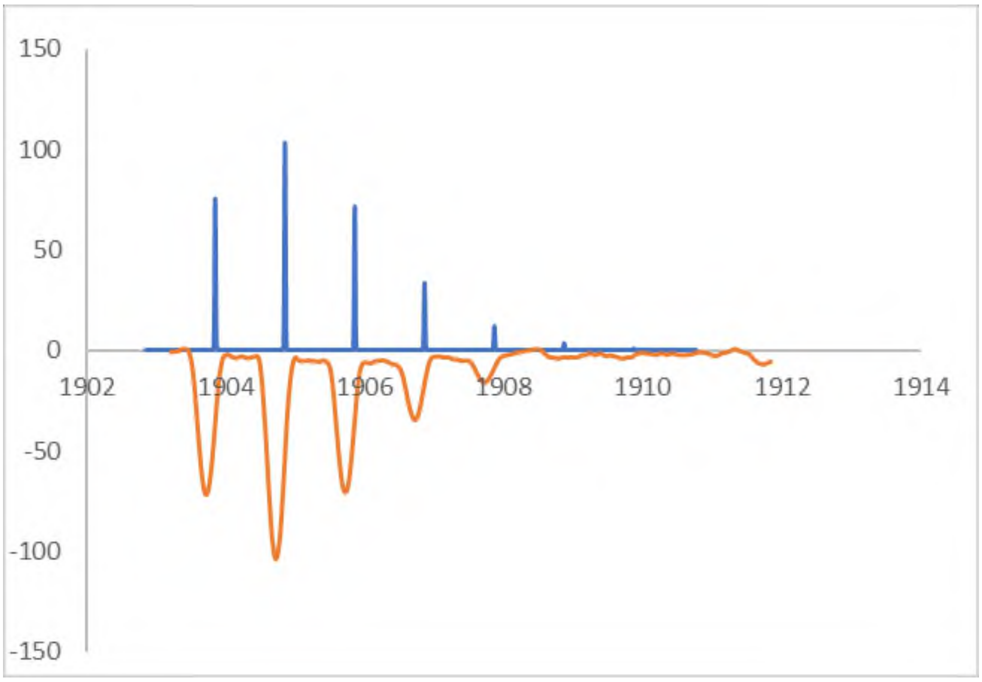

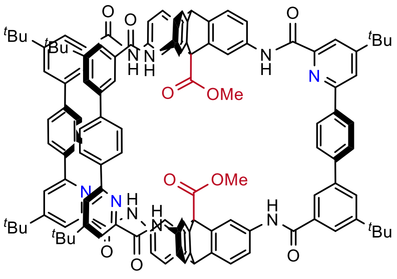

3e

3e **^{1}H NMR** (600 MHz, $CDCl_3$)

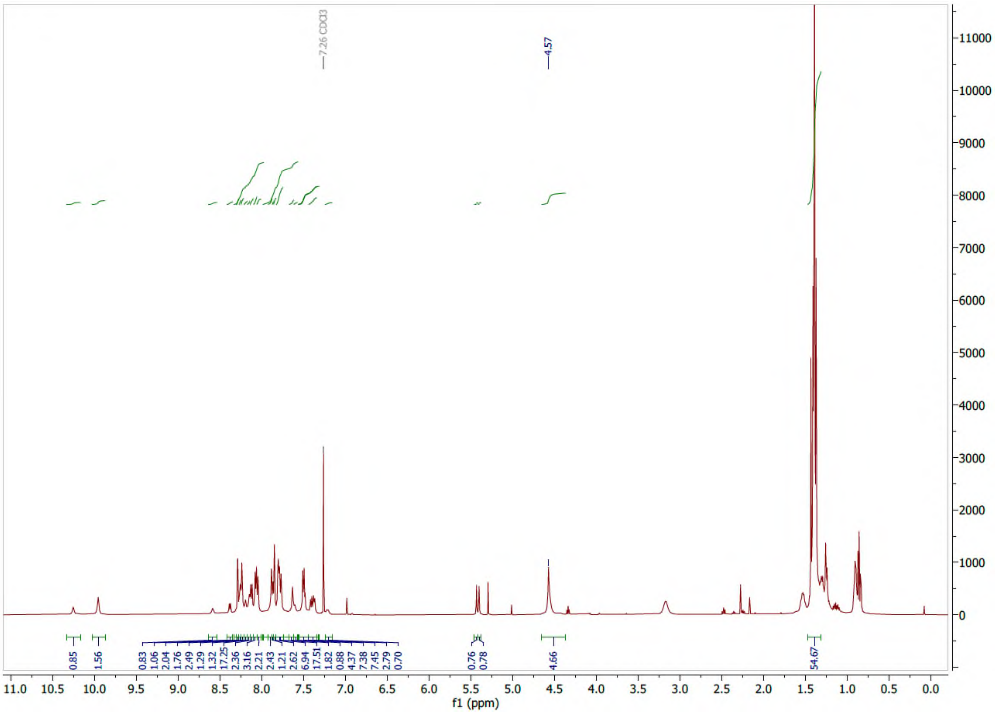

3e **^{13}C NMR** (126 MHz, $CDCl_3$)

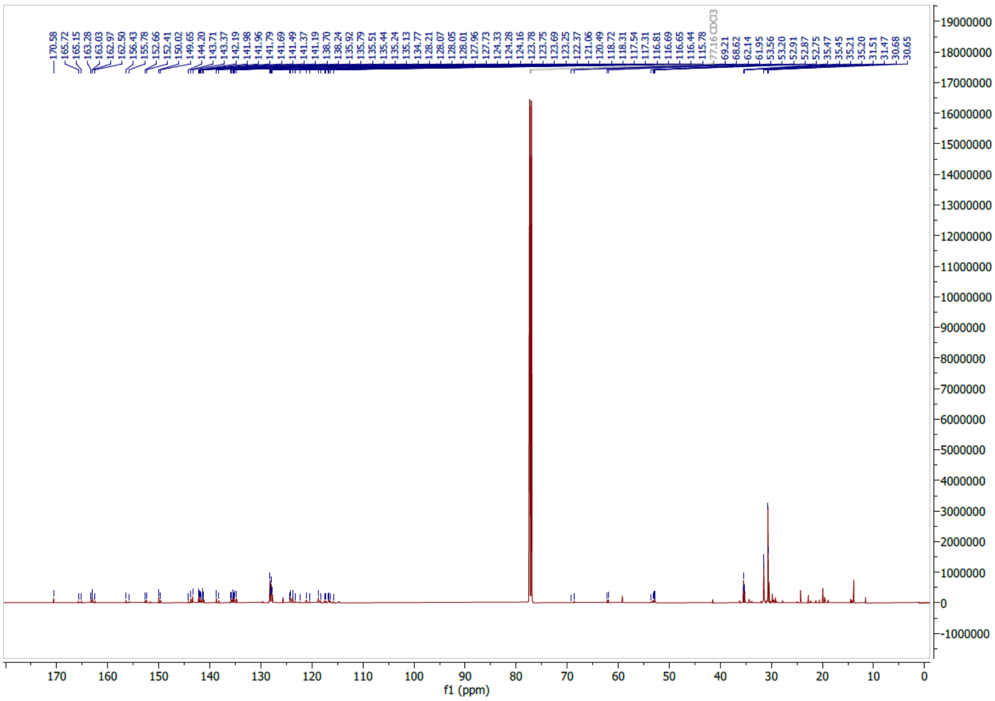

3e ^{1}H-^{1}H COSY NMR (600 MHz, $CDCl_3$)

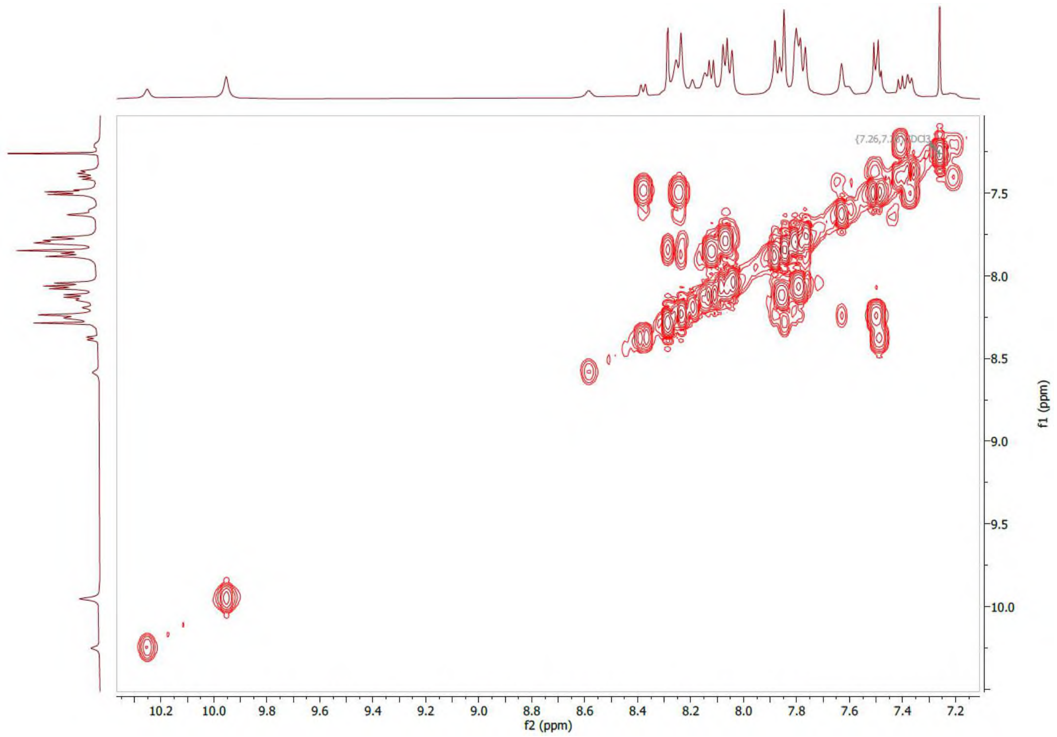

3e ^{1}H-^{13}C HSQC NMR ($CDCl_3$)

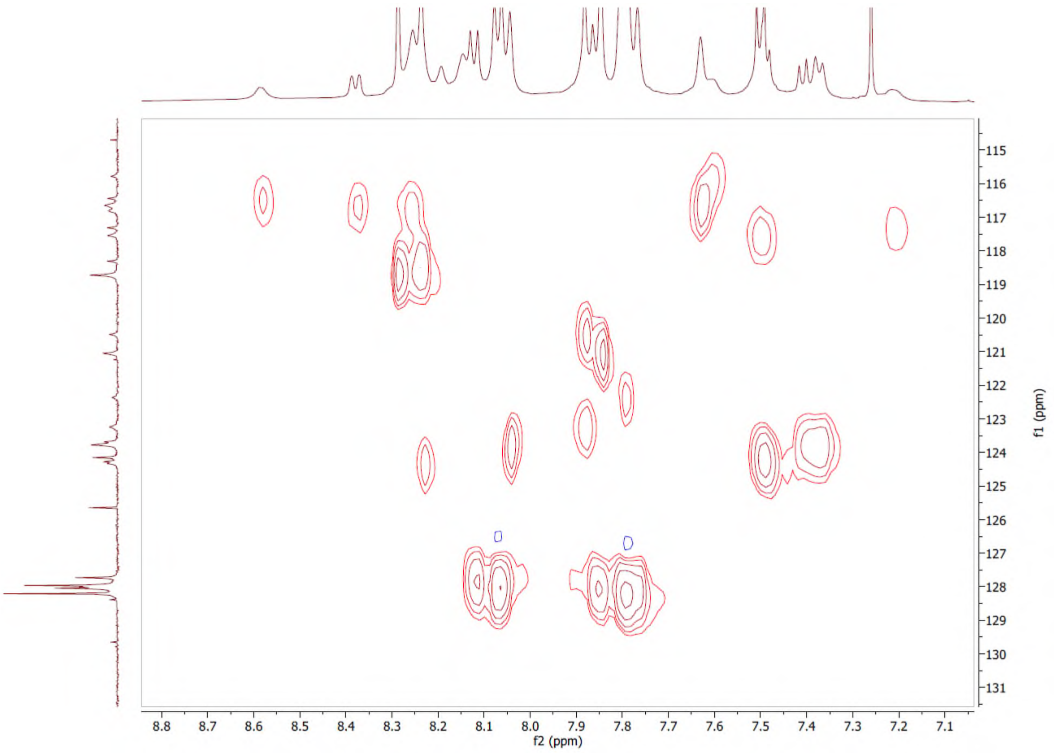

3e HMBC NMR (CDCl3)

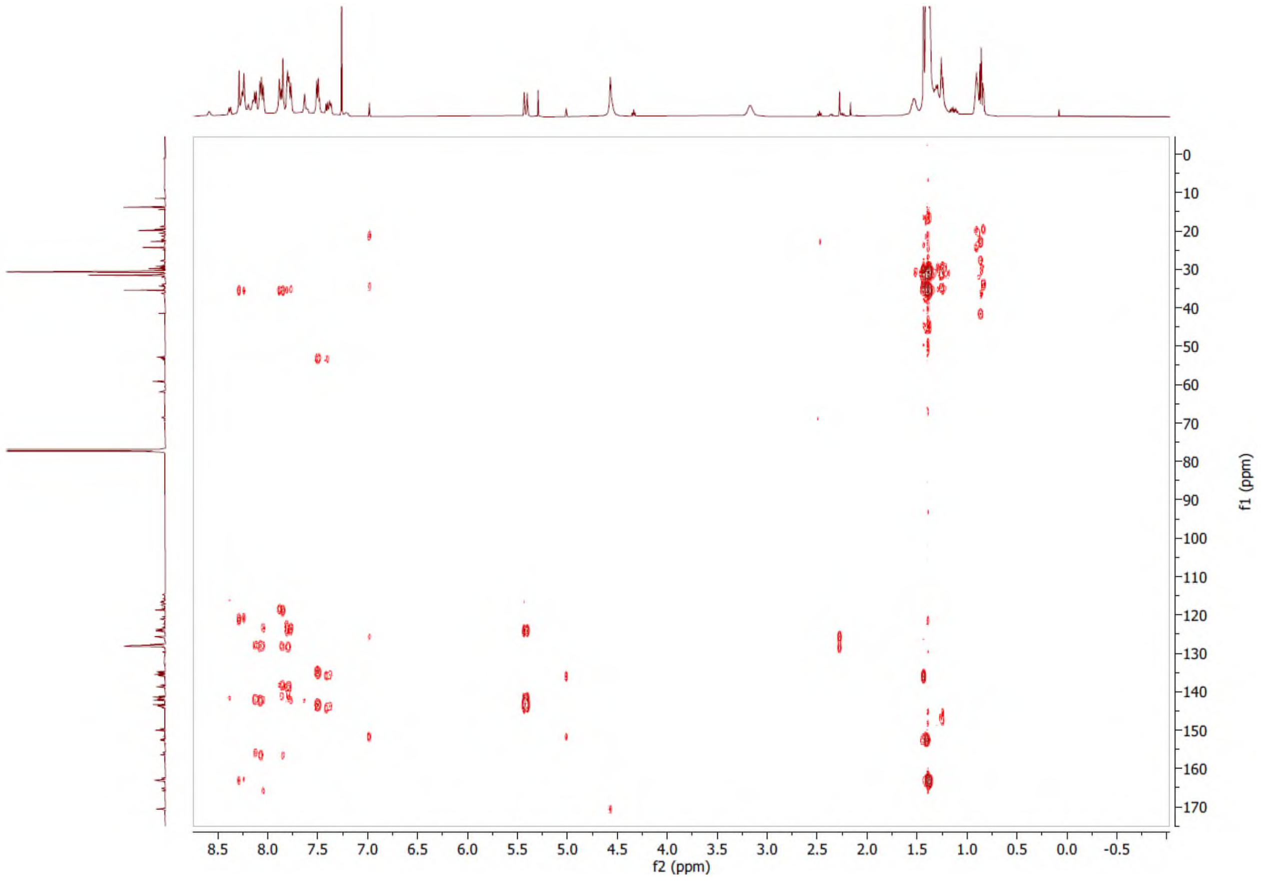

3e MS *m/z* (MALDI-ToF-RP) (DCTB matrix) (M+H, M+Na)

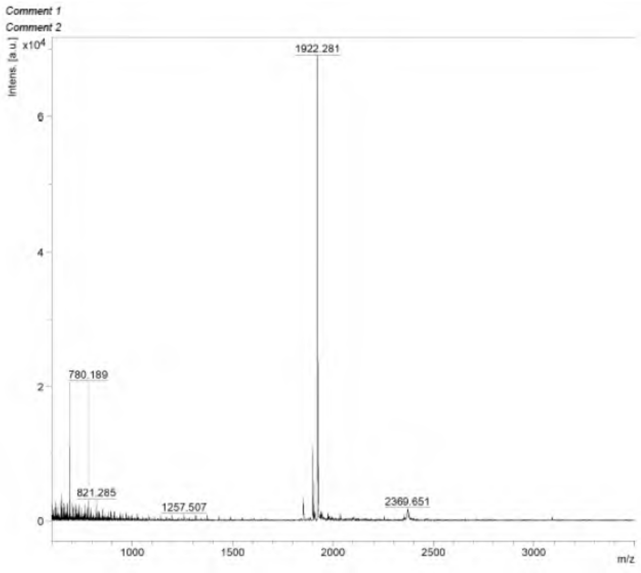

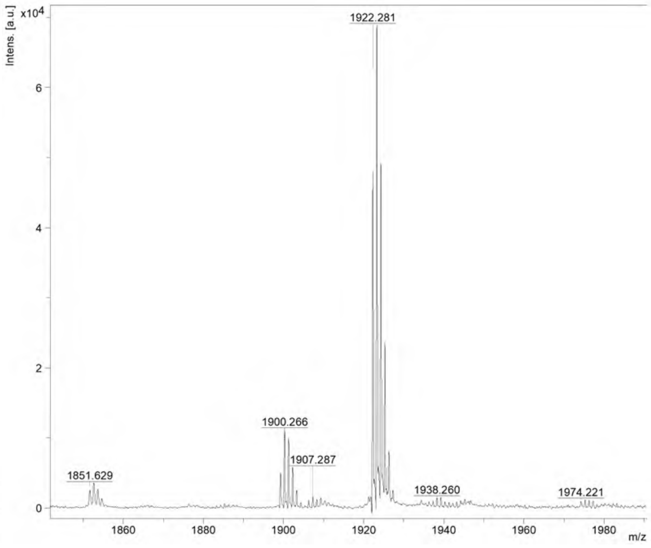

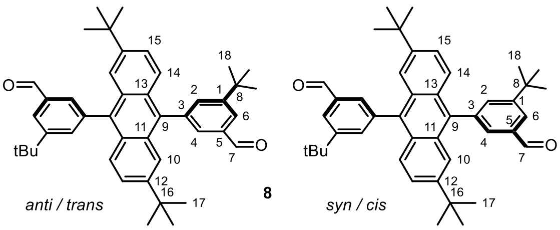

8 ^{1}H NMR (500 MHz, C_6D_6)

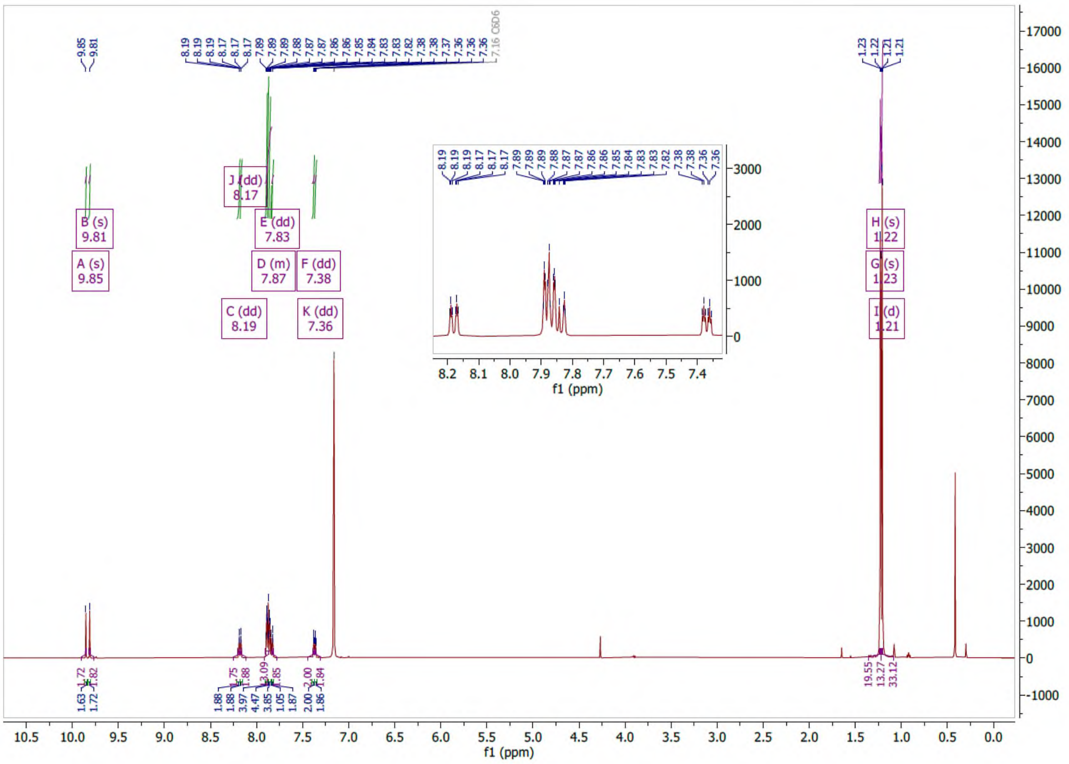

8 ^{13}C NMR (126 MHz, C_6D_6)

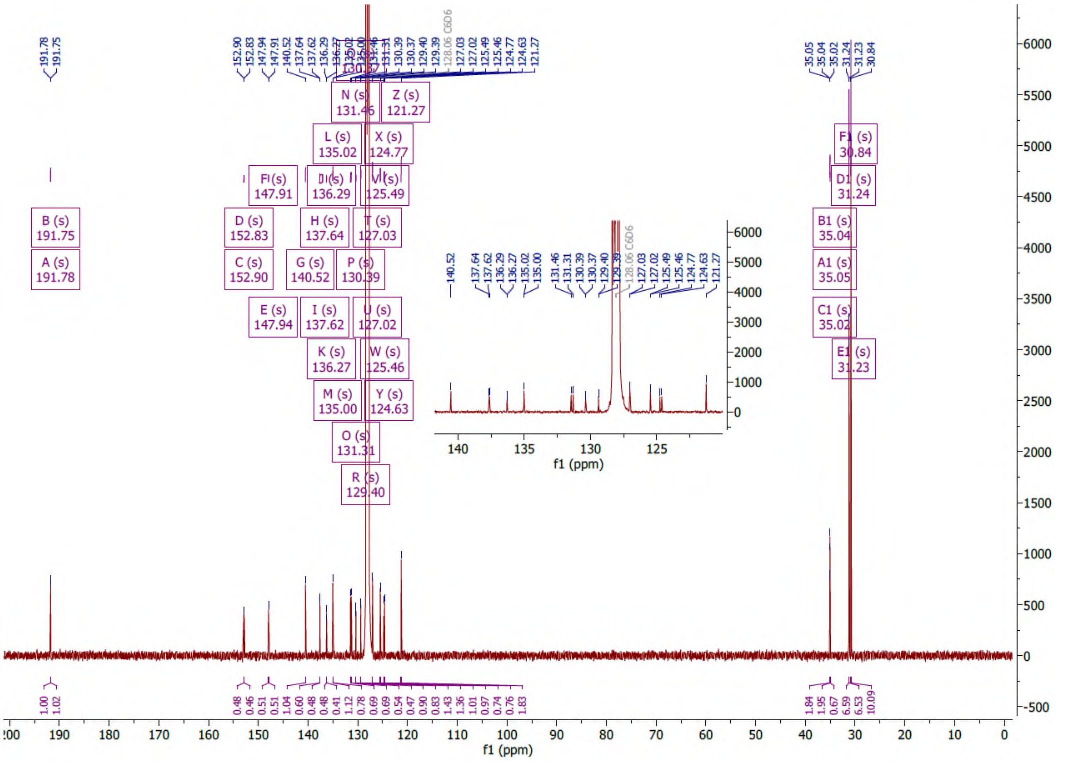

4e MALDI-TOF

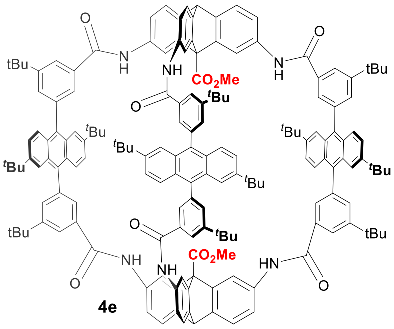
4e

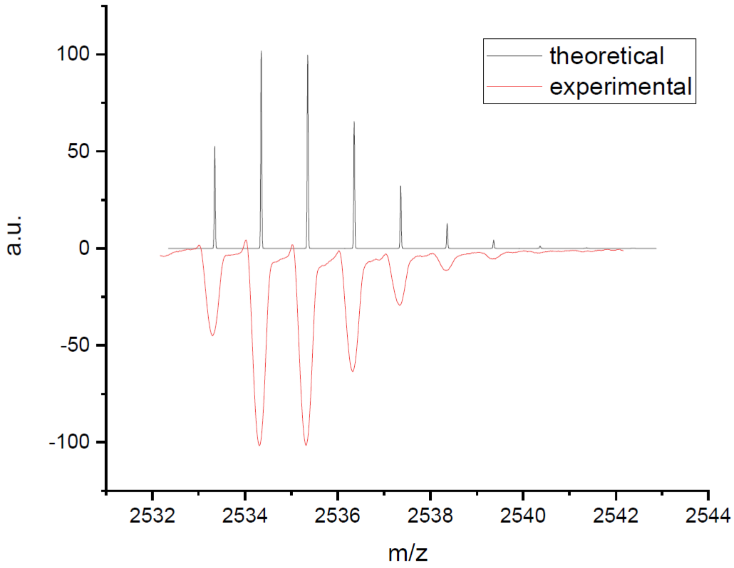

expansion, measured and recalibrated alongside cages 7a, 1e, 1, 2e and 2 for consistency

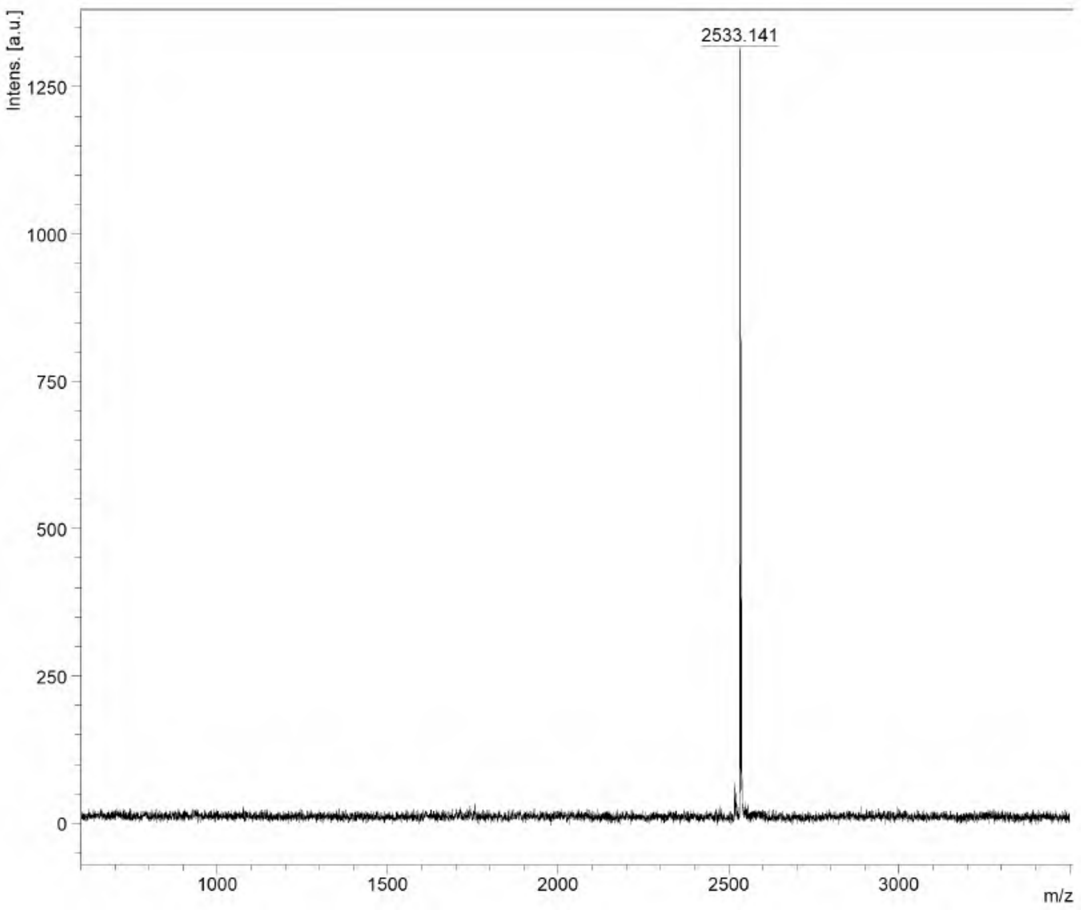

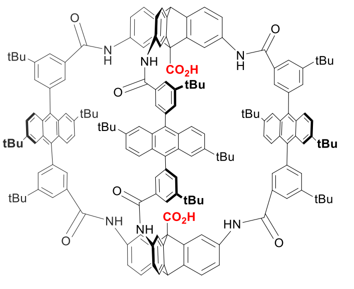

4

4 ^{1}H NMR (600 MHz, THF-d_8) (as a pair of atropisomers)

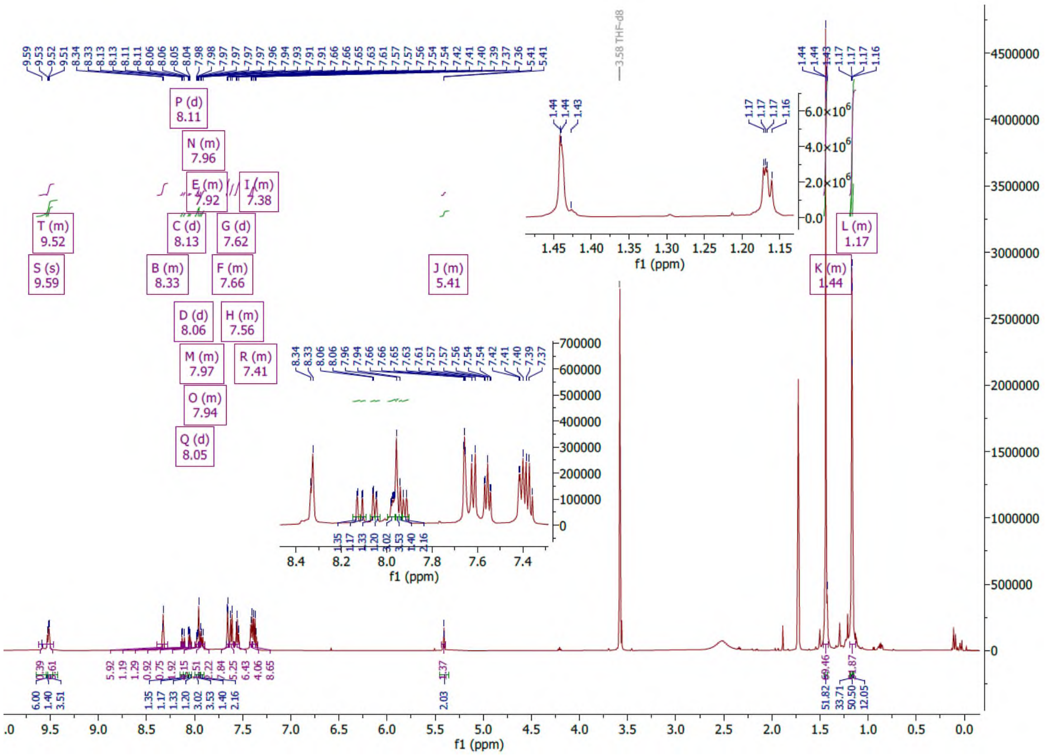

4 ^{13}C NMR (151 MHz, THF-d_8) (as a pair of atropisomers)

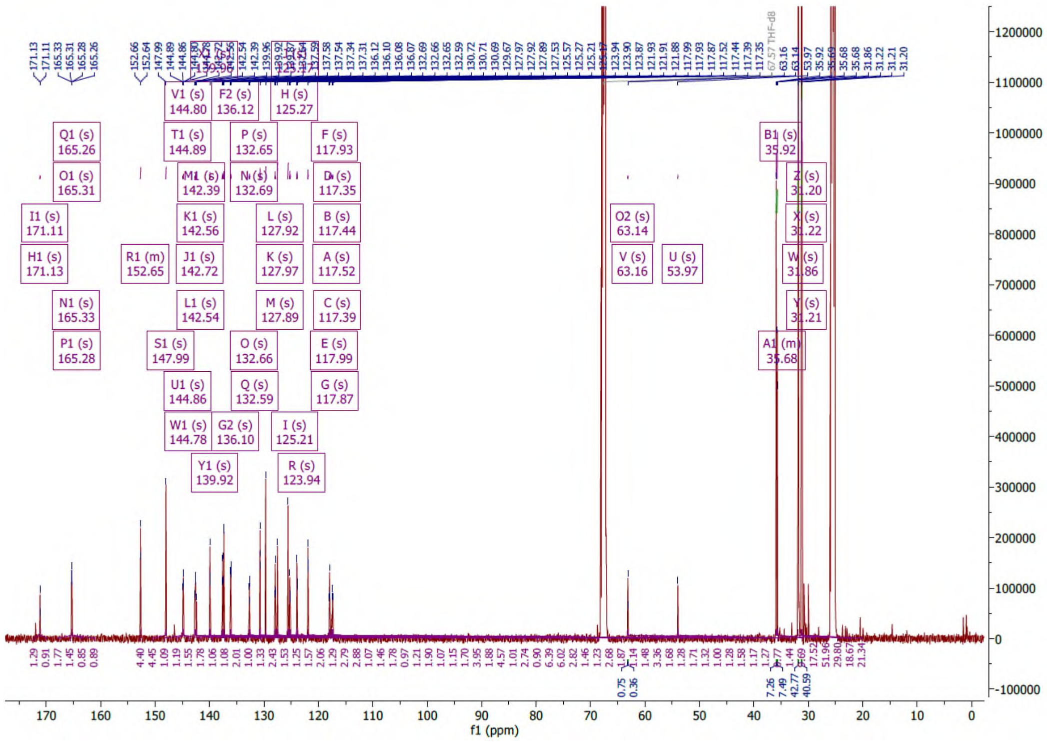

4 expansion of ^{13}C NMR (151 MHz, THF-d$_8$)
(showing the two atropisomers)

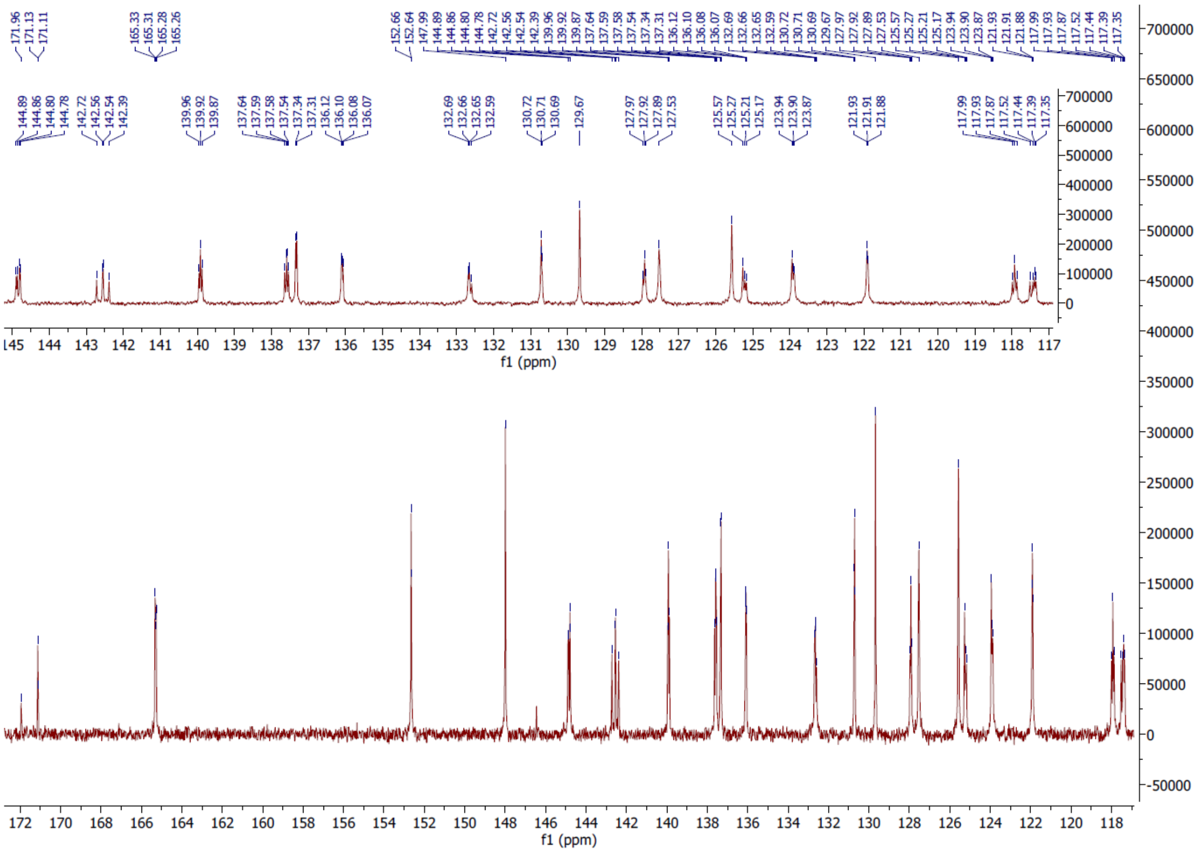

4 ^{1}H-COSY (THF-d$_8$)

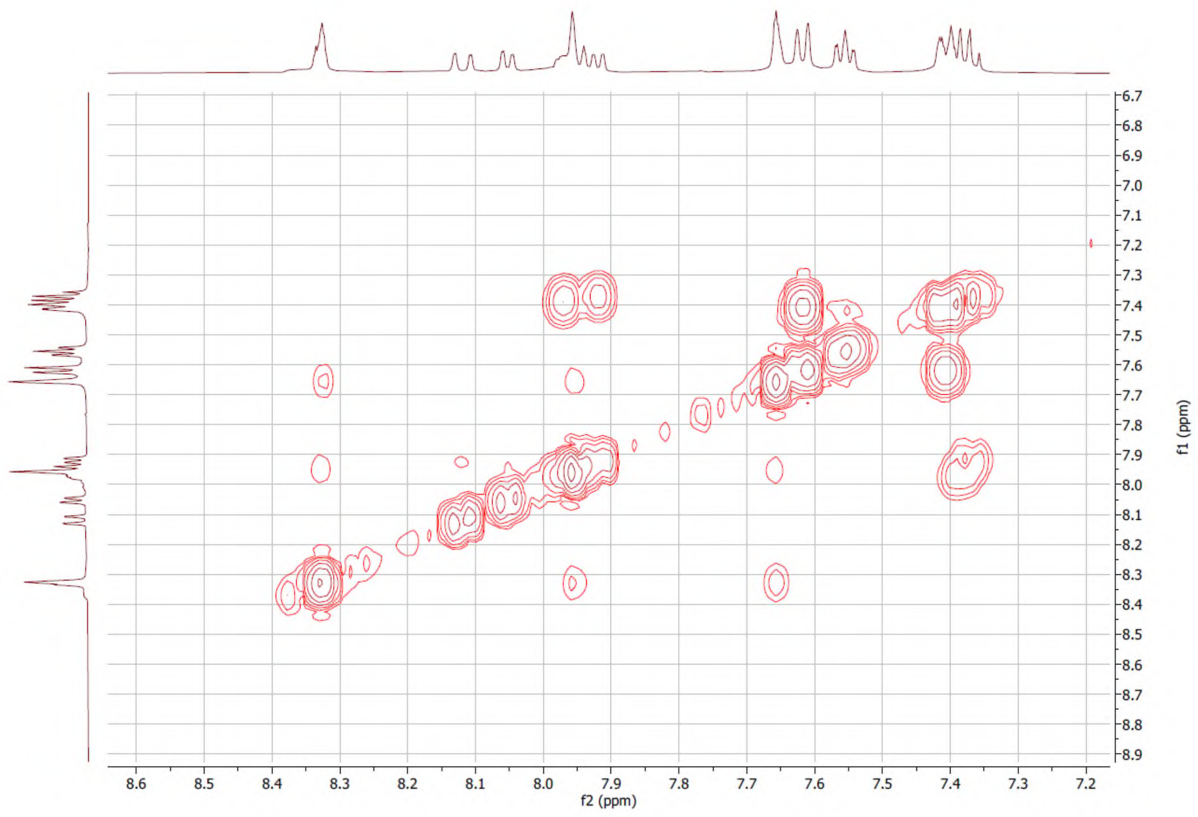

4 **HSQC (**THF-d_8**)**

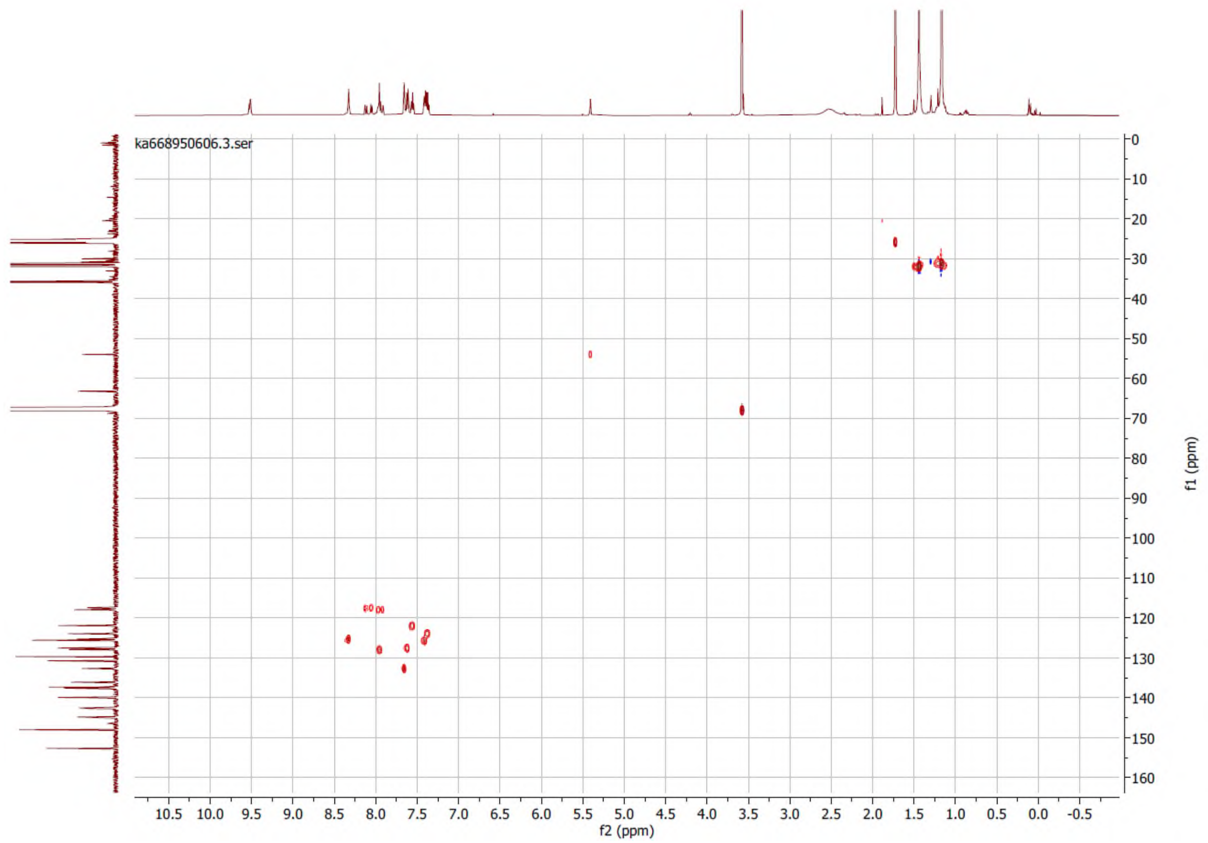

4 **HMBC (**THF-d_8**)**

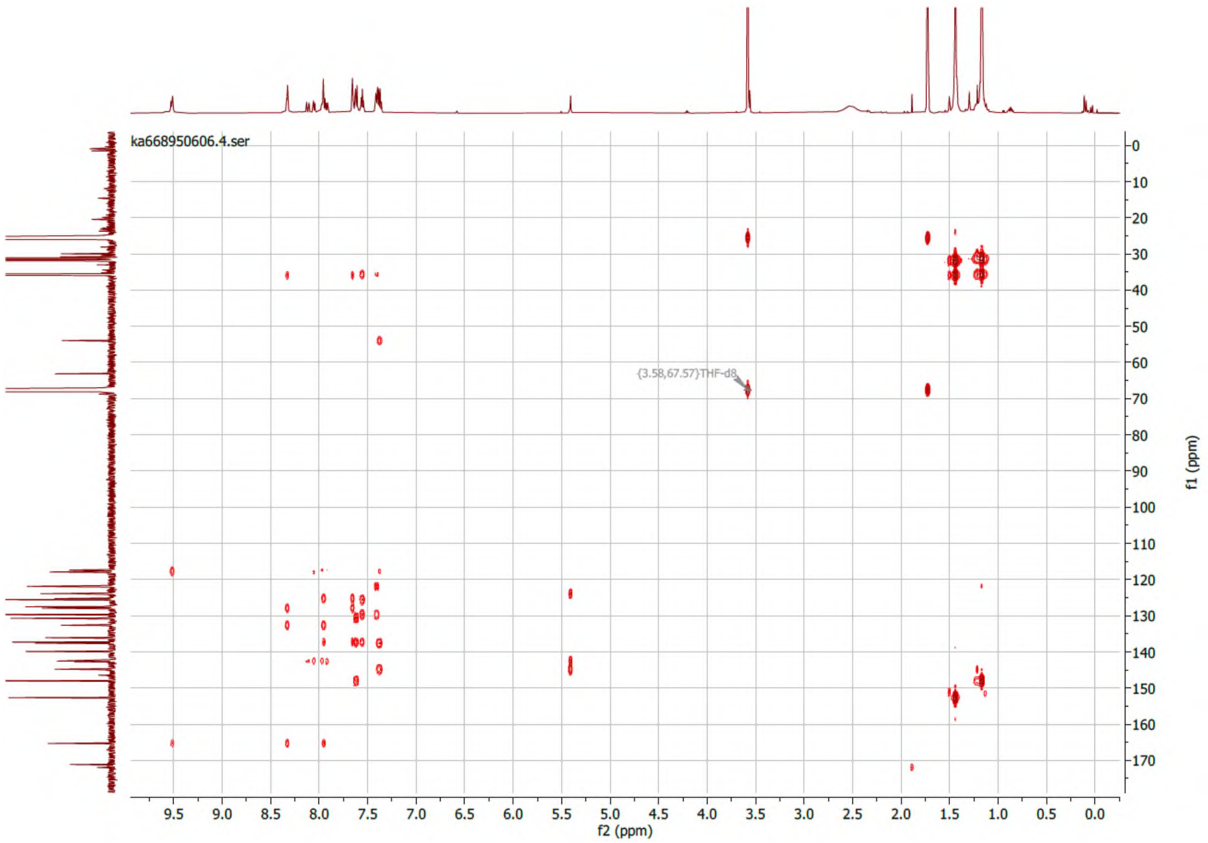

Reference annotations for:

Programmable Synthesis of Organic Cages with Reduced Symmetry

In order to facilitate the expeditious locating of supporting claims referenced in the main text, we include an annotated reference justification. The aim of the justification is aid transparency, and save time for readers. The trail includes:

- details of which tables etc quoted values are taken from;
- quotes from the original manuscripts to allow instant localisation of supporting claims using modern computer search functions;
- author comment on groups of references;
- longer footnotes;
- examples of references disputing a claim made in the manuscript, where appropriate.

**Introduction**	
It has long been known that supramolecular systems can host unique chemical environments not found in bulk solution or the gas phase.[1–7]	Reviews on reactivity and behaviour in confined spaces
These tailored environments are highly attractive for tasks such as sensing,[8] catalysis,[9,10] separation, [11–13] delivery,[14] and stabilisation,[15,16] to name a few.	Reviews of applications in organic cages, metal coordination cages, and capsules.
current self-assembly approaches due to the reliance on symmetric geometries to favour assembly by dynamic covalent chemistry.[17–19]	Reviews of dynamic covalent chemistry and reticular chemistry
Nonetheless, the successes of modern macromolecular cavity chemistry[8–11,14]	As summarised in relevant reviews
have inspired attempts to tune and reduce the symmetry elements of the cavities of self-assembled structures, to increase activity, selectivity and functionality.[20–25]	Cooper (organic cages review); Clever (review of increasing functionality in metal organic cages); Otte (decreased symmetry in cages); Lewis, Jelfs (desymmetrising coordination cages); Yaghi, Li (anisotropic reticular synthesis)
Promising cavity types include non-covalently assembled organic capsules,[26–28]	Catalysis in capsules
metal organic (coordination) cages[9,10,29–31]	Catalysis in coordination cages, and other applications,
and organic cages. [20,32–36]	Organic cage properties reviews; there are very few cage-catalysis examples for organic cages to date.
While rational methods to lower symmetry in coordination cages have	Including reviews and progress by Nitschke, Lewis, Jelfs and Clever.

gathered increasing momentum,[21,23,37–44]	
In addition to the semi-stepwise methodology of Otte,[22]	Otte uses an initial self-assembly of a macrocycle, and expands to a cage in a second self-assembling step.
one approach is to use computational screening combined with synthesis to assess viable formation of stable imine-linked cages when different types of multivalent aldehydes are mixed with multivalent amines.[45–48]	Cooper, Jelfs and Greenaway and coworkers have excelled in locating organic cages predicted to be stable computationally, and testing these experimentally.
Social self-sorting, narcissistic self-sorting[49,50] (including with chiral fragments)[47,51] and scrambling are possible outcomes,	Narcissistic self-sorting is common because it uses the natural symmetry inherent in shapes made with regular bonding vectors.
successful instances of reduced symmetry cages via self-assembly have been reported by He and Zhang,[52] Mukherjee,[50] and Cooper, Slater, Greenaway and Jelfs.[47,53,54]	successfully isolated desymmetrised cages are known for a **Tri^{2}Di^{3}** cage,[50] and a cage formed using a reduced-symmetry (C_{2v}) trisaldehyde,[53] **Tet^{3}Di^{6}** cages (see topology terms here)[48] with two different Tet groups,[54] as well as cages with chiral groups.[47] He and Zhang report an unusual [4+8] cage with $\boldsymbol{C_2}$ symmetry.[52]
outcomes are discovered[53,55,56] rather than designed,[57]	This is not to denigrate this remarkable approach; there is certainly an element of design in choosing the initial building blocks. The authors use the word discovery because, before the calculations are run, it remains hard to predict (rationally) which building blocks will form stable cages, let alone low symmetry cages. The discovery is whether the simulations were helpful or not, and whether a useful system results.<br><br>Cooper et all state:[57] "For the rational design of large organic cages via a bottom-up strategy, it is important to recognize that small changes in the bond angles between the reactive functionalities in the starting materials can have a profound effect on the outcome of the reaction. For example… …the addition of a single extra carbon atom into the vicinal diamine-functionalized ring resulted in a minor change to the bond angle between the diamine groups, which increased the size of the cage product from a 10-component [4 + 6] cage to a 20-component [8 + 12] cage. Likewise, Fujita demonstrated that slight changes to bond angles between pyridyl ligand donors significantly affect the structures of metal–organic polyhedra, which he referred to as "emergent behavior"."
For this reason, our approach has been to tune specific promising cage classes based on amide-linkages, which offer greater stability and post-functionalisation[58–62] options than the imine variants	Mastalerz has successfully employed phenol alkylation reactions on imine cages;[60] but more robust transformations are available for amide cages.[61]<br>Nitschke has discussed the many challenges associated with post-functionalisations of coordination cages.[58]<br>Otte has reviewed modification chemistry in organic cages, revealing that the field is in its infancy.[59]<br>Zhang and Jiang review recent post-synthetic modifications to porous organic cages.[62]
To this end, we recently reported methodology to access robust, soluble	We developed the in situ locking variant of the Pinnick oxidation[63] following work in which Mastalerz applied it to previously isolated

and functional organic amide-linked cages[63] using an *in situ* Pinnick oxidation locking approach,[64,65] which advanced important work by Mastalerz.[61,66]	imine cages;[61,66] Yaghi and Cui previously used it on isolated imine-linked COF systems.[64,65]<br>The value of the in situ approach is to access highly soluble or metastable cages which cannot be stably precipitated; we can therefore trap less symmetric species that do not crystallise/precipitate as readily.[63]
…that resemble the enzyme motif found in a broad family of aspartyl proteases and glycoside hydrolases.[67,68]	For instance, Bugg discusses the HIV aspartyl protease in figure 5.19 of the third edition. Lysozyme is discussed in figure 5.38 of the same edition.
**Results and Discussions**	
**(A)**	
did not crystallise in the naively expected symmetric D_{3h} geometry that defines the trigonal prism cage "topology"[48] (often termed [2+3] or **Tri^{2}Di^{3}** cages[48]).	Jelfs *et al* set out the common topologies of organic cages, and explain the notation system, in which Tri=tritopic=three bonding vectors; Di=ditopic=2 bonding vectors; and the superscripts refer to the ratio of instances of each building block in the cage.
– there are 13 unique permutations of six carbonyl orientations for planar[69] *trans*-amides,	Pros and Bloomfield discuss the conformational preferences of phenylbenzamides; the *trans* planar amide is preferred.<br><br>In our hands, c*is* amides of cage **1** were infrequently observed during conformational sampling, and the few that were observed were always too high in energy to be present in significant quantity e.g. <0.001% population. In practice, the amides are only approximately planar due to macrocyclic strain
This is readily understood by noting that the amide bond linkages deviate from linearity: the C$\widehat{N}$C angle opens to 129.5°, whilst the N$\hat{C}$(=O)C angle narrows to 114.8° (**Figure 2a**)[69,70] and so each terphenyl edge piece can project different bonding vectors…	Figure 2 in Bloomfield shows statistics of amide bond rotations.[69] Hamilton in Figure 2 depicts angle deviations from linearity for amides.[70]
is less costly than permitting bond angle strain from the angle deficit.[71,72]	Conjugated systems such as biaryls are stabilised by orbital overlap and the possibility of resonance. Initial rotation of adjacent biaryls causes only minor loss of this overlap energy. In contrast, bending bonds induces higher strain at lower angle gain.<br><br>We cite here two examples in hydrocarbon systems where the small cost of minor twisting of C-C pi systems (Fig 2,3[72], Fig4[71]) is contrasted to the increased cost of bending.
**(B)**	
Use of hydrogen bonding to override geometric preferences has been applied widely, from helical peptides[73] and macromolecules[70,74] to organic cages.[75]	We highlight Gellman's foldamers,[73] amide bond deviations and control employed by Gong[74] and Hamilton,[70] and Mastalerz's use of phenols for constructing an organic cage.[75]

due to hydrogen bonding (or by reducing N/C=O dipole clashes).[70]	Hamilton discusses the relevant conformational preferences observed in the Cambridge structural database in figure 2.
**(C)**	
Recent work from Cooper, Jelfs, and Greenaway has focused on using computational screening to predict imine cage assembly.[53,55,76,77]	Approaches typically involve calculating the expected populations of all possible cages that might form from some collection of building blocks and focussing attention on combinations that are most likely to give isolable material. This is a requirement because mixtures of imine cages are typically difficult to purify due to their instability to hydrolysis or scrambling, which is not necessarily true for amide cages.<br>A second stage is to calculate what physical state hypothetical solids might exist in, and choose possible cages according to their predicted properties. This is a broad screening approach, and differs from the cavity tuning approach which aims to develop and improve specific architectures.
or one can be distal ("up-up-down" = **UUD**: 3/4 chance) (**Figure 4a**).[50]	Mukherjee observes these statistics in a different system.
as often observed for lower symmetry cages.[53]	Jelfs and coworkers were forced to use predictive methods to assign a tentative structure in the case of a low symmetry cage which did not crystallise.
Tuning of cage windows[78] or cavity size is a key technique for tuning cage properties.[77]	The PyWindow software sometimes provides useful pore window sizes, though the method does not work well for the very open cages used here.<br>Slater et al have used pore tuning in materials design.
Modifications at the periphery typically alter the window size, although they can also influence cage topology.[79,80]	Cooper et all discuss how external cyclohexane substitution of diamines affects cage topology.<br>Clever discusses some "steric" engineering to the same effect.
are predicted to translate their axial configurational chirality to a conformational helical chirality.[42,47]	Raymond discusses translation of chirality into structure.<br>Slater et al show how chiral pieces can result in contrasting cavities.
**Discussion**	
Many current approaches to access low-symmetry cages use geometrically unsymmetric edge pieces.[53,80]	Jelfs and coworkers use an edge piece with different numbers of aldehyde at the top/bottom.<br>Likewise, Clever reviews recent low symmetry cages, many of which use unsymmetric edge pieces.
The observation that a symmetric assembly can relax into a reduced symmetry conformation is not new, but instances are usually "noted" rather than exploited. [50,53,81–84]	In all these references, the reduced symmetry is observed, but not predicted; noted, but not explained. We take this as an immense marker of the importance of codifying this topic to aid design strategies.<br>The most relevant discussion comes from Chand: Chand has reported M_2L_4 cages demonstrating mixed conformation pairs in diurea cages[81,82] and self-sorting in unsymmetrical amido-pyridyl ligands.[83]<br><br>In the case of a urea cage showing conformational bias (cage 2),[81] the rationale seems to be in the ligand preference: "The strong preference of trans/trans conformations around (C)urea−(N)urea bonds in acyclic 1,3- disubstituted urea moieties is well-known, wherein the carbonyl bond and N−H bonds are oriented in opposite directions." The SI (Figure S2) shows which ligands are plausibly geometrically suited for metal coordination in isolation. Chand states of cage 2: "It can be seen that NH protons of the diagonally located urea moieties are either all endohedral or all exohedral." But this observation is not discussed further. The ureas are likely rotating: "Slow conformational

	changes at the ligand backbone could be the reason behind the signal broadening”. This appears to be a plausible example of *conformational autodesymemtrisation*.<br><br>In one clear case of conformational bias, counter anions are discussed as a rationale:[82] “The cage configurations can be manipulated by anions having different size and shape.”<br><br>Chand’s discussion of the origin of the preference of one self-sorted cage reads:[83] “The higher degree of diastereoselectivity during the self sorting can be probably attributed to the geometric complementarity/constraint provided by the ligand design.” Although the effect is similar to the results we describe, this system is not a conformational preference effect.<br><br>Separately, Jelfs et al recently state: “Mukherjee et al. have recently observed a self-selection process between multiple structural isomers when an unsymmetrical ditopic building block was employed for the synthesis of imine cages, but such processes are very hard to anticipate[53] “<br><br>Separately, Mukherjee and co-workers reported the following:<br>“…which established the unequivocal formation of the single isomer **II**, rather than a mixture of isomeric cages (**I** and **II**)…”<br>“To our surprise, when aldehyde **B** was subjected to the reaction with amine **X** under the same reaction condition, a mixture of products was found to form…”<br>“Interestingly, X-ray crystallographic analysis revealed the selective crystallization of isomer **II**. Such a phenomenon could be related to the self-selection process during crystallization…”
to equally distribute strain.[85,86]	Ring strain is largely the sum of bond angle strain, caused due to individual bond angles deviating from the ideal (e.g. relative to the isolated amide fragment). *Polymacrocyclic* strain arises across the entire structure. Necessarily, the lowest energy species will minimise strain, and each e.g. bond will not take on more “strain” than any other (which tends to favour symmetric conformations).<br>Shiotari discusses types of strain,[86] including “Baeyer” strain, which is discussed further by Wiberg.[85]
“Self-sorting” describes the *configurational* assembly preference of components in a self-assembling mixture.[87,88]	Wurthner’s review describes self-sorting.
We thank the EPSRC UK National Crystallography Service at the University of Southampton for the collection of some crystallographic data.[89] We acknowledge the use of the University of Oxford Advanced Research Computing (ARC) facility in carrying out computations.[90]	

REFERENCES FOR ANNOTATIONS FOR MAIN TEXT (for ESI references, see page S59)

1. Raynal, M., Ballester, P., Vidal-Ferran, A., & Van Leeuwen, P. W. N. M. (2014). Supramolecular catalysis. Part 2: artificial enzyme mimics. Chem. Soc. Rev., *43*, 1734–1787. https://doi.org/10.1039/C3CS60037H

2. Terra, J. C. S., Martins, A. R., Moura, F. C. C., Weber, C. C., & Moores, A. (2022). Making more with less: confinement effects for more sustainable chemical transformations. Green Chem., *24*, 1404–1438. https://doi.org/10.1039/D1GC03283F

3. Rebek, J. (2009). Molecular behavior in small spaces. Acc. Chem. Res., *42*, 1660–1668. https://doi.org/10.1021/ar9001203

4. Puthiaraj, P., Lee, Y. R., Zhang, S., & Ahn, W. S. (2016). Triazine-based covalent organic polymers: Design, synthesis and applications in heterogeneous catalysis. J. Mater. Chem. A, *4*, 16288–16311. https://doi.org/10.1039/c6ta06089g

5. Reactivity in Confined Spaces. (2021). In G. Lloyd; & R. S. Forgan (Eds.), *Reactivity in Confined Spaces*. The Royal Society of Chemistry. https://doi.org/10.1039/9781788019705

6. Liu, W., & Stoddart, J. F. (2021). Emergent behavior in nanoconfined molecular containers. Chem, *7*, 919–947. https://doi.org/10.1016/J.CHEMPR.2021.02.016

7. Warmuth, R. (2000). The inner phase of molecular container compounds as a novel reaction environment. J. Incl. Phenom., *37*, 1–37. https://doi.org/10.1023/A:1008082732631/METRICS

8. Montà-González, G., Sancenón, F., Martínez-Máñez, R., & Martí-Centelles, V. (2022). Purely Covalent Molecular Cages and Containers for Guest Encapsulation. Chem. Rev., *122*, 13636–13708. https://doi.org/10.1021/acs.chemrev.2c00198

9. Fang, Y., Powell, J. A., Li, E., Wang, Q., Perry, Z., Kirchon, A., Yang, X., Xiao, Z., Zhu, C., Zhang, L., Huang, F., & Zhou, H.-C. (2019). Catalytic reactions within the cavity of coordination cages. Chem. Soc. Rev., *48*, 4707–4730. https://doi.org/10.1039/C9CS00091G

10. Spicer, R. L., & Lusby, P. J. (2021). Chapter 2: Catalytic Strategies within the Confined Spaces of Coordination Cages. In *Monographs in Supramolecular Chemistry* (Vols. 2021-Janua, Issue 31, pp. 29–69). Royal Society of Chemistry. https://doi.org/10.1039/9781788019705-00029

11. Zhang, D., Ronson, T. K., Zou, Y. Q., & Nitschke, J. R. (2021). Metal–organic cages for molecular separations. Nat. Rev. Chem. 2021 53, *5*, 168–182. https://doi.org/10.1038/s41570-020-00246-1

12. Mitra, T., Jelfs, K. E., Schmidtmann, M., Ahmed, A., Chong, S. Y., Adams, D. J., & Cooper, A. I. (2013). Molecular shape sorting using molecular organic cages. Nat. Chem., *5*, 276–281. https://doi.org/10.1038/nchem.1550

13. Zhang, C., Wang, Q., Long, H., & Zhang, W. (2011). A highly C 70 selective shape-persistent rectangular prism constructed through one-step alkyne metathesis. J. Am. Chem. Soc., *133*, 20995–21001. https://doi.org/10.1021/JA210418T/SUPPL_FILE/JA210418T_SI_001.PDF

14. Ahmad, N., Younus, H. A., Chughtai, A. H., & Verpoort, F. (2014). Metal–organic molecular cages: applications of biochemical implications. Chem. Soc. Rev., *44*, 9–25. https://doi.org/10.1039/C4CS00222A

15. Cram, D. J., Tanner, M. E., & Thomas, R. (1991). The Taming of Cyclobutadiene. Angew. Chem. Int. Ed. Engl., *30*, 1024–1027. https://doi.org/10.1002/ANIE.199110241

16. Qiu, L., McCaffrey, R., Jin, Y., Gong, Y., Hu, Y., Sun, H., Park, W., & Zhang, W. (2018). Cage-templated synthesis of highly stable palladium nanoparticles and their catalytic activities in Suzuki–Miyaura coupling. Chem. Sci., *9*, 676–680. https://doi.org/10.1039/C7SC03148C

17. Rowan, S. J., Cantrill, S. J., Cousins, G. R. L., Sanders, J. K. M., & Stoddart, J. F. (2002). Dynamic Covalent Chemistry. Angew. Chem. Int. Ed., *41*, 898–952. https://doi.org/10.1002/1521-3773(20020315)41:6%3C898::AID-ANIE898%3E3.0.CO;2-E

18. Jin, Y., Yu, C., Denman, R. J., & Zhang, W. (2013). Recent advances in dynamic covalent chemistry. Chem. Soc. Rev., *42*, 6634–6654. https://doi.org/10.1039/C3CS60044K

19. Gropp, C., Canossa, S., Wuttke, S., Gándara, F., Li, Q., Gagliardi, L., & Yaghi, O. M. (2020). Standard Practices of Reticular Chemistry. ACS Cent. Sci., *6*, 1255–1273. https://doi.org/10.1021/ACSCENTSCI.0C00592/ASSET/IMAGES/LARGE/OC0C00592_0008.JPEG

20. Hasell, T., & Cooper, A. I. (2016). Porous organic cages: Soluble, modular and molecular pores. Nat. Rev. Mater., *1*, 1–14. https://doi.org/10.1038/natrevmats.2016.53

21. Pullen, S., Tessarolo, J., & Clever, G. H. (2021). Increasing structural and functional complexity in self-assembled coordination cages. Chem. Sci., *12*, 7269–7293. https://doi.org/10.1039/D1SC01226F

22. Otte, M., Lutz, M., & Klein Gebbink, R. J. M. (2017). Selective Synthesis of Hetero-Sequenced Aza-Cyclophanes. Eur. J. Org. Chem., *2017*, 1657–1661. https://doi.org/10.1002/EJOC.201700106

23. Lewis, J. E. M. (2022). Pseudo-heterolepticity in Low-Symmetry Metal-Organic Cages. Angew. Chem. Int. Ed., *61*, e202212392. https://doi.org/10.1002/ANIE.202212392

24. Xu, W., Tu, B., Liu, Q., Shu, Y., Liang, C. C., Diercks, C. S., Yaghi, O. M., Zhang, Y. B., Deng, H., & Li, Q. (2020). Anisotropic reticular chemistry. Nat. Rev. Mater., *5*, 764–779. https://doi.org/10.1038/s41578-020-0225-x

25. Lewis, J. E. M., Tarzia, A., White, A. J. P., & Jelfs, K. E. (2020). Conformational control of Pd2L4 assemblies with unsymmetrical ligands. Chem. Sci., *11*, 677–683. https://doi.org/10.1039/c9sc05534g

26. Yu, Y., Yang, J. M., & Rebek, J. (2020). Molecules in Confined Spaces: Reactivities and Possibilities in Cavitands. Chem, *6*, 1265–1274. https://doi.org/10.1016/J.CHEMPR.2020.04.014

27. Zhang, Q., Catti, L., & Tiefenbacher, K. (2018). Catalysis inside the Hexameric Resorcinarene Capsule. Acc. Chem. Res., *51*, 2107–2114. https://doi.org/https://doi.org/10.1021/acs.accounts.8b00320

28. Pappalardo, A., Puglisi, R., & Sfrazzetto, G. T. (2019). Catalysis inside supramolecular capsules: Recent developments. Catalysts, *9*, 630. https://doi.org/10.3390/catal9070630

29. Tateishi, T., Yoshimura, M., Tokuda, S., Matsuda, F., Fujita, D., & Furukawa, S. (2022). Coordination/metal–organic cages inside out. Coord. Chem. Rev., *467*, 214612. https://doi.org/10.1016/J.CCR.2022.214612

30. Percástegui, E. G., Ronson, T. K., & Nitschke, J. R. (2020). Design and Applications of Water-Soluble Coordination Cages. Chem. Rev., *120*, 13480–13544. https://doi.org/10.1021/acs.chemrev.0c00672

31. Chen, S., & Chen, L. J. (2022). Metal–Organic Cages: Applications in Organic Reactions. Chemistry, *4*, 494–519. https://doi.org/10.3390/chemistry4020036

32. Mastalerz, M. (2018). Porous Shape-Persistent Organic Cage Compounds of Different Size, Geometry, and Function. Acc. Chem. Res., *51*, 2411–2422. https://doi.org/10.1021/acs.accounts.8b00298

33. Little, M. A., & Cooper, A. I. (2020). The Chemistry of Porous Organic Molecular Materials. Adv. Funct. Mater., *30*, 1909842. https://doi.org/10.1002/adfm.201909842

34. Briggs, M. E., & Cooper, A. I. (2017). A perspective on the synthesis, purification, and characterization of porous organic cages. Chem. Mater., *29*, 149–157. https://doi.org/10.1021/acs.chemmater.6b02903

35. Acharyya, K., & Mukherjee, P. S. (2019). Organic Imine Cages: Molecular Marriage and Applications. Angew. Chem. Int. Ed., *58*, 8640–8653. https://doi.org/10.1002/anie.201900163

36. Huang, S., Lei, Z., Jin, Y., & Zhang, W. (2021). By-design molecular architectures via alkyne metathesis. Chem. Sci., *12*, 9591–9606. https://doi.org/10.1039/D1SC01881G

37. Pearcy, A. C., Lisboa, L. S., Preston, D., Page, N. B., Lawrence, T., Wright, L. J., Hartinger, C. G.,

& Crowley, J. D. (2023). Exploiting reduced-symmetry ligands with pyridyl and imidazole donors to construct a second-generation stimuli-responsive heterobimetallic [PdPtL4]4+ cage. Chem. Sci., *14*, 8615–8623. https://doi.org/10.1039/D3SC01354E

38. Hardy, M., & Lützen, A. (2020). Better Together: Functional Heterobimetallic Macrocyclic and Cage-like Assemblies. Chem. – A Eur. J., *26*, 13332–13346. https://doi.org/10.1002/CHEM.202001602

39. Lewis, J. E. M., & Crowley, J. D. (2020). Metallo-Supramolecular Self-Assembly with Reduced-Symmetry Ligands. Chempluschem, *85*, 815–827. https://doi.org/10.1002/CPLU.202000153

40. Tripathy, D., Debata, N. B., Naik, K. C., & Sahoo, H. S. (2022). Coordination driven discrete metallopolygons and cages from unsymmetric bidentate ligands. Coord. Chem. Rev., *456*, 214396. https://doi.org/10.1016/J.CCR.2021.214396

41. McTernan, C. T., Davies, J. A., & Nitschke, J. R. (2022). Beyond Platonic: How to Build Metal-Organic Polyhedra Capable of Binding Low-Symmetry, Information-Rich Molecular Cargoes. In *Chemical Reviews* (Vol. 122, Issue 11). https://doi.org/10.1021/acs.chemrev.1c00763

42. Davis, A. V., Fiedler, D., Ziegler, M., Terpin, A., & Raymond, K. N. (2007). Resolution of chiral, tetrahedral M4L6 metal-ligand hosts. J. Am. Chem. Soc., *129*, 15354–15363. https://doi.org/10.1021/ja0764815

43. Meng, W., Ronson, T. K., & Nitschke, J. R. (2013). Symmetry breaking in self-assembled M4L6 cage complexes. Proc. Natl. Acad. Sci. U. S. A., *110*, 10531–10535. https://doi.org/10.1073/pnas.1302683110

44. Wu, K., Benchimol, E., Baksi, A., & Clever, G. H. (2024). Non-statistical assembly of multicomponent [Pd2ABCD] cages. Nat. Chem. 2024, 1–8. https://doi.org/10.1038/s41557-023-01415-7

45. Greenaway, R. L., Santolini, V., Bennison, M. J., Alston, B. M., Pugh, C. J., Little, M. A., Miklitz, M., Eden-Rump, E. G. B., Clowes, R., Shakil, A., Cuthbertson, H. J., Armstrong, H., Briggs, M. E., Jelfs, K. E., & Cooper, A. I. (2018). High-throughput discovery of organic cages and catenanes using computational screening fused with robotic synthesis. Nat. Commun., *9*, 1–11. https://doi.org/10.1038/s41467-018-05271-9

46. Greenaway, R. L., & Jelfs, K. E. (2020). High-Throughput Approaches for the Discovery of Supramolecular Organic Cages. Chempluschem, *85*, 1813–1823. https://doi.org/10.1002/cplu.202000445

47. Slater, A. G., Little, M. A., Briggs, M. E., Jelfs, K. E., & Cooper, A. I. (2018). A solution-processable dissymmetric porous organic cage. Mol. Syst. Des. Eng., *3*, 223–227. https://doi.org/10.1039/c7me00090a

48. Santolini, V., Miklitz, M., Berardo, E., & Jelfs, K. E. (2017). Topological landscapes of porous organic cages. Nanoscale, *9*, 5280–5298. https://doi.org/10.1039/C7NR00703E

49. Acharyya, K., Mukherjee, S., & Mukherjee, P. S. (2013). Molecular marriage through partner preferences in covalent cage formation and cage-to-cage transformation. J. Am. Chem. Soc., *135*, 554–557. https://doi.org/10.1021/ja310083p

50. Acharyya, K., & Mukherjee, P. S. (2015). Shape and size directed self-selection in organic cage formation. Chem. Commun., *51*, 4241–4244. https://doi.org/10.1039/C5CC00075K

51. Hasell, T., Little, M. A., Chong, S. Y., Schmidtmann, M., Briggs, M. E., Santolini, V., Jelfs, K. E., & Cooper, A. I. (2017). Chirality as a tool for function in porous organic cages. Nanoscale, *9*, 6783–6790. https://doi.org/10.1039/c7nr01301a

52. Zhang, L., Jin, Y., Tao, G. H., Gong, Y., Hu, Y., He, L., & Zhang, W. (2020). Desymmetrized Vertex Design toward a Molecular Cage with Unusual Topology. Angew. Chem. Int. Ed., *59*, 20846–20851. https://doi.org/10.1002/ANIE.202007454

53. Berardo, E., Greenaway, R. L., Turcani, L., Alston, B. M., Bennison, M. J., Miklitz, M., Clowes, R., Briggs, M. E., Cooper, A. I., & Jelfs, K. E. (2018). Computationally-inspired discovery of an

unsymmetrical porous organic cage. Nanoscale, *10*, 22381–22388. https://doi.org/10.1039/c8nr06868b

54. Abet, V., Szczypiński, F. T., Little, M. A., Santolini, V., Jones, C. D., Evans, R., Wilson, C., Wu, X., Thorne, M. F., Bennison, M. J., Cui, P., Cooper, A. I., Jelfs, K. E., & Slater, A. G. (2020). Inducing Social Self-Sorting in Organic Cages To Tune The Shape of The Internal Cavity. Angew. Chem. Int. Ed., *59*, 16755–16763. https://doi.org/10.1002/anie.202007571

55. Greenaway, R. L., Santolini, V., Bennison, M. J., Alston, B. M., Pugh, C. J., Little, M. A., Miklitz, M., Eden-Rump, E. G. B., Clowes, R., Shakil, A., Cuthbertson, H. J., Armstrong, H., Briggs, M. E., Jelfs, K. E., & Cooper, A. I. (2018). High-throughput discovery of organic cages and catenanes using computational screening fused with robotic synthesis. Nat. Commun., *9*, 1–11. https://doi.org/10.1038/s41467-018-05271-9

56. Berardo, E., Turcani, L., Miklitz, M., & Jelfs, K. E. (2018). An evolutionary algorithm for the discovery of porous organic cages. Chem. Sci., *9*, 8513–8527. https://doi.org/10.1039/c8sc03560a

57. Teng, B., Little, M. A., Hasell, T., Chong, S. Y., Jelfs, K. E., Clowes, R., Briggs, M. E., & Cooper, A. I. (2019). Synthesis of a large, shape-flexible, solvatomorphic porous organic cage. Cryst. Growth Des., *19*, 3647–3651. https://doi.org/10.1021/acs.cgd.8b01761

58. Roberts, D. A., Pilgrim, B. S., & Nitschke, J. R. (2018). Covalent post-assembly modification in metallosupramolecular chemistry. Chem. Soc. Rev., *47*, 626–644. https://doi.org/10.1039/C6CS00907G

59. Otte, M. (2023). Reactions in Endohedral Functionalized Cages. Eur. J. Org. Chem., *26*, e202300012. https://doi.org/10.1002/ejoc.202300012

60. Schneider, M. W., Oppel, I. M., Griffin, A., & Mastalerz, M. (2013). Post-Modification of the Interior of Porous Shape-Persistent Organic Cage Compounds. Angew. Chem. Int. Ed., *52*, 3611–3615. https://doi.org/10.1002/anie.201208156

61. Bhat, A. S., Elbert, S. M., Zhang, W.-S., Rominger, F., Dieckmann, M., Schröder, R. R., & Mastalerz, M. (2019). Transformation of a [4+6] Salicylbisimine Cage to Chemically Robust Amide Cages. Angew. Chem. Int. Ed., *58*, 8819–8823. https://doi.org/10.1002/anie.201903631

62. Wang, H., Jin, Y., Sun, N., Zhang, W., & Jiang, J. (2021). Post-synthetic modification of porous organic cages. Chem. Soc. Rev., *50*, 8874–8886. https://doi.org/10.1039/D0CS01142H

63. Andrews, K. G., & Christensen, K. E. (2023). Access to Amide-linked Organic Cages by in situ Trapping of Metastable Imine Assemblies: Solution Phase Bisamine Recognition. Chem. – A Eur. J., e202300063. https://doi.org/10.1002/chem.202300063

64. Waller, P. J., Lyle, S. J., Osborn Popp, T. M., Diercks, C. S., Reimer, J. A., & Yaghi, O. M. (2016). Chemical Conversion of Linkages in Covalent Organic Frameworks. J. Am. Chem. Soc., *138*, 15519–15522. https://doi.org/10.1021/jacs.6b08377

65. Han, X., Huang, J., Yuan, C., Liu, Y., & Cui, Y. (2018). Chiral 3D Covalent Organic Frameworks for High Performance Liquid Chromatographic Enantioseparation. J. Am. Chem. Soc., *140*, 892–895. https://doi.org/10.1021/jacs.7b12110

66. Lauer, J. C., Bhat, A. S., Barwig, C., Fritz, N., Kirschbaum, T., Rominger, F., & Mastalerz, M. (2022). [2+3] Amide Cages by Oxidation of [2+3] Imine Cages – Revisiting Molecular Hosts for Highly Efficient Nitrate Binding. Chem. Eur. J., *28*, e202201527. https://doi.org/10.1002/chem.202201527

67. Ganz, T. (2006). Lysozyme. In *Encyclopedia of Respiratory Medicine, Four-Volume Set* (pp. 649–653). Academic Press. https://doi.org/10.1016/B0-12-370879-6/00228-3

68. Bugg, T. D. H. (2012). Introduction to Enzyme and Coenzyme Chemistry: Third Edition. In *Introduction to Enzyme and Coenzyme Chemistry: Third Edition*. John Wiley and Sons. https://doi.org/10.1002/9781118348970

69. Pros, G. J., & Bloomfield, A. J. (2019). Why Do N-Alkylated Anilides Bend Over? the Factors

Dictating the Divergent Conformational Preferences of 2° and 3° N-Aryl Amides. J. Phys. Chem. A, *123*, 7609–7618. https://doi.org/10.1021/acs.jpca.9b04555

70. Saraogi, I., Incarvito, C. D., & Hamilton, A. D. (2008). Controlling curvature in a family of oligoamide α-helix mimetics. Angew. Chem. Int. Ed., *47*, 9691–9694. https://doi.org/10.1002/anie.200803778

71. Bell, T. W., Catalano, V. J., Drew, M. G. B., & Phillips, D. J. (2002). Bond angle versus torsional deformation in an overcrowded alkene: 9-(2,2,2-Triphenylethylidene)fluorene. Chem. Eur. J., *8*, 5001–5006. https://doi.org/10.1002/1521-3765(20021104)8:21<5001::AID-CHEM5001>3.0.CO;2-A

72. Armon, A. M., Bedi, A., Borin, V., Schapiro, I., & Gidron, O. (2021). Bending versus Twisting Acenes – A Computational Study. Eur. J. Org. Chem., *2021*, 5424–5429. https://doi.org/10.1002/ejoc.202100865

73. Gellman, S. H. (1998). Foldamers: A Manifesto. Acc. Chem. Res., *31*, 173–180. https://doi.org/10.1021/ar960298r

74. Yuan, L., Zeng, H., Yamato, K., Sanford, A. R., Feng, W., Atreya, H. S., Sukumaran, D. K., Szyperski, T., & Gong, B. (2004). Helical aromatic oligoamides: Reliable, readily predictable folding from the combination of rigidified structural motifs. J. Am. Chem. Soc., *126*, 16528–16537. https://doi.org/10.1021/ja046858w

75. Schneider, M. W., Oppel, I. M., & Mastalerz, M. (2012). Exo-Functionalized Shape-Persistent [2+3] Cage Compounds: Influence of Molecular Rigidity on Formation and Permanent Porosity. Chem. Eur. J., *18*, 4156–4160. https://doi.org/10.1002/chem.201200032

76. Ollerton, K., Greenaway, R. L., & Slater, A. G. (2021). Enabling Technology for Supramolecular Chemistry. Front. Chem., *9*, 973. https://doi.org/10.3389/fchem.2021.774987

77. Slater, A. G., Reiss, P. S., Pulido, A., Little, M. A., Holden, D. L., Chen, L., Chong, S. Y., Alston, B. M., Clowes, R., Haranczyk, M., Briggs, M. E., Hasell, T., Day, G. M., & Cooper, A. I. (2017). Computationally-Guided Synthetic Control over Pore Size in Isostructural Porous Organic Cages. ACS Cent. Sci., *3*, 734–742. https://doi.org/10.1021/acscentsci.7b00145

78. Miklitz, M., & Jelfs, K. E. (2018). pywindow: Automated Structural Analysis of Molecular Pores. J. Chem. Inf. Model., *58*, 2387–2391. https://doi.org/10.1021/acs.jcim.8b00490

79. Reiss, P. S., Little, M. A., Santolini, V., Chong, S. Y., Hasell, T., Jelfs, K. E., Briggs, M. E., & Cooper, A. I. (2016). Periphery-Functionalized Porous Organic Cages. Chem. Eur. J., *22*, 16547–16553. https://doi.org/10.1002/chem.201603593

80. Pullen, S., Tessarolo, J., & Clever, G. H. (2021). Increasing structural and functional complexity in self-assembled coordination cages. Chem. Sci., *12*, 7269–7293. https://doi.org/10.1039/D1SC01226F

81. Dasary, H., Jagan, R., & Chand, D. K. (2018). Ligand Isomerism in Coordination Cages. Inorg. Chem., *57*, 12222–12231. https://doi.org/10.1021/acs.inorgchem.8b01884

82. Sarkar, M., Dasary, H., & Chand, D. K. (2021). Helicity induction by innocent anion in a quadruple stranded cage. J. Organomet. Chem., *950*, 121984. https://doi.org/10.1016/j.jorganchem.2021.121984

83. Mishra, S. S., Kompella, S. V. K., Krishnaswamy, S., Balasubramanian, S., & Chand, D. K. (2020). Low-Symmetry Self-Assembled Coordination Complexes with Exclusive Diastereoselectivity: Experimental and Computational Studies. Inorg. Chem., *59*, 12884–12894. https://doi.org/10.1021/acs.inorgchem.0c01964

84. Skowronek, P., Warzajtis, B., Rychlewska, U., & Gawroński, J. (2013). Self-assembly of a covalent organic cage with exceptionally large and symmetrical interior cavity: the role of entropy of symmetry. Chem. Commun., *49*, 2524–2526. https://doi.org/10.1039/C3CC39098E

85. Wiberg, K. B. (1986). The Concept of Strain in Organic Chemistry. Angew. Chem. Int. Ed. Engl., *25*, 312–322. https://doi.org/10.1002/anie.198603121

86. Shiotari, A., Nakae, T., Iwata, K., Mori, S., Okujima, T., Uno, H., Sakaguchi, H., & Sugimoto, Y. (2017). Strain-induced skeletal rearrangement of a polycyclic aromatic hydrocarbon on a copper surface. Nat. Commun., *8*, 1–8. https://doi.org/10.1038/ncomms16089

87. Safont-Sempere, M. M., Fernández, G., & Würthner, F. (2011). Self-sorting phenomena in complex supramolecular systems. Chem. Rev., *111*, 5784–5814. https://doi.org/10.1021/cr100357h

88. Serrano-Molina, D., Montoro-García, C., Mayoral, M. J., De Juan, A., & González-Rodríguez, D. (2022). Self-Sorting Governed by Chelate Cooperativity. J. Am. Chem. Soc., *144*, 5450–5460. https://doi.org/10.1021/jacs.1c13295

89. Coles, S. J., Allan, D. R., Beavers, C. M., Teat, S. J., Holgate, S. J. W., & Tovee, C. A. (2020). Leading edge chemical crystallography service provision and its impact on crystallographic data science in the twenty-first century. In *Structure and Bonding* (Vol. 185, pp. 69–140). Springer Science and Business Media Deutschland GmbH. https://doi.org/10.1007/430_2020_63

90. Richards, A. (2015). University of Oxford Advanced Research Computing. Univ. Oxford Adv. Res. Comput., *783*, 1–4. https://doi.org/10.5281/ZENODO.22558
